# Gene-based burden scores identify rare variant associations for 28 blood biomarkers

**DOI:** 10.1186/s12863-023-01155-0

**Published:** 2023-09-04

**Authors:** Rana Aldisi, Emadeldin Hassanin, Sugirthan Sivalingam, Andreas Buness, Hannah Klinkhammer, Andreas Mayr, Holger Fröhlich, Peter Krawitz, Carlo Maj

**Affiliations:** 1 Institute of Genomic Statistic and Bioinformatics, University Hospital Bonn, Bonn, Germany; 2 Luxembourg Center for Systems Biomedicine, University of Luxembourg, Esch-Sur-Alzette, Luxembourg; 3 Core Unit for Bioinformatics Analysis, University Hospital Bonn, Bonn, Germany; 4 Institute of Medical Biometry, Informatics and Epidemiology, University Hospital Bonn, Bonn, Germany; 5 Fraunhofer Institute for Algorithms and Scientific Computing, Sankt Augustin, Germany; 6 Bonn-Aachen International Center for IT (b-it), University of Bonn, Bonn, Germany; 7 Centre for Human Genetics, University of Marburg, Marburg, Germany

**Keywords:** Gene associations, Blood biomarkers, Genetic prediction, Rare variants, PRS, Complex phenotypes

## Abstract

**Background:**

A relevant part of the genetic architecture of complex traits is still unknown; despite the discovery of many disease-associated common variants. Polygenic risk score (PRS) models are based on the evaluation of the additive effects attributable to common variants and have been successfully implemented to assess the genetic susceptibility for many phenotypes. In contrast, burden tests are often used to identify an enrichment of rare deleterious variants in specific genes. Both kinds of genetic contributions are typically analyzed independently. Many studies suggest that complex phenotypes are influenced by both low effect common variants and high effect rare deleterious variants. The aim of this paper is to integrate the effect of both common and rare functional variants for a more comprehensive genetic risk modeling.

**Methods:**

We developed a framework combining gene-based scores based on the enrichment of rare functionally relevant variants with genome-wide PRS based on common variants for association analysis and prediction models. We applied our framework on UK Biobank dataset with genotyping and exome data and considered 28 blood biomarkers levels as target phenotypes. For each biomarker, an association analysis was performed on full cohort using gene-based scores (GBS). The cohort was then split into 3 subsets for PRS construction and feature selection, predictive model training, and independent evaluation, respectively. Prediction models were generated including either PRS, GBS or both (combined).

**Results:**

Association analyses of the cohort were able to detect significant genes that were previously known to be associated with different biomarkers. Interestingly, the analyses also revealed heterogeneous effect sizes and directionality highlighting the complexity of the blood biomarkers regulation. However, the combined models for many biomarkers show little or no improvement in prediction accuracy compared to the PRS models.

**Conclusion:**

This study shows that rare variants play an important role in the genetic architecture of complex multifactorial traits such as blood biomarkers. However, while rare deleterious variants play a strong role at an individual level, our results indicate that classical common variant based PRS might be more informative to predict the genetic susceptibility at the population level.

**Supplementary Information:**

The online version contains supplementary material available at 10.1186/s12863-023-01155-0.

## Background

The genetic architecture of complex phenotypes has been studied extensively for over a century; however, a relevant part of the genetics still elude us. That is because, essentially, many factors are involved in the development of such traits, both biological and environmental, which makes it harder to discover causative effects for any complex phenotype or disease [1]. Genome-wide association studies (GWAS) investigate the associations of low-effect single nucleotide polymorphisms (SNPs) with specific phenotypes. For the last decade, GWAS have been used to identify many common variants that are associated with diseases and other phenotypes such as cancer [2], autism [3] and cholesterol [4]. About 90% of the variants identified by GWAS are located in the non-coding regions of the genome. This gives insight to the mechanisms behind development and progress of complex phenotypes by exploring regulatory elements that could have an effect on disease related genes [5]. However, the narrow sense of heritability estimated from the GWAS, also known as SNP-h^2^, is typically lower than the broad sense of heritability H^2^ estimate from twins and family studies, this is known as the missing heritability [6]. Different hypotheses have been suggested to resolve the difference between observed and measured heritability, such as non-linear effects, epigenetics and rare variants [6]. It has also been hypothesized that family studies or twin studies might have overestimated the heritability and that the shared environment plays a significant role in these traits [7]. On the other hand, many studies suggest that more genetic variations need to be included in the analysis of complex traits to account for the unexplained heritability, such as small to moderate effect low-frequency (MAF1%-5%) variants, and potentially highly damaging rare variants (MAF < 1%) [8]. In fact, it has been observed that rare variants contribute to the genetic landscape of complex phenotypes such as inflammatory bowel disease [9], hypertension [10] and autism [11].

Common and rare variants are typically analyzed independently. Common variants’ effects on a certain phenotype are analyzed using polygenic risk scores (PRS), these scores are usually derived from large-scale GWAS and are used to assess an individual’s genetic liability for a certain trait or disease [12]. However, current PRSs explain only a small part of the heritability of complex traits [13]. On the other hand, multiple methods have been developed to find phenotype associations with rare variants. A widely known category is burden test, which collapses all information in a genetic region (e.g. gene) into one genetic burden score that can be used for association analysis. The association is then analyzed between the burden score and a certain phenotype. However, burden tests assume that all rare variants are causal and have the same directional effect on the trait tested [14]. Another class of methods was developed to avoid these limitations, which is known as the variance-component tests. These tests analyze associations by looking at joint genetic effect for variants in a genetic region. For example, sequence kernel association test (SKAT), aggregates score statistics of multiple variants then evaluates the distribution [15]. While this class has dealt with the limitations of burden tests, it might not perform well when a large proportion of the variants have strong effects in the same direction [14]. For this purpose, methods combining burden tests and variance-component tests have been proposed. One of these methods is SKAT-O, an extension of SKAT which can incorporate both common and rare variants in the analysis [16]. While all these different approaches have their advantages, one of their disadvantages is that they do not provide individual-level data, therefore, other methods based on functional annotations and frequency weight have been developed, such as Genepy [17] and GenRisk [18]. These approaches are more general and allow gene-based scores at individuals levels to be derived which can be used subsequently for multiple analyses.

For both common and rare variants, well-established methods exist to perform genotype-phenotype association and prediction analysis; however, their combined contributions have not been fully studied. Our paper aims to analyze the contribution of both rare and common variants to complex phenotypes. We achieve this by integrating gene-based scores for rare variants and PRS for common variants in genetic risk modeling.

## Results

We used gene-based scores, calculated based on the burden of rare functional variants and allele frequency, to analyze gene associations with 28 quantitative biomarkers. We further integrated the gene-based scores with the PRS models, aiming to enhance the risk prediction.

### Identification of phenotype-associated genes

**Fig. 1:**
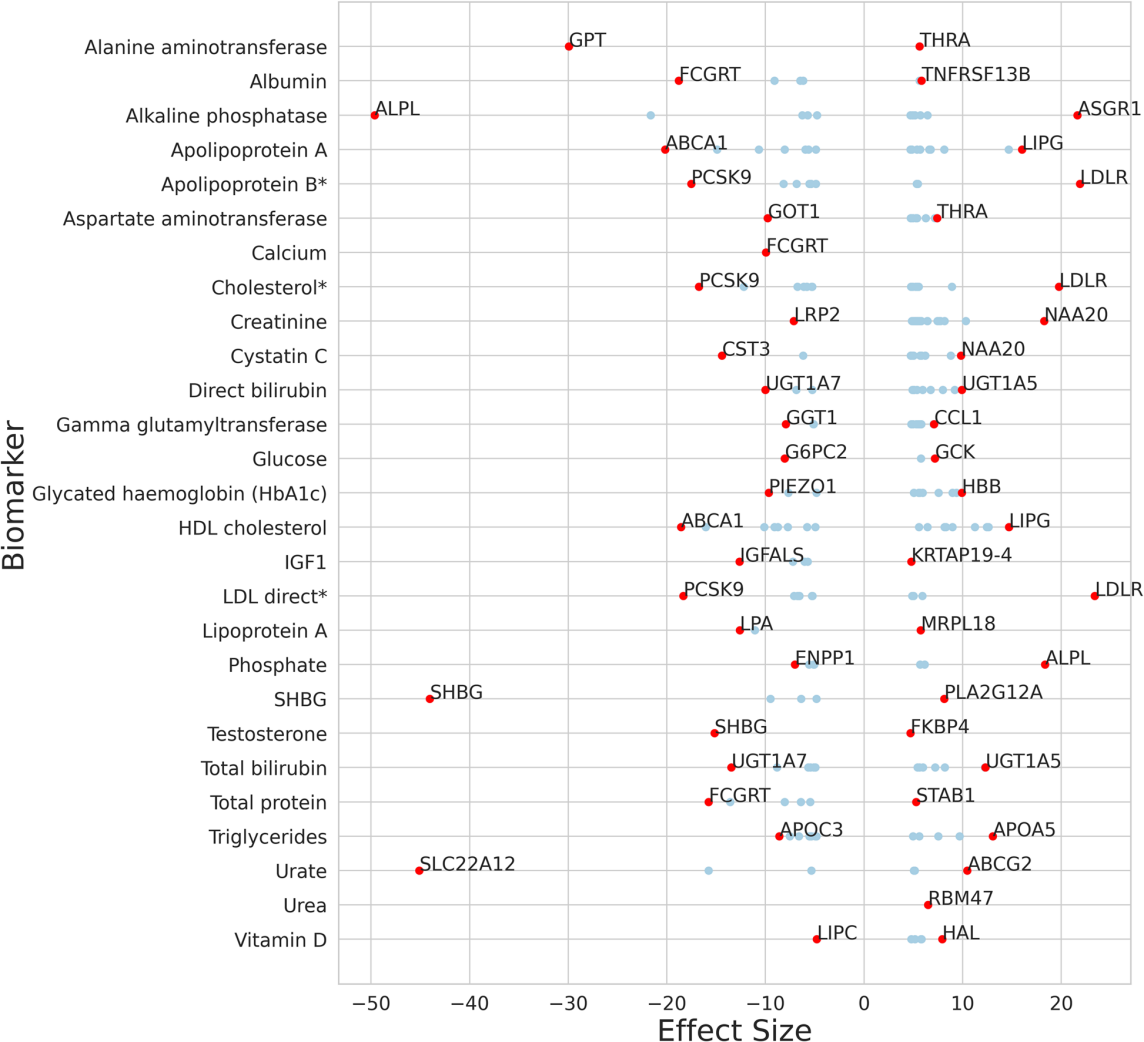
Distribution of effect sizes of genes with *P*-value < 0.05 after Bonferroni correction, the highest and lowest genes’ effect sizes are labeled for every biomarker

To identify genes associated with different biomarkers, we performed association analysis, using linear regression, on the UK biobank cohort with 28 blood biomarkers extracted as phenotypes. Furthermore, we calculated the effect size (z-score) of each gene on each biomaker phenotype using the beta coefficient and standard error extracted from the association analysis. Figure 1 displays the distribution of the effect sizes of genes with *P*-value < 0.05 after Bonferroni correction for each phenotype with highlight on the highest and lowest effect size genes, with effect sizes ranging between -49.6 (ALPL in alkaline phosphatase) and 23.4 (LDLR in LDL direct measurement). The number of genes with positive and negative effects for each biomarker is shown in Table 1.

**Table 1:** Number of significantly associated genes with negative and positive effect sizes

Biomarker	Negative effect	Positive effect
Alanine aminotransferase	1	1
Albumin	4	2
Alkaline phosphatase	5	6
Apolipoprotein A	7	9
Apolipoprotein B^a^	6	3
Aspartate aminotransferase	1	8
Cholesterol^a^	8	7
Creatinine	1	27
Cystatin C	2	9
Direct bilirubin	3	10
Gamma glutamyltransferase	3	9
Glucose	1	2
Glycated haemoglobin (HbA1c)	3	8
HDL cholesterol	8	9
IGF1	4	1
LDL direct^a^	7	4
Lipoprotein A	2	1
Phosphate	4	3
SHBG	4	1
Testosterone	1	1
Total bilirubin	7	7
Total protein	5	1
Triglycerides	8	5
Urate	3	3
Vitamin D	1	5

^a^ Values adjusted for statins

### Rare and common variants integrated risk prediction models

In order to assess the contribution of rare and common variants on complex phenotypes, we generated prediction models for each biomarker. These models were generated using GenRisk pipeline, which evaluates different regression models and outputs the model with the best performance as a final output, we then calculated the R^2^ for each model using an independent testing set. Four different models for each biomarker were generated: based on polygenic risk scores for common variant effect (PRS model); based on selected gene-based scores for rare variant effect (GBS model); combining both rare and common variant effects (PRS+GBS combined model); a only covariates-model (in order to assess the incremental performance due to the genetic factors). Table 2 presents the R^2^ for the covariates models and the incremental R^2^ for all other models in comparison.

**Table 2:** The R^2^ of prediction models for blood biomarkers, with calculated incremental R^2^ values between covariates only model and the rest of the models

Biomarker	Gene predictors	Covariates Model R^2^	Incremental R^2^		
			Genes	PRS	Combined
Alanine aminotransferase	4	0.137	0.003	0.011	0.014
Albumin	5	0.059	0.005	0.027	0.032
Alkaline Phosphatase	8	0.071	0.026	0.088	0.103
Apolipoprotein A	11	0.208	0.009	0.075	0.083
Apolipoprotein B^a^	5	0.088	0.007	0.157	0.162
Aspartate Aminotransferase	10	0.040	0.000	0.009	0.009
Calcium	2	0.028	0.002	0.017	0.018
Cholesterol^a^	6	0.089	0.006	0.096	0.099
C-reactive protein	5	0.066	0.004	0.009	0.011
Creatinine	41	0.248	-0.006	0.011	0.005
Cystatin C	11	0.177	-0.001	0.043	0.043
Direct bilirubin	14	0.045	0.011	0.272	0.272
Gamma glutamyltransferase	11	0.053	0.001	0.015	0.015
Glucose	8	0.030	0.001	0.003	0.003
Glycated haemoglobin (HbA1c)	16	0.098	0.001	0.020	0.022
HDL cholesterol	14	0.274	0.011	0.113	0.120
IGF1	5	0.091	0.003	0.067	0.070
LDL direct^a^	5	0.077	0.006	0.109	0.113
Lipoprotein A	3	0.000	0.003	0.567	0.591
Phosphate	3	0.067	0.003	0.020	0.023
SHBG	5	0.309	0.017	0.053	0.065
Testosterone	1	0.828	0.001	0.006	0.008
Total bilirubin	11	0.064	0.012	0.399	0.400
Total protein	4	0.003	0.005	0.039	0.042
Triglycerides	7	0.139	0.003	0.058	0.061
Urate	4	0.387	0.013	0.065	0.077
Urea	2	0.070	0.000	0.009	0.010
Vitamin D	2	0.040	0.001	0.015	0.015

^a^ Values adjusted for statins

## Discussion

In this study, we evaluated the association of rare genetic variants with 28 blood biomarkers. In addition, we explore the genetic contribution of these variants to the regulation of the biomarkers levels using samples from the UK Biobank. The association analysis, based on gene-scores derived from the burden of rare functional variants, revealed several interesting gene candidates associated with different blood biomarkers, showing both positive (increasing) and negative (decreasing) effect sizes. Some of these candidate genes have clear known associations with their respective biomarker; for example, ALPL gene was identified in association with alkaline phosphatase biomarker levels, and SHBG gene was associated with both sex hormone binding globulin (SHBG) and testosterone biomarkers’ levels. In addition, the negative effect direction of those associations indicates that the presence of rare functional, possibly damaging, variants, as measured by the gene-based scores, decreases the biomarkers’ levels. This is consistent with the fact that ALPL and SHBG are the protein-coding genes for the alkaline phosphatase and SHBG biomarkers, respectively. Consequently, the presence of damaging variants in these genes could lead to a decrease in the production of their corresponding biomarkers. Additionally, since SHBG regulates testosterone levels in the body, a reduction in SHBG levels may also result in a reduction of testosterone levels [19].

Another clear example for rare variant associations is LDL (low-density lipoprotein), which showed association and positive effect direction with LDLR and negative effect direction with PCSK9. In this case, damaging mutations in LDLR, the gene for the LDL receptor, result in an increase in LDL levels in plasma. This finding is not surprising, as it has been previously suggested that mutations in LDLR are often responsible for familial hypercholesterolemia [20]. Instead, PCSK9 is a regulatory protein that degrades LDLR and thus leads to an increase in LDL plasma levels. In fact, PCSK9 inhibitors have been used as a treatment for hypercholestrolemia [21].

To confirm and validate our result, we also compared our findings with two different approaches that try to find gene-phenotype associations using rare variants and are performed on UK biobank samples, genebass [22] and AstraZeneca PheWAS [23]. Genebass uses SAIGE-GENE [24] to perform gene-based burden test and SKAT-O, while AstraZeneca PheWAS analysis was performed using Fisher’s exact test on different models each with their own variant functional and allele frequency filtering criteria. In general, the different methods share many similar associations, however, our method has shown to have less inflated lambda in comparison to genebass. Typically, the lambda values are expected to be near 1, a lambda lower than 1 (deflation) could mean under-powered analysis and a lambda higher than 1 (inflation) could mean high false positive rate. Table 3 presents the lambdas as calculated from the three different approaches, since genebass and Astrazeneca PheWAS used different models to find associations, the average of these models is reported. Lambdas for all models’ values can be found in the supplementary material (Table S2).

**Table 3:** The lambdas of the three different approaches, averaged in case of multiple values. Full and detailed table with all values can be found in Supplementary material

Biomarker	GenRisk	Genebass Burden Average	Genebass SKATO Average	AstraZeneca PheWAS Average
Alanine aminotransferase	1.016	1.139 ± 0.081	1.1967 ± 0.278	1.046 ± 0.016
Albumin	1.069	1.136 ± 0.132	1.231 ± 0.243	1.050 ± 0.018
Alkaline phosphatase	1.078	1.322 ± 0.259	1.739 ± 1.130	1.084 ± 0.023
Apolipoprotein A	1.068	1.207 ± 0.170	1.340 ± 0.317	1.070 ± 0.020
Apolipoprotein B	1.105	1.149 ± 0.094	1.247 ± 0.289	1.053 ± 0.018
Aspartate aminotransferase	0.951	1.174 ± 0.104	1.253 ± 0.285	1.064 ± 0.027
Calcium	1.063	1.099 ± 0.045	1.162 ± 0.140	1.053 ± 0.020
Cholesterol	1.085	1.158 ± 0.117	1.199 ± 0.266	1.050 ± 0.014
C-reactive protein	0.995	1.228 ± 0.178	1.505 ± 0.786	1.082 ± 0.0201
Creatinine	0.861	1.201 ± 0.158	1.328 ± 0.418	1.097 ± 0.027
Cystatin C	0.995	1.221 ± 0.173	1.376 ± 0.371	1.093 ± 0.030
Direct bilirubin	0.993	1.168 ± 0.146	1.411 ± 0.613	1.036 ± 0.001
Gamma glutamyltransferase	0.965	1.207 ± 0.078	1.384 ± 0.289	1.065 ± 0.030
Gluscose	0.998	1.081 ± 0.081	1.082 ± 0.111	1.019 ± 0.013
Glycated haemoglobin HbA1c	1.018	1.224 ± 0.125	1.391 ± 0.387	1.090 ± 0.026
HDL Cholesterol	1.076	1.231 ± 0.175	1.417 ± 0.474	1.075 ± 0.026
IGF1	1.084	1.212 ± 0.145	1.352 ± 0.396	1.096 ± 0.019
LDL direct	1.092	1.132 ± 0.119	1.179 ± 0.245	1.039 ± 0.016
Lipoprotein A	0.992	1.156 ± 0.152	1.354 ± 0.534	1.020 ± 0.008
Phosphate	1.065	1.041 ± 0.028	0.976 ± 0.060	1.054 ± 0.020
SHBG	1.07	1.194 ± 0.076	1.353 ± 0.336	1.065 ± 0.025
Testosterone	1.005	1.088 ± 0.105	1.072 ± 0.205	1.016 ± 0.014
Total bilirubin	1.028	1.264 ± 0.193	1.648 ± 0.911	1.030 ± 0.013
Total protein	1.059	1.194 ± 0.183	1.286 ± 0.323	1.078 ± 0.021
Triglycerides	1.066	1.197 ± 0.187	1.279 ± 0.362	1.071 ± 0.011
Urate	1.076	1.227 ± 0.054	1.429 ± 0.335	1.058 ± 0.018
Urea	1.036	1.116 ± 0.091	1.157 ± 0.226	1.049 ± 0.012
Vitamin D	1.034	1.089 ± 0.016	1.133 ± 0.135	1.049 ± 0.019

**Fig. 2:**
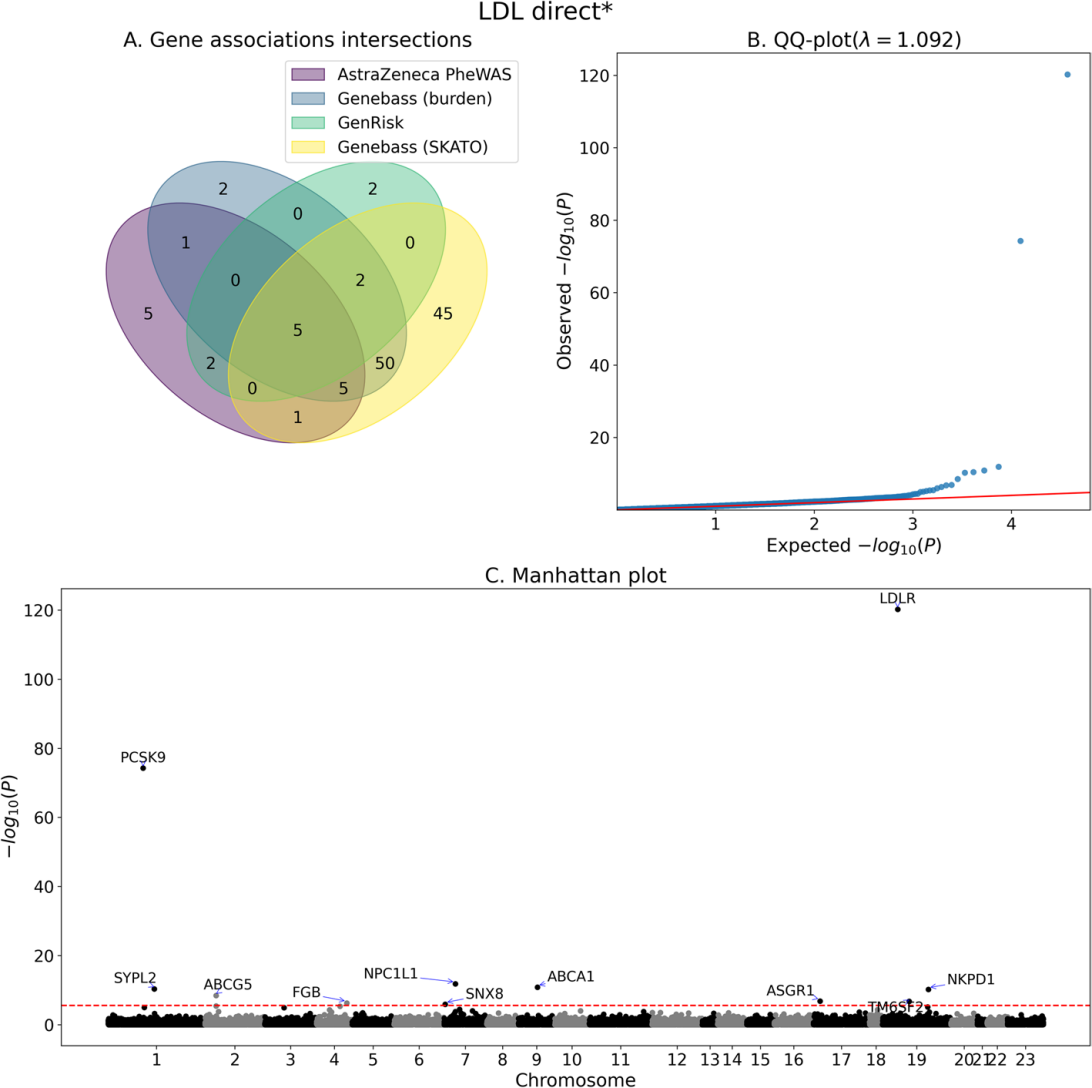
Association analysis summary for LDL direct*. **A** Venn diagram of the number significantly associated genes as identified by GenRisk, AstraZeneca PheWAS (all models) and genebass (Burden and SKATO). **B** QQ-plot of the *P*-values of GenRisk pipeline results. **C** Manhattan plot of GenRisk pipeline results. *statin adjusted values

**Fig. 3:**
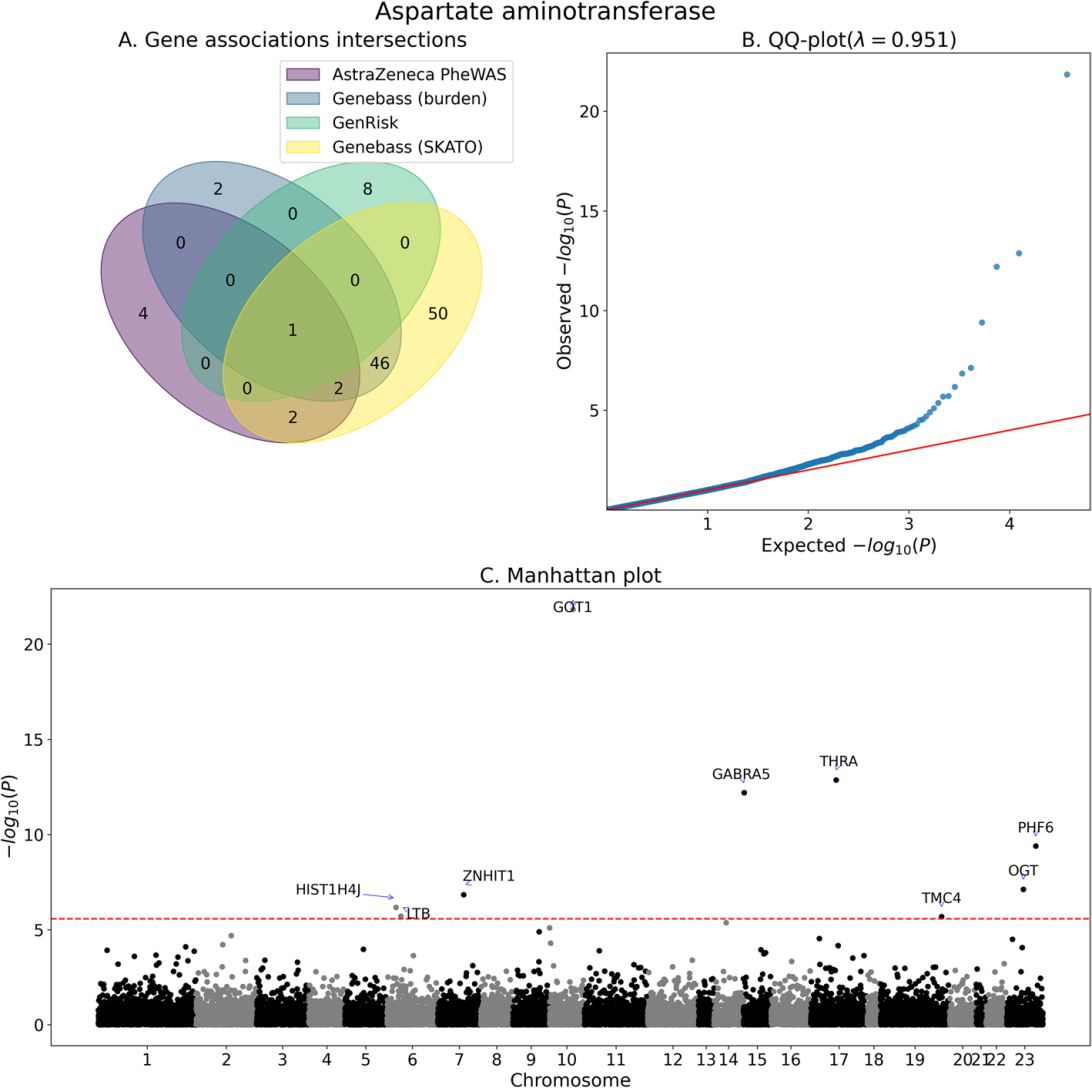
Association analysis summary for aspartate aminotransferase. **A** Venn diagram of the number significantly associated genes as identified by GenRisk, AstraZeneca PheWAS (all models) and genebass (Burden and SKATO). **B** QQ-plot of the *P*-values of GenRisk pipeline results. **C** Manhattan plot of GenRisk pipeline results

**Fig. 4:**
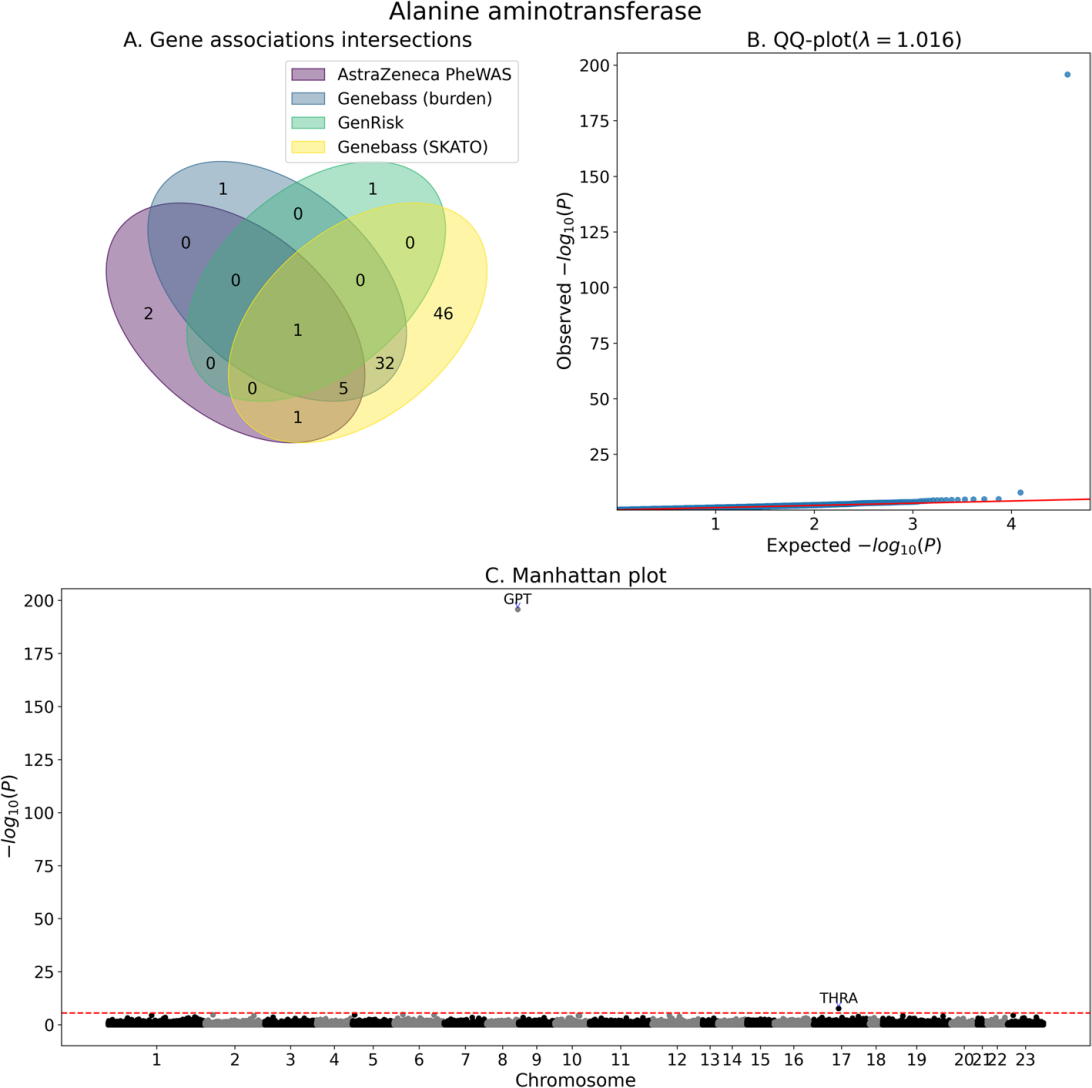
Association analysis summary for alanine aminotransferase. **A** Venn diagram of the number significantly associated genes as identified by GenRisk, AstraZeneca PheWAS (all models) and genebass (Burden and SKATO). **B** QQ-plot of the *P*-values of GenRisk pipeline results. **C** Manhattan plot of GenRisk pipeline results

All approaches identified genes that are previously known to be associated with the respective biomarker (*P*-value < 0.05 after Bonferroni correction), for example PCSK9, LDLR, NPC1L1 and ABCG5 association with LDL levels [25–27]. However, our approach was able to identify potential novel associations that were not found with the other methods, such as, SNX8 for LDL and cholesterol, which is a part of the sorting nexin family and have been previously associated with the distribution of neuronal cholesterol [28]. Another example of shared association among all approaches is the association of GOT1, also known as AST1, with aspartate aminotransferase (AST), which is the gene encoding AST. GenRisk further identified THRA, also known as thyroid hormone receptor alpha. AST is a liver enzyme that is used as a biomarker to indicate liver damage or disease and in fact, the liver plays an important role in the activation, metabolism and transport of thyroid hormone, while thyroid hormones are said to affect hepatic cells metabolism [29]. Notably, THRA was also identified by GenRisk as significant, for alanine aminotransferase, another liver biomarker. Figures 2, 3 and 4 display the association analysis results along with venn diagram representing the number of significant associations identified from each approach mentioned above for LDL, aspartate aminotransferase and alanine aminotransferase, respectively. Similar figures for the rest of the biomarkers are provided in the supplementary material (Figs. S2–S26). The summary statistics for the association analysis performed by GenRisk for each biomarker are also provided in the supplementary material (Tables S3–S31).

In addition, in order to assess the contribution of rare-variants in the 28 blood biomarkers, we compared risk prediction models using four different modalities (see Methods for details). Our prediction model results suggest that the effect of rare variants on complex phenotypes differs depending on the distinct genetic architecture of the phenotypes. Furthermore, even though most of the biomarkers predictions show improvements when combining rare (GBS) and common (PRS) variants, these improvements are marginal in many cases which suggest that the added predictive value of rare variants in risk prediction is limited. Interestingly, gradient boosting regressor was selected by our pipeline as best performing model for most biomarkers. In gradient boosting machines, weak performing models, e.g decision trees, are combined together to generate a more powerful predictive model [30]. In fact, it has been shown that gradient boosting and other machine learning models perform better than traditional linear models in complex phenotypes when non-additive effects might be involved [31].

It is noteworthy to mention that some risk prediction models were mostly influenced by other factors, like sex for testosterone and creatinine, as seen in Fig. 5, which was identified as the variable with the highest influence in these models with the other features playing only a minor role in the prediction. This is to be expected, since testosterone is a sex-specific hormone and creatinine levels vary depending on the individual’s size and muscle mass, which is usually higher in men [32]. The true vs. predicted value plot and the top features figures for all the biomarkers’ models can be found in the supplementary materials (Figs. S27–S54).

**Fig. 5:**
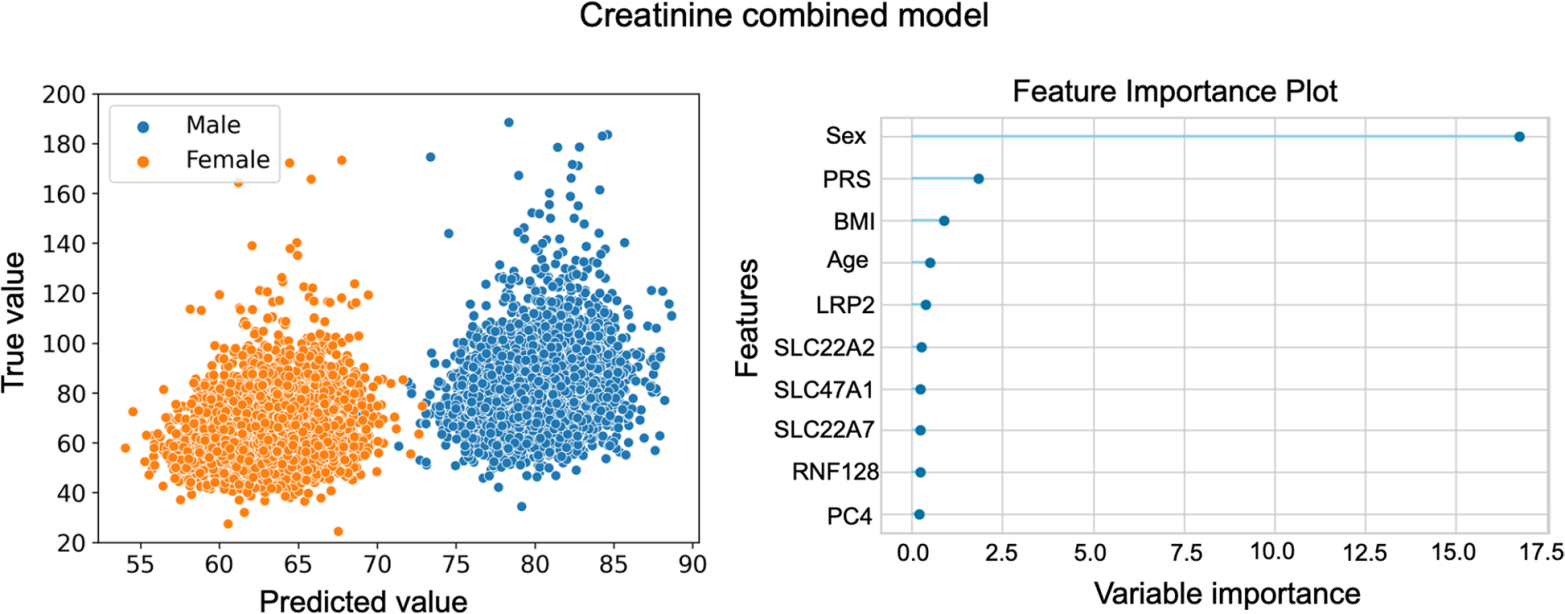
True vs. Predicted value plot (left) and top 10 features (right) for creatinine combined model. Values that are a 3 standard deviations away from the mean were eliminated for a better visualization

## Conclusion

In this study, we investigate the contribution of rare functional variants in blood biomarkers. We performed association analysis on gene-based burden scores and built genetic risk models using rare and common variant effects. The results suggest that gene-based score is a powerful instrument to identify gene-phenotype associations between rare-variants and complex phenotypes. While some of the associations were replicated by other methods, our tool has the advantage of producing individual-level scores that can be used for multiple subsequent analyses. Although gene-based scores have proven to be useful on the individual-level, traditional PRS provides more information for risk prediction purposes on the population-level scale. It is important to mention that these results are limited to the effects of rare and common variants at gene-based level. Even though we included non-linear models in the analysis to potentially detect gene-gene interactions, they cannot capture effects that happen at variant level. Furthermore, other potential factors influencing the genetic susceptibility (i.e., epigenetics, gene-environment) are not considered in our current work.

## Methods

### Cohort and data processing

All analyses were performed on the UK biobank cohort, which is a large-scale population-based biomedical database that contains data for half a million participants. Data include questionnaires, biomarkers, imaging and genetic data. For our analysis, we used imputed genotype data, whole exome sequencing data, biometric data (age, sex, BMI) and all blood biomarker measurements except for rheumatoid factor and estradiol, which were excluded because of low sample size. The UK biobank field identifiers used can be found in supplementary material (Table S1). Variants were annotated with genes using NCBI’s gene and reference sequences [33], gnomad allele frequency and CADD v1.6 raw scores [34]. We filtered the cohort to include participants with white British ancestry that have whole exome sequencing data and genotype data, resulting in n=145,464 samples. For individuals using the cholesterol lowering statins as medication, cholesterol, LDL and apolipoprotein B levels were adjusted by using previously estimated factors of 0.684, 0.749, and 0.719, respectively [35]. For risk prediction modeling, the cohort was split into three subsets: 60% (n=87,278) for constructing the PRS and feature selection, 30% (n=43,639) for training the prediction models, and 10% (n=14,547) for model testing. The number of samples per phenotype varied depending on the availability of measurements. Distribution and number of samples per biomarker can be found in the supplementary material (Fig. S1).

### Polygenic risk score (PRS)

To generate the PRS for each biomarker, we applied snpnet pipeline [36] on the the imputed genotyping samples of the construction dataset. This pipeline uses batch screening iterative lasso framework to select effect variants and generate polygenic score which can be used to calculate PRS for a cohort. We used the default parameters defined in snpnet pipeline for polgenic score derivation and excluded SNPs with MAF < 0.01. After polygenic score construction, we calculated the PRS for the remaining cohort to be included in the prediction model training and testing subsets.

### Rare variants analysis

We used GenRisk, a python package that implements a gene-based scoring system, association analysis, risk scores calculations and machine learning models generation [18]. The gene-based scoring system depends on frequency and functional annotations, with up-weighting function for rare variants. Gene-based scores (GBS) were derived from whole exome data for all individuals in the cohort, using default settings (MAF threshold < 0.01, beta weighting function with parameters 1 and 25), and associations were assessed for the 28 biomarkers with quantitative values. For association analysis, linear regression was applied to the gene-based scores of the whole cohort with BMI, age, sex and the first four genetic principal components (PCs) as covariates. The number of PCs was chosen based on the variance explained in UK biobank European cohort [37]. Manhattan and QQ plots were generated to visualize the results, and the lambda statistic, representing the inflation of *P*-values in comparison to the expected distribution of P, was also calculated. To account for multiple testing, Bonferroni correction was applied to adjust the *P*-values. Thus, the genome-wide significance threshold level was calculated based on the number of tested genes (0.05/18556 =2.69E-07).

### Feature selection

To reduce the numbers of input variables in prediction models, feature selection was applied on the GBS matrix to select genes that are associated with the respective biomarker. Association analysis was performed using linear regression with the same previously stated covariates on the GBS of the construction subset for each of the biomarker and genes with *P*-value < 0.05 after Bonferroni correction were selected as gene predictors. Number of gene predictors per biomarker can be found in Table 2.

### Risk prediction modeling

For each biomarker, four different prediction models were generated using the machine learning model training subset.

- Covariates model: biomarker = sex + age + BMI + PC1 + PC2 + PC3 + PC4
- GBS model: biomarker = covariates + GBS
- PRS model: biomarker = covariates + PRS
- Combined model: biomarker = covariates + GBS + PRS

Our tool, GenRisk, uses PyCaret as underlying framework for prediction model generation. PyCaret is a python library that implements different machine learning models and can be used for training and testing, selecting, fine tuning and finalizing models^1^. Different models (n=17) including linear, such as ridge, elastic net and lasso regression, and non-linear models, like gradient boosting and random forest regression, are tested. A list of all models can be found in the GenRisk documentation^2^. For the GBS, only the gene predictors that were selected in the feature selection step for each biomarker were included. All features were normalized by calculating the z-score. The training step was performed on the training set, with the corresponding biomarker as target, using 10 fold cross-validation and the best performing model for each biomarker is then finalized considering the complete training cohort and applied on the independent test set.

### Supplementary Information

**Additional file 1.**

**Additional file 2.**

## Data Availability

UK Biobank is a large-scale biomedical database and research resource. Data from UK Biobank (Genotyping data, exome data, and phenotypic data) are available upon application (http://www.ukbiobank.ac.uk/about-biobank-uk/). Restrictions apply to the availability of these data, which were used under license for the current study (Project ID: 81202).

## Abbreviations

AST: Aspartate aminotransferase
GBS: Gene-based scores
GWAS: Genome-wide association studies
LDL: Low-density lipoprotein
MAF: Minor allele frequency
PRS: Polygenic risk scores
SHBG: Sex hormone binding globulin
SNPs: Single nucleotide polymorphisms
SKAT: Sequence kernel association test

## Footnotes

1 https://pycaret.gitbook.io/docs/

2 https://genrisk.readthedocs.io/en/latest/
