# Supplementary material for: Gene-based burden scores identify rare variant associations for 28 blood biomarkers
Source: BMC Genom Data. 2023 Sep 4;24:50. doi: 10.1186/s12863-023-01155-0 (PMC10476296; doi:10.1186/s12863-023-01155-0)
Supplement: Supplementary file 1 — Additional file 1. [file 12863_2023_1155_MOESM1_ESM.pdf]

Supplementary material

Table of figures

Figure S1 Distribution of samples across biomarkers. 3
Figure S2 Association analysis summary for albumin. 4
Figure S3 Association analysis summary for alkaline phosphatase 5
Figure S4 Association analysis summary for apolipoprotein A 6
*Figure S5 Association analysis summary for apolipoprotein B** 7
Figure S6 Association analysis summary for C-reactive protein 8
Figure S7 Association analysis summary for calcium 9
*Figure S8 Association analysis summary for cholesterol** 10
Figure S9 Association analysis summary for creatinine 11
Figure S10 Association analysis summary for cystatin C 12
Figure S11 Association analysis summary for direct bilirubin. 13
Figure S12 Association analysis summary for gamma glutamyltransferase 14
Figure S13 Association analysis summary for glucose 15
Figure S14 Association analysis summary for glycated haemoglobin (HbA1c) 16
Figure S15 Association analysis summary for HDL cholesterol. 17
Figure S16 Association analysis summary for IGF1. 18
Figure S17 Association analysis summary for lipoprotein A 19
Figure S18 Association analysis summary for phosphate 20
Figure S19 Association analysis summary for SHBG 21
Figure S20 Association analysis summary for testosterone 22
Figure S21 Association analysis summary for total bilirubin 23
Figure S22 Association analysis summary for total protein 24
Figure S23 Association analysis summary for triglycerides 25
Figure S24 Association analysis summary for urate. 27
Figure S25 Association analysis summary for urea 28
Figure S26 Association analysis summary for vitamin D 28
Figure S27 True vs. Predicted value plot (left) and top 10 features (right) for alanine aminotransferase. 29
Figure S28 True vs. Predicted value plot (left) and top 10 features (right) for albumin 30
Figure S29 True vs. Predicted value plot (left) and top 10 features (right) for alkaline phosphatase. 31
Figure S30 True vs. Predicted value plot (left) and top 10 features (right) for apolipoprotein A 32
*Figure S31 True vs. Predicted value plot (left) and top 10 features (right) for apolipoprotein B** 33
Figure S32 True vs. Predicted value plot (left) and top 10 features (right) for aspartate aminotransferasel. 34
Figure S33 True vs. Predicted value plot (left) and top 10 features (right) for calcium 35
*Figure S 34 True vs. Predicted value plot (left) and top 10 features (right) for cholesterol** 36
Figure S35 True vs. Predicted value plot (left) and top 10 features (right) for C reactive protein 37
Figure S36 True vs. Predicted value plot (left) and top 10 features (right) for creatinine 38
Figure S37 True vs. Predicted value plot (left) and top 10 features (right) for cystatin C. 39
Figure S38 True vs. Predicted value plot (left) and top 10 features (right) for direct bilirubin 40
Figure S39 True vs. Predicted value plot (left) and top 10 features (right) for gamma glutamyltransferase 41
Figure S40 True vs. Predicted value plot (left) and top 10 features (right) for glucose 42
Figure S41 True vs. Predicted value plot (left) and top 10 features (right) for glycated haemoglobin (HbA1c) 43
Figure S42 True vs. Predicted value plot (left) and top 10 features (right) for HDL cholesterol. 44
Figure S43 True vs. Predicted value plot (left) and top 10 features (right) for IGF1 45
*Figure S44 True vs. Predicted value plot (left) and top 10 features (right) for LDL direct** 46
Figure S45 True vs. Predicted value plot (left) and top 10 features (right) for lipoprotein A 47
Figure S46 True vs. Predicted value plot (left) and top 10 features (right) for phosphate. 48
Figure S47 True vs. Predicted value plot (left) and top 10 features (right) for SHBG 49
Figure S48 True vs. Predicted value plot (left) and top 10 features (right) for testosterone. 50
Figure S49 True vs. Predicted value plot (left) and top 10 features (right) for total bilirubin 51
Figure S50 True vs. Predicted value plot (left) and top 10 features (right) for total protein. 52
Figure S51 True vs. Predicted value plot (left) and top 10 features (right) for triglycerides. 53

Figure S52 True vs. Predicted value plot (left) and top 10 features (right) for urate..54
Figure S53 True vs. Predicted value plot (left) and top 10 features (right) for urea...55
Figure S54 True vs. Predicted value plot (left) and top 10 features (right) for vitamin D.....................................56

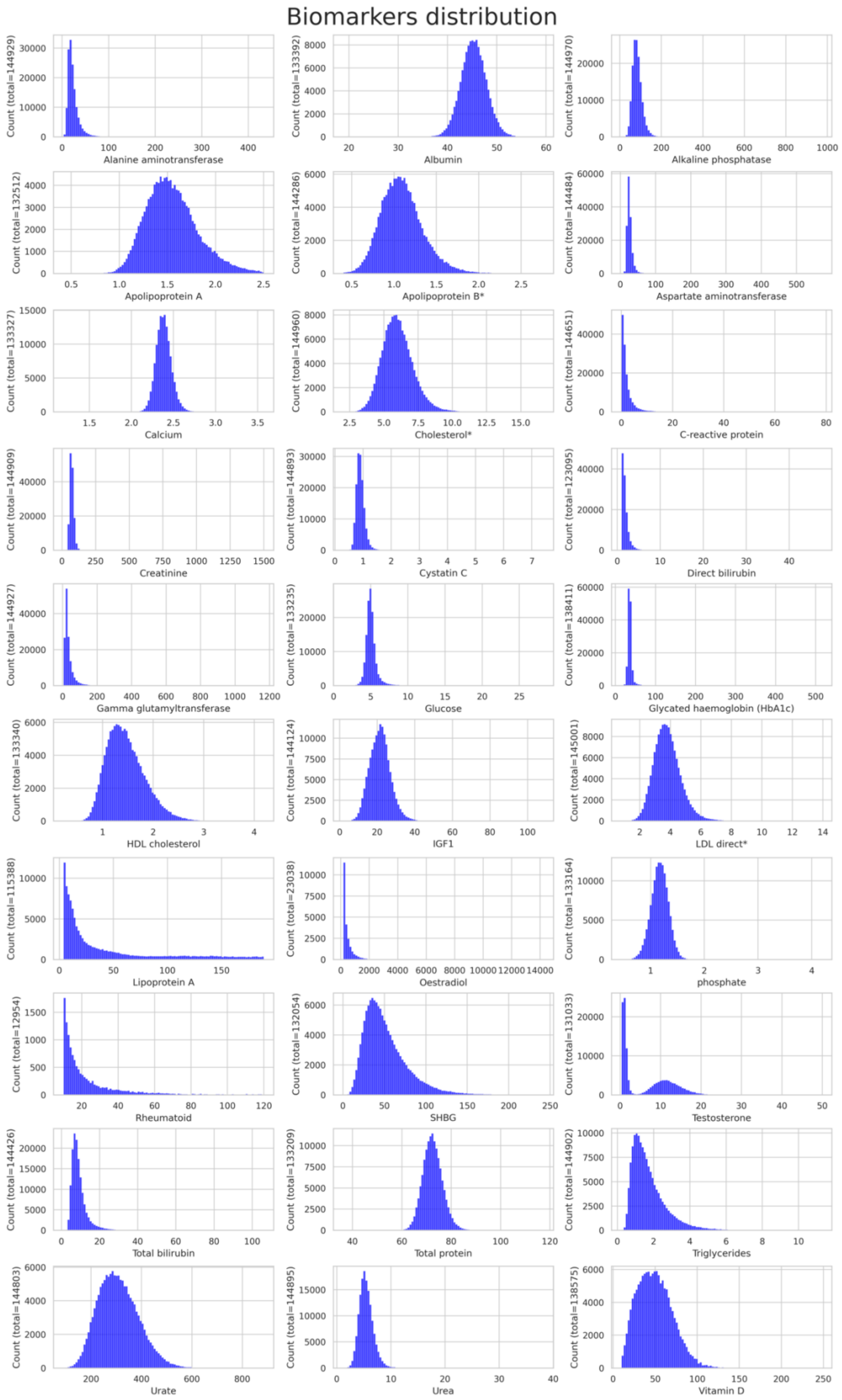

Figure S1 Distribution of samples across biomarkers.

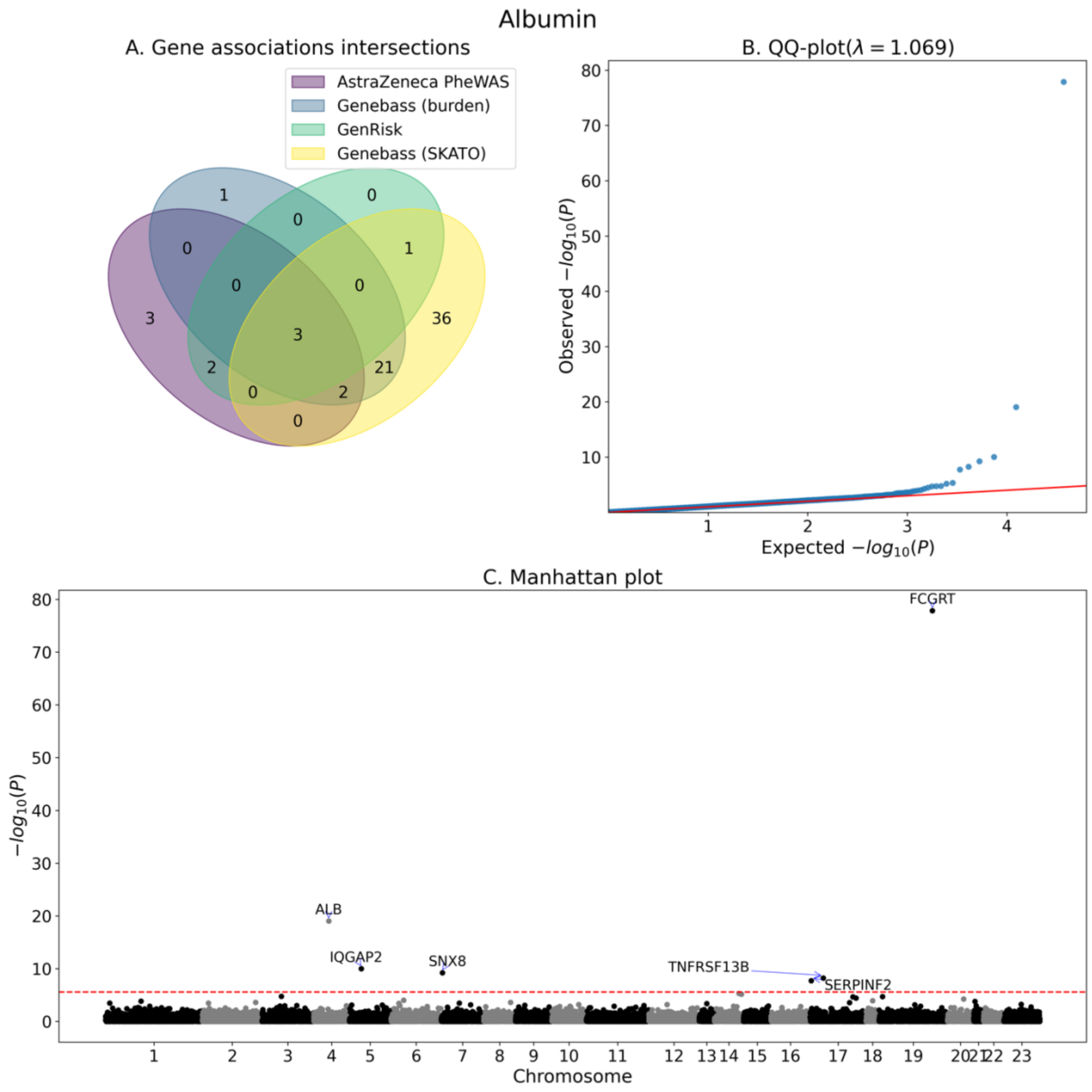

Figure S2 Association analysis summary for albumin.

A. Venn diagram of the number significantly associated genes as identified by GenRisk, AstraZenica PheWAS and genebass. B. QQ-plot of the P-values of GenRisk pipeline results. C. Manhattan plot of GenRisk pipeline results.

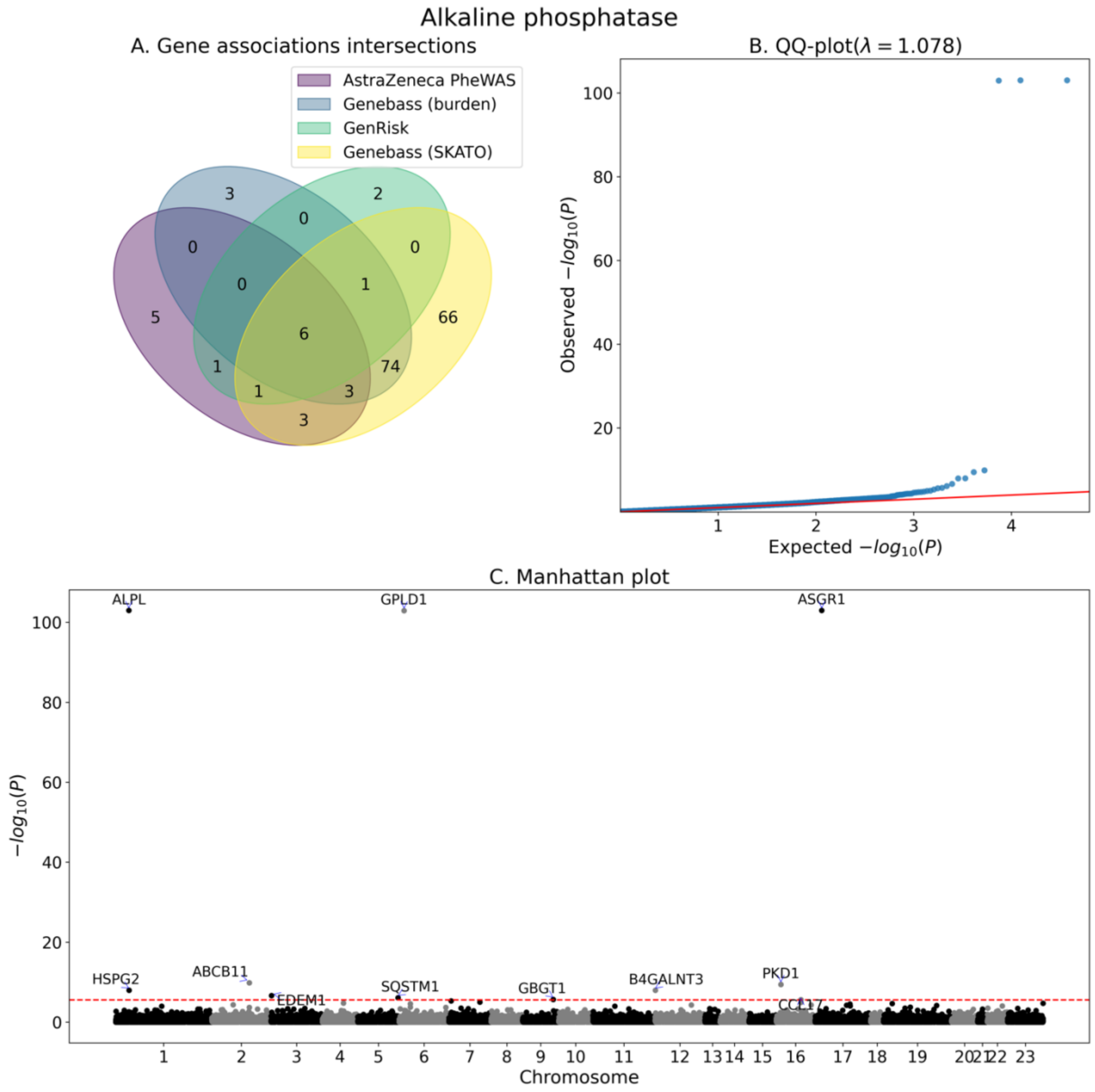

Figure S3 Association analysis summary for alkaline phosphatase.

A. Venn diagram of the number significantly associated genes as identified by GenRisk, AstraZenica PheWAS and genebass. B. QQ-plot of the P-values of GenRisk pipeline results. C. Manhattan plot of GenRisk pipeline results.

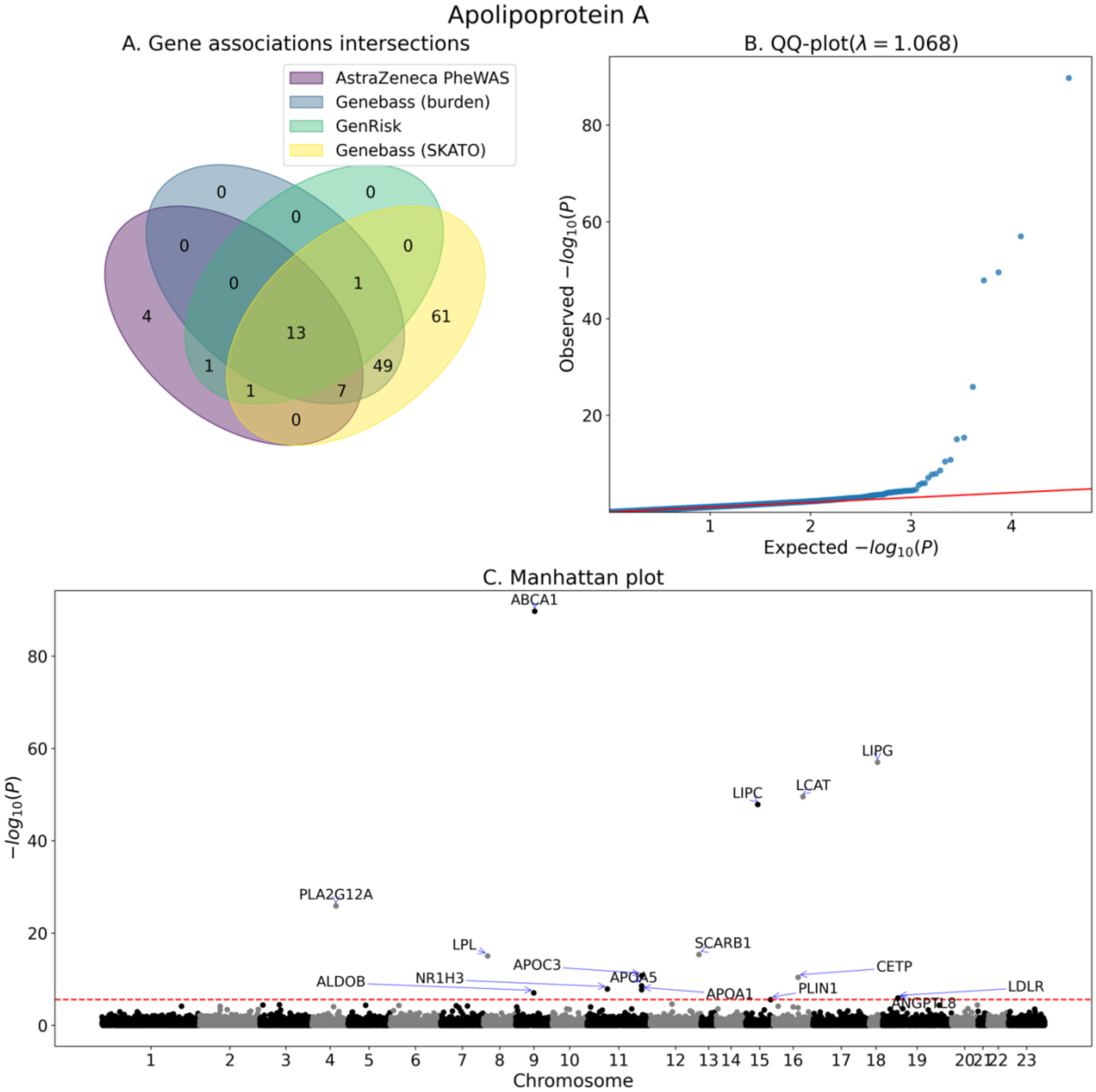

Figure S4 Association analysis summary for apolipoprotein A.

A. Venn diagram of the number significantly associated genes as identified by GenRisk, AstraZenica PheWAS and genebass. B. QQ-plot of the P-values of GenRisk pipeline results. C. Manhattan plot of GenRisk pipeline results.

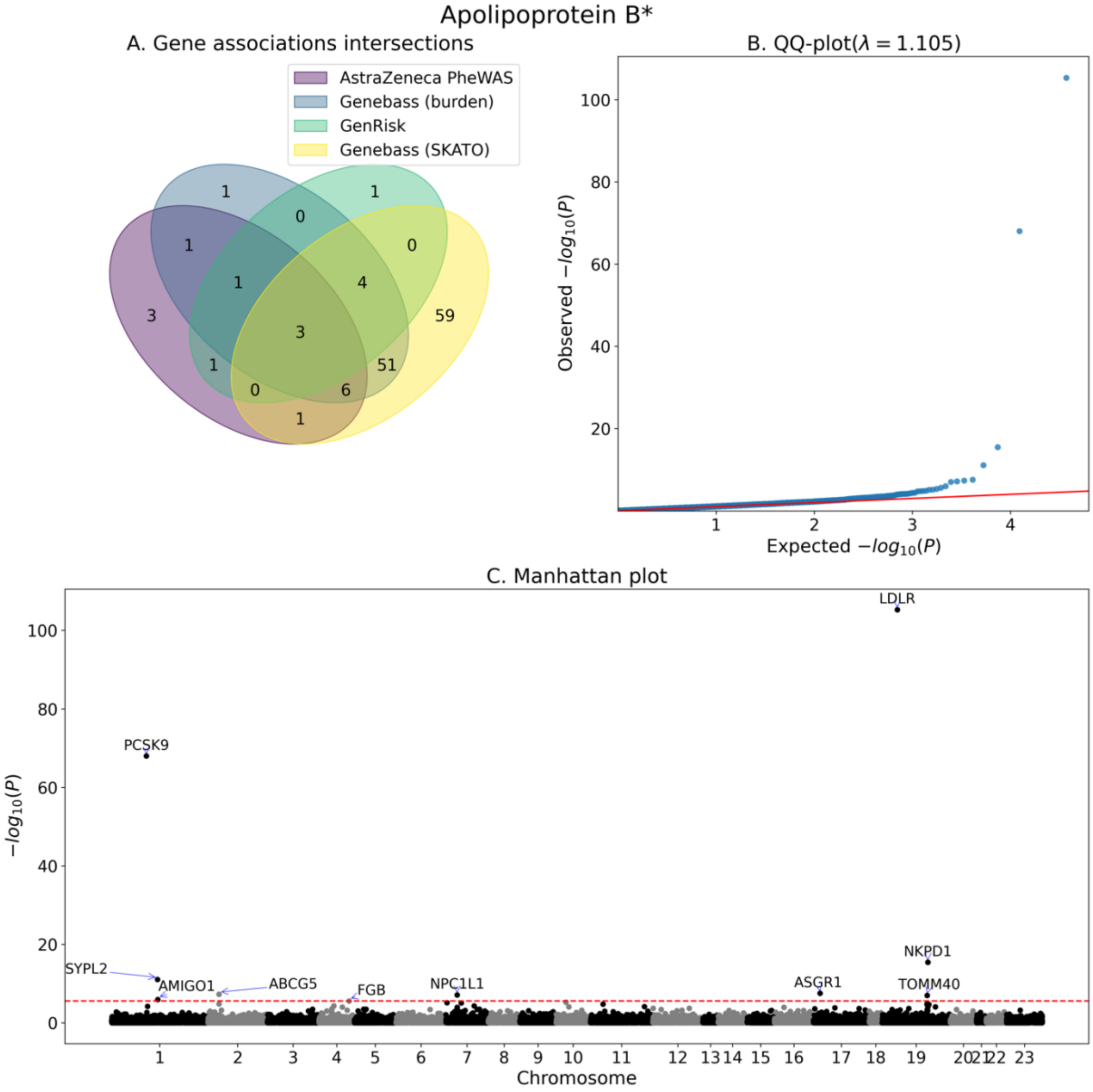

Figure S5 Association analysis summary for apolipoprotein B*.
A. Venn diagram of the number significantly associated genes as identified by GenRisk, AstraZenica PheWAS and genebass. B. QQ-plot of the P-values of GenRisk pipeline results. C. Manhattan plot of GenRisk pipeline results.
* statin adjusted values

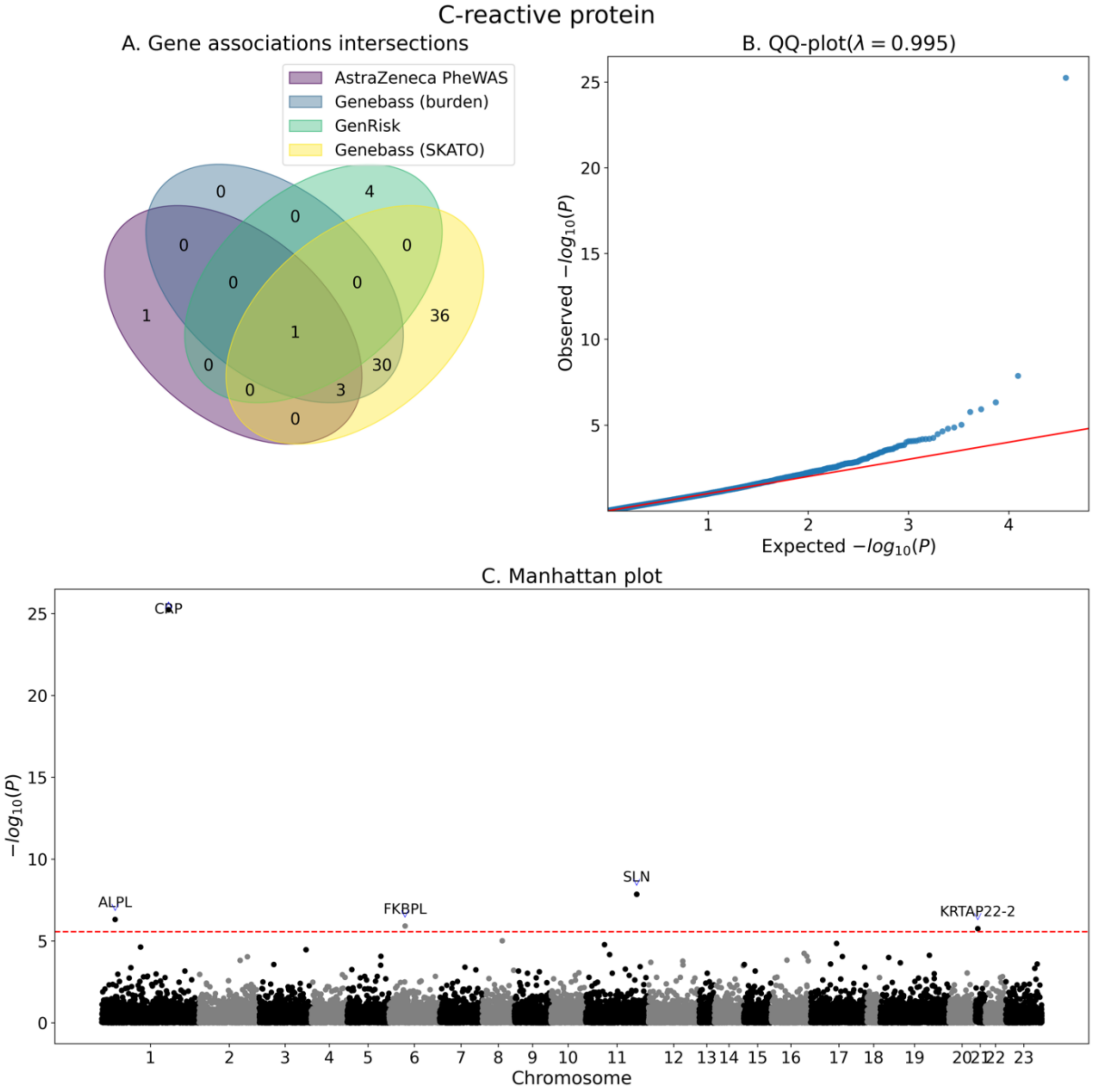

Figure S6 Association analysis summary for C-reactive protein.

A. Venn diagram of the number significantly associated genes as identified by GenRisk, AstraZenica PheWAS and genebass. B. QQ-plot of the P-values of GenRisk pipeline results. C. Manhattan plot of GenRisk pipeline results.

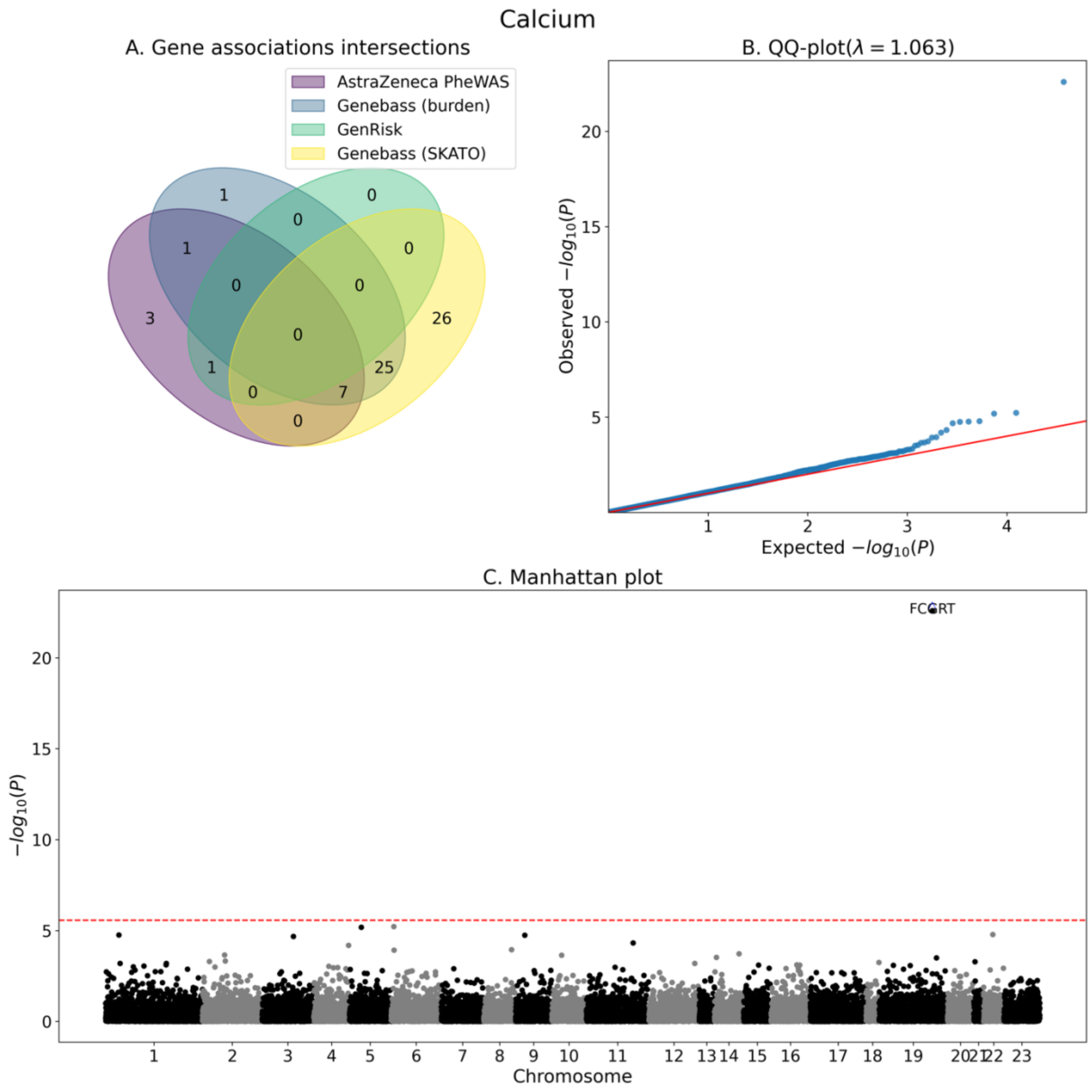

Figure S7 Association analysis summary for calcium.

A. Venn diagram of the number significantly associated genes as identified by GenRisk, AstraZenica PheWAS and genebass. B. QQ-plot of the P-values of GenRisk pipeline results. C. Manhattan plot of GenRisk pipeline results.

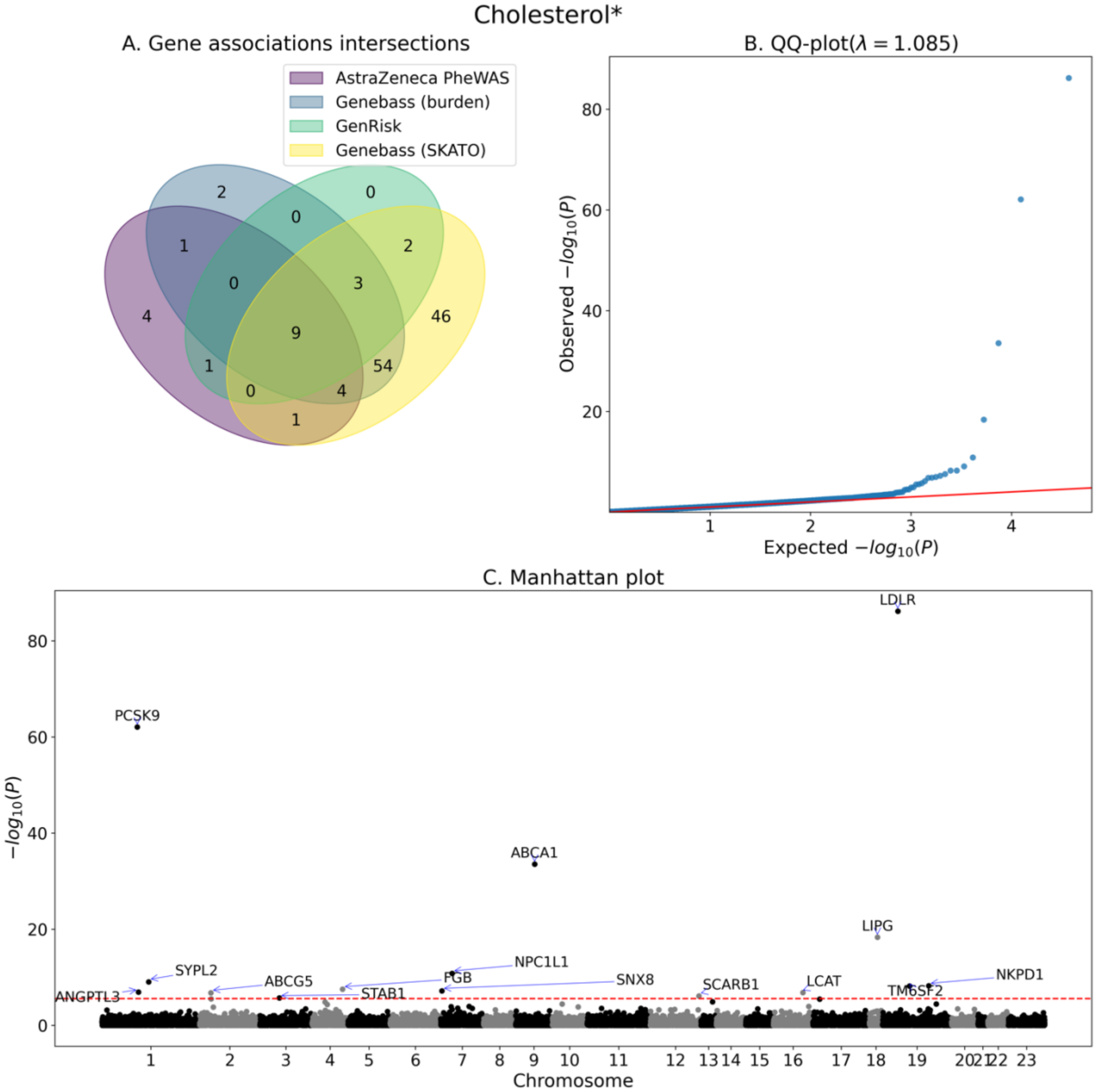

Figure S8 Association analysis summary for cholesterol*.

A. Venn diagram of the number significantly associated genes as identified by GenRisk, AstraZenica PheWAS and genebass. B. QQ-plot of the P-values of GenRisk pipeline results. C. Manhattan plot of GenRisk pipeline results.

* statin adjusted values

Creatinine

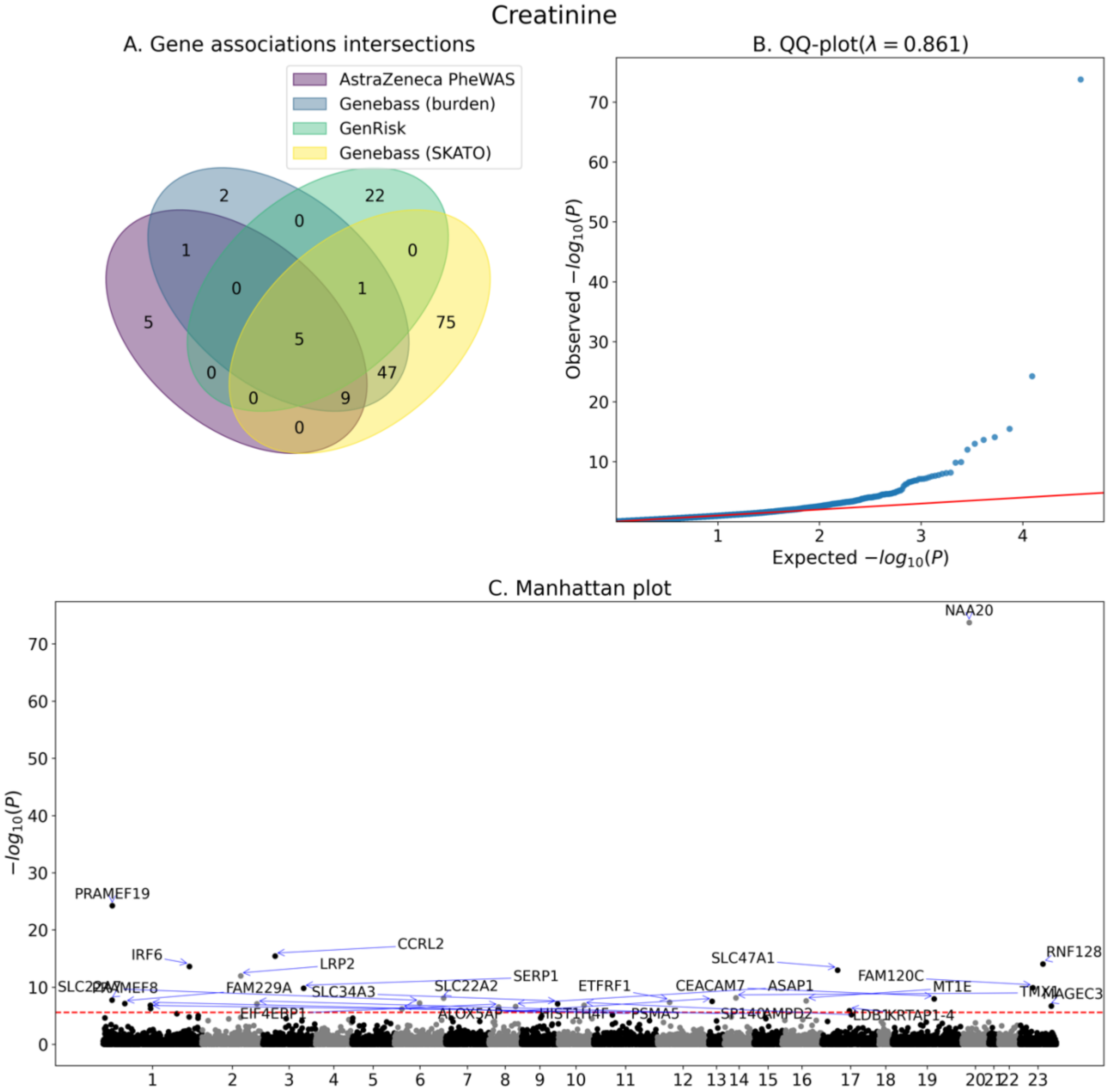

Figure S9 Association analysis summary for creatinine.

A. Venn diagram of the number significantly associated genes as identified by GenRisk, AstraZenica PheWAS and genebass. B. QQ-plot of the P-values of GenRisk pipeline results. C. Manhattan plot of GenRisk pipeline results.

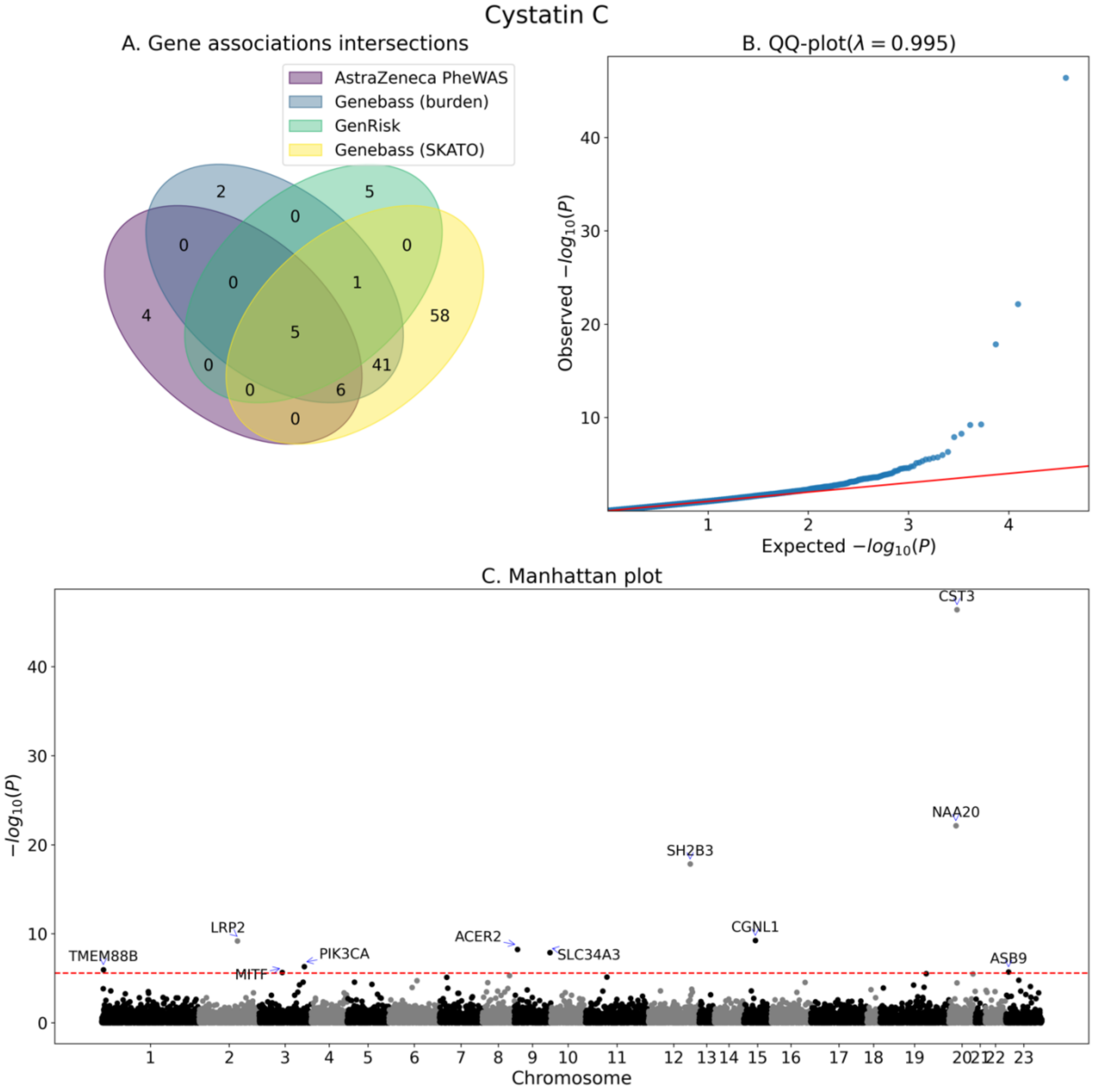

Figure S10 Association analysis summary for cystatin C.

A. Venn diagram of the number significantly associated genes as identified by GenRisk, AstraZenica PheWAS and genebass. B. QQ-plot of the P-values of GenRisk pipeline results. C. Manhattan plot of GenRisk pipeline results.

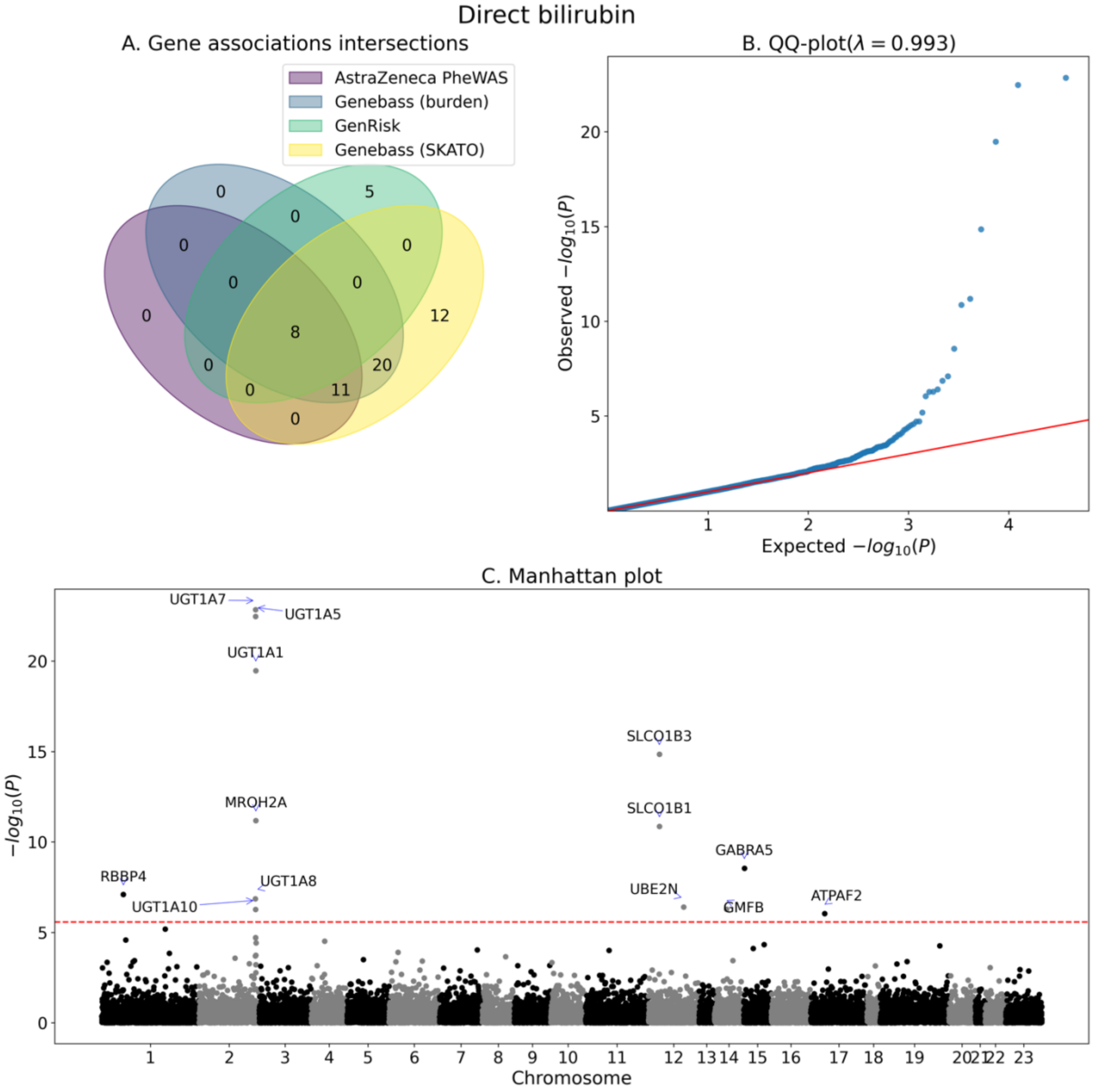

Figure S11 Association analysis summary for direct bilirubin.

A. Venn diagram of the number significantly associated genes as identified by GenRisk, AstraZenica PheWAS and genebass. B. QQ-plot of the P-values of GenRisk pipeline results. C. Manhattan plot of GenRisk pipeline results.

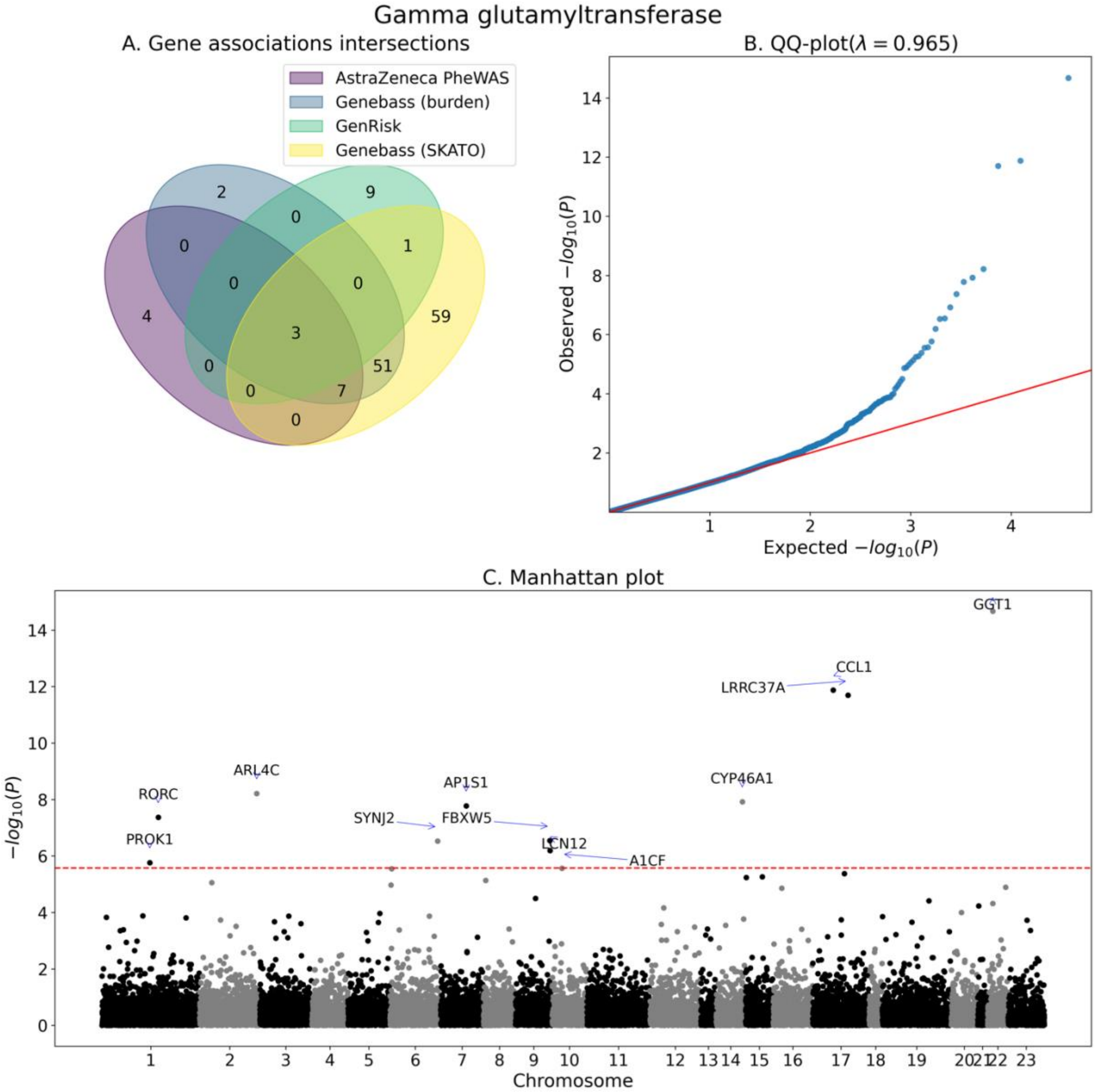

Figure S12 Association analysis summary for gamma glutamyltransferase.
A. Venn diagram of the number significantly associated genes as identified by GenRisk, AstraZenica PheWAS and genebass. B. QQ-plot of the P-values of GenRisk pipeline results. C. Manhattan plot of GenRisk pipeline results.

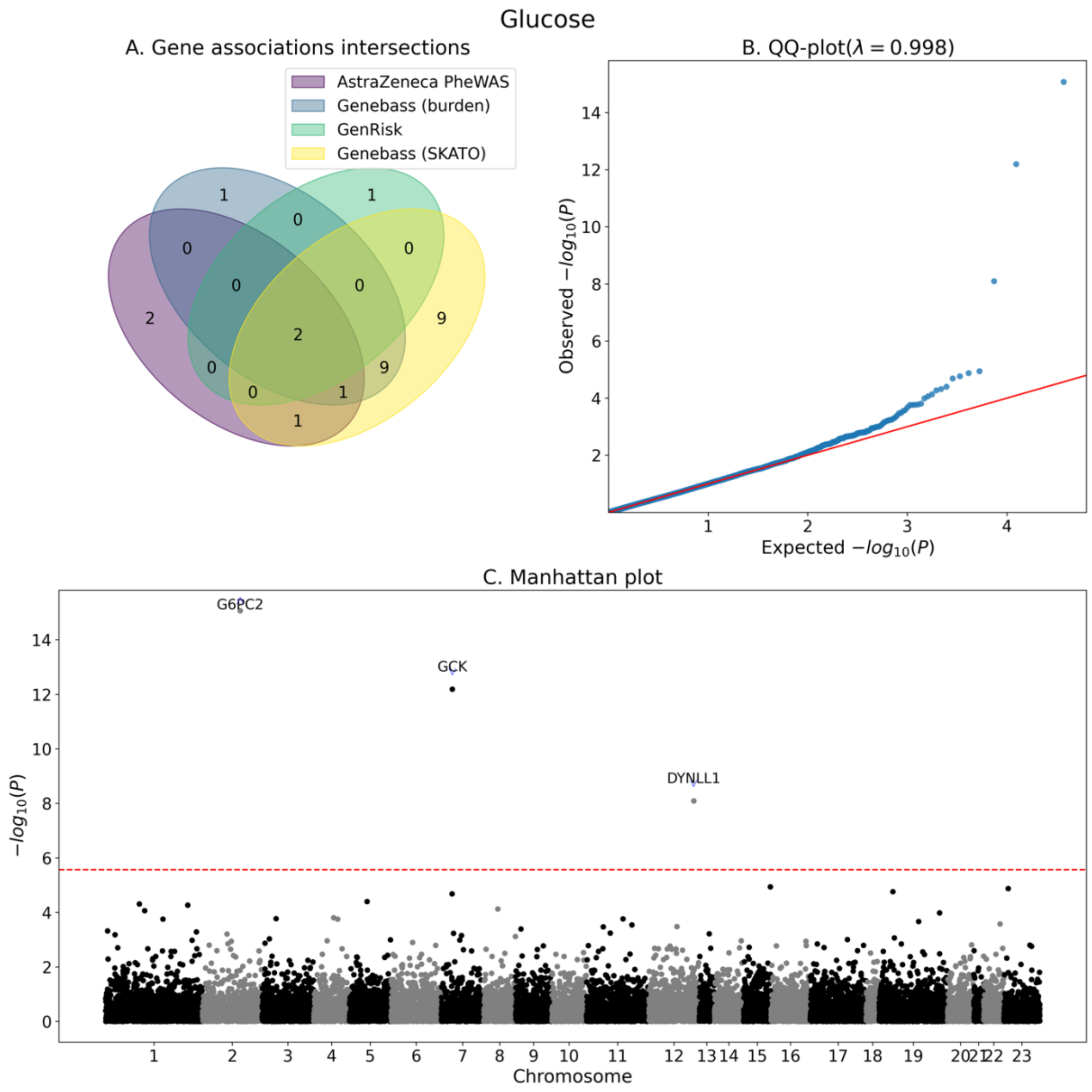

Figure S13 Association analysis summary for glucose.

A. Venn diagram of the number significantly associated genes as identified by GenRisk, AstraZenica PheWAS and genebass. B. QQ-plot of the P-values of GenRisk pipeline results. C. Manhattan plot of GenRisk pipeline results.

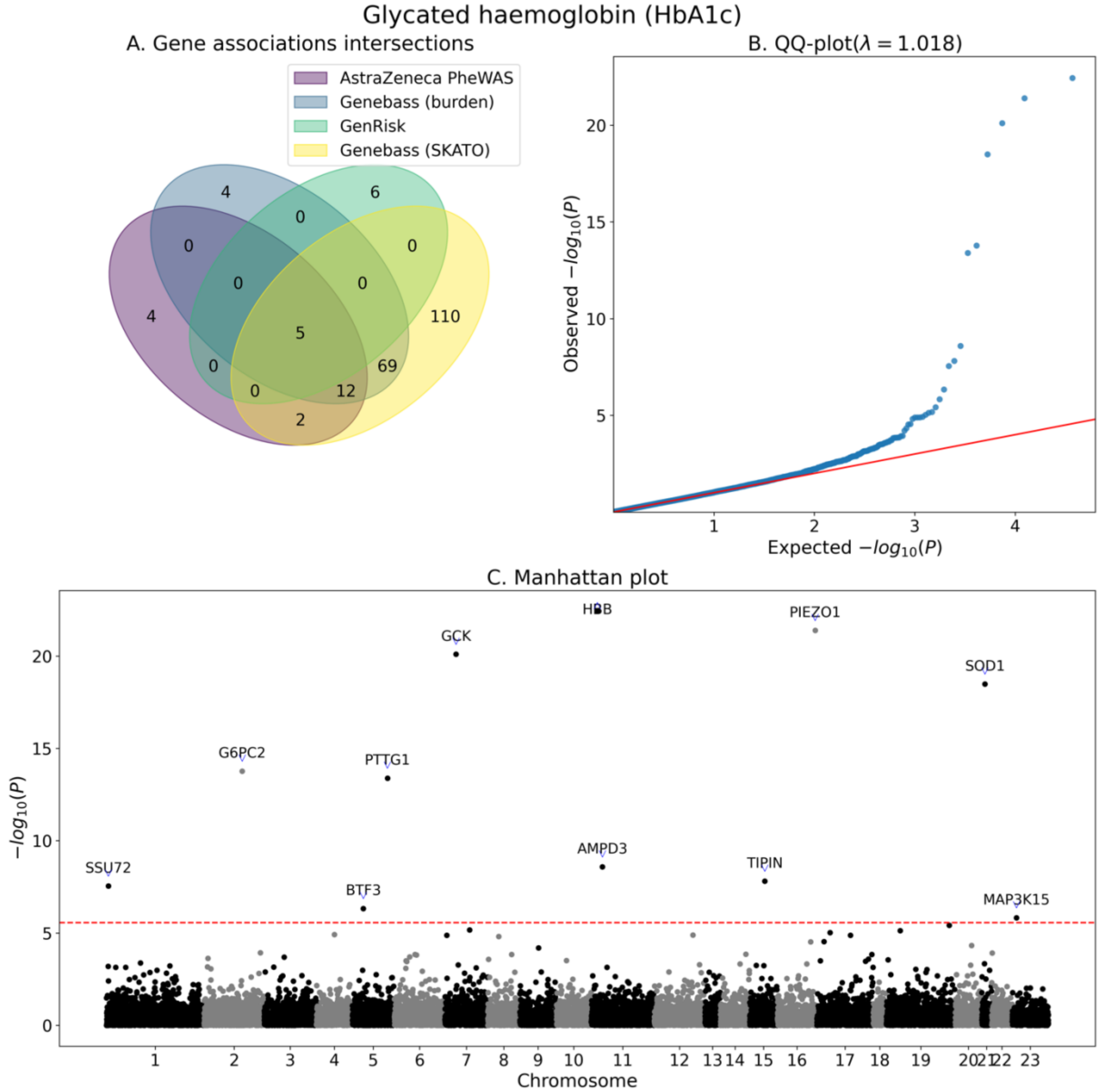

Figure S14 Association analysis summary for glycated haemoglobin (HbA1c).
A. Venn diagram of the number significantly associated genes as identified by GenRisk, AstraZenica PheWAS and genebass. B. QQ-plot of the P-values of GenRisk pipeline results. C. Manhattan plot of GenRisk pipeline results.

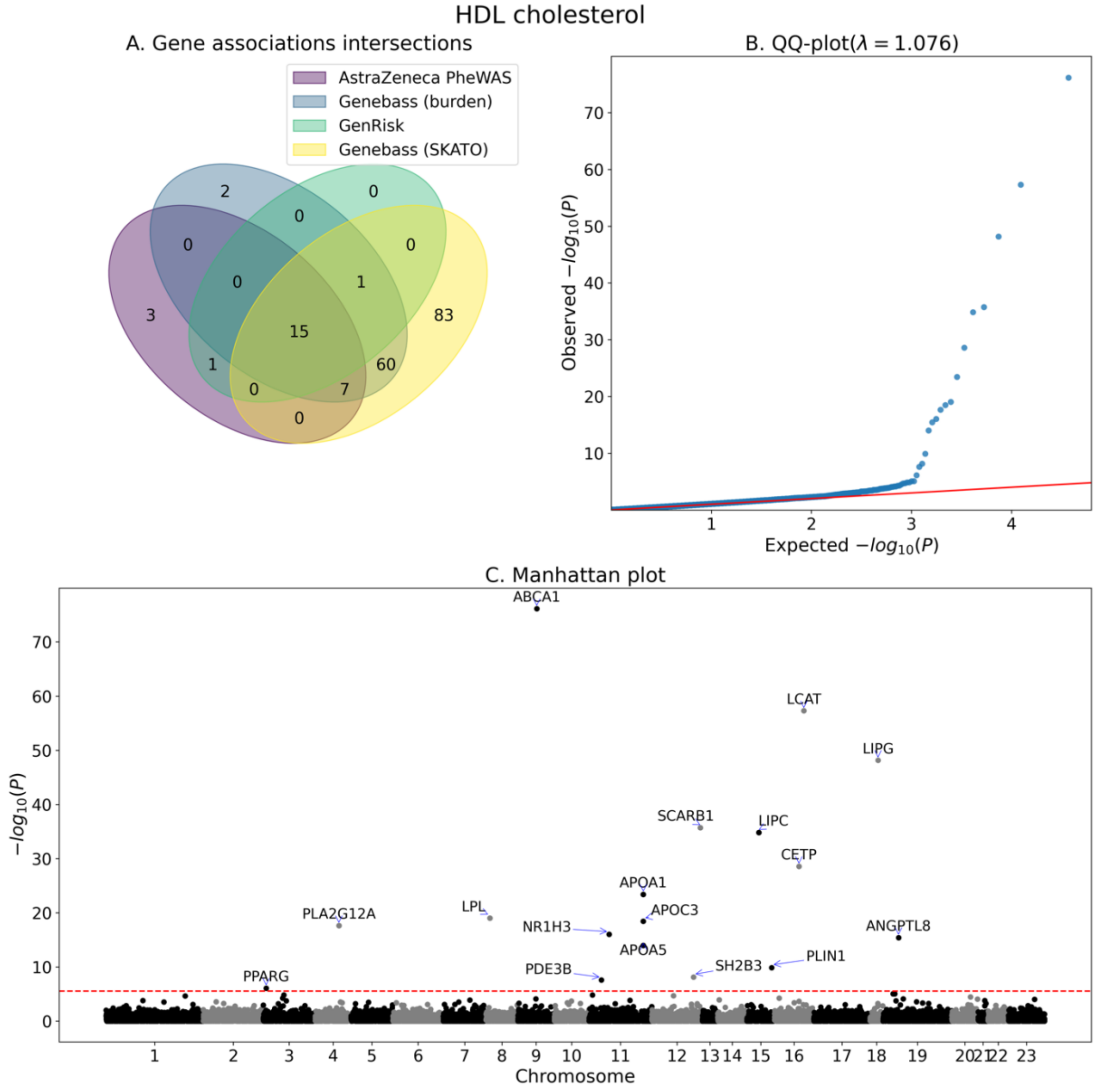

Figure S15 Association analysis summary for HDL cholesterol.
A. Venn diagram of the number significantly associated genes as identified by GenRisk, AstraZenica PheWAS and genebass. B. QQ-plot of the P-values of GenRisk pipeline results. C. Manhattan plot of GenRisk pipeline results.

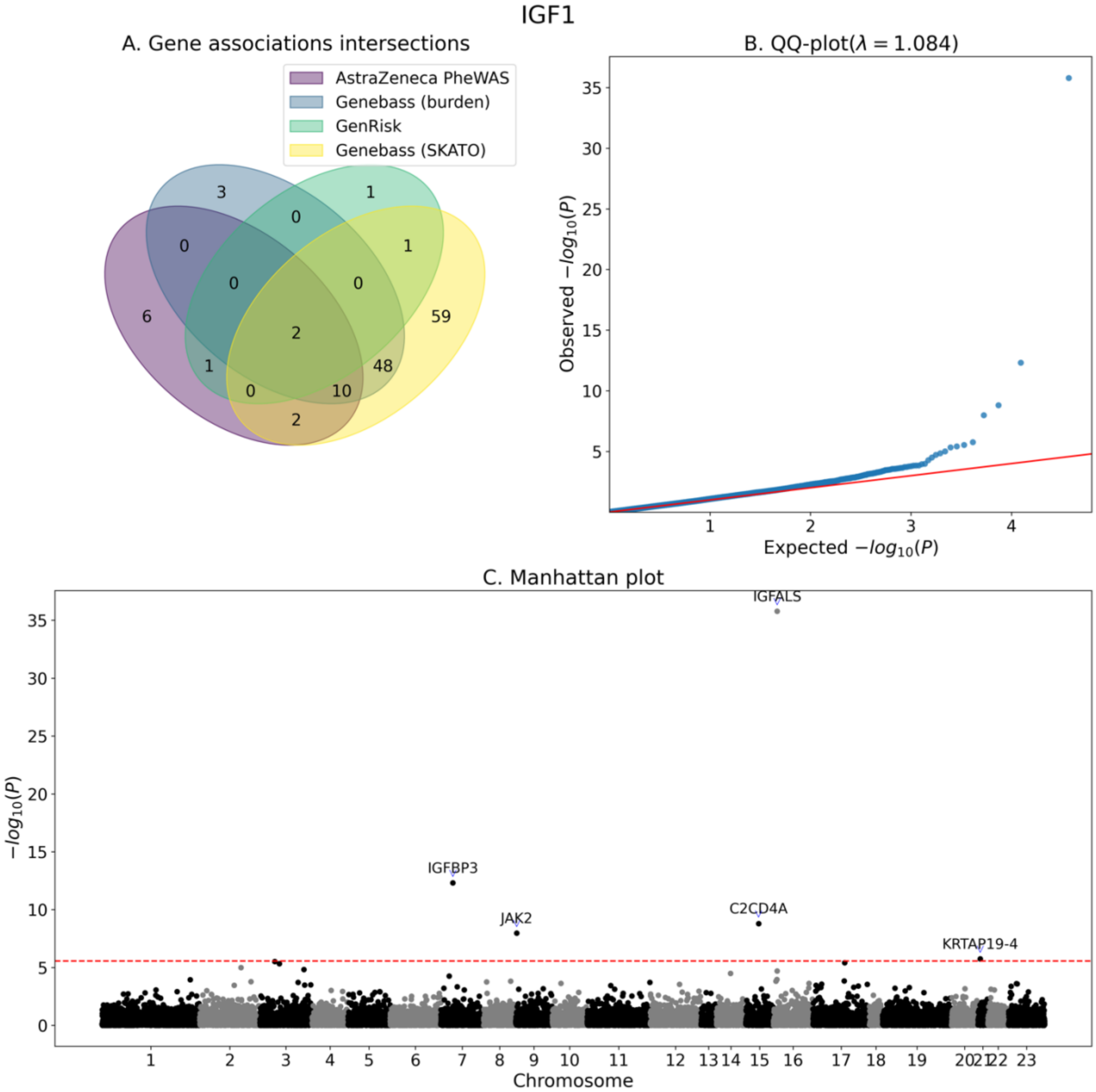

Figure S16 Association analysis summary for IGF1.

A. Venn diagram of the number significantly associated genes as identified by GenRisk, AstraZenica PheWAS and genebass. B. QQ-plot of the P-values of GenRisk pipeline results. C. Manhattan plot of GenRisk pipeline results.

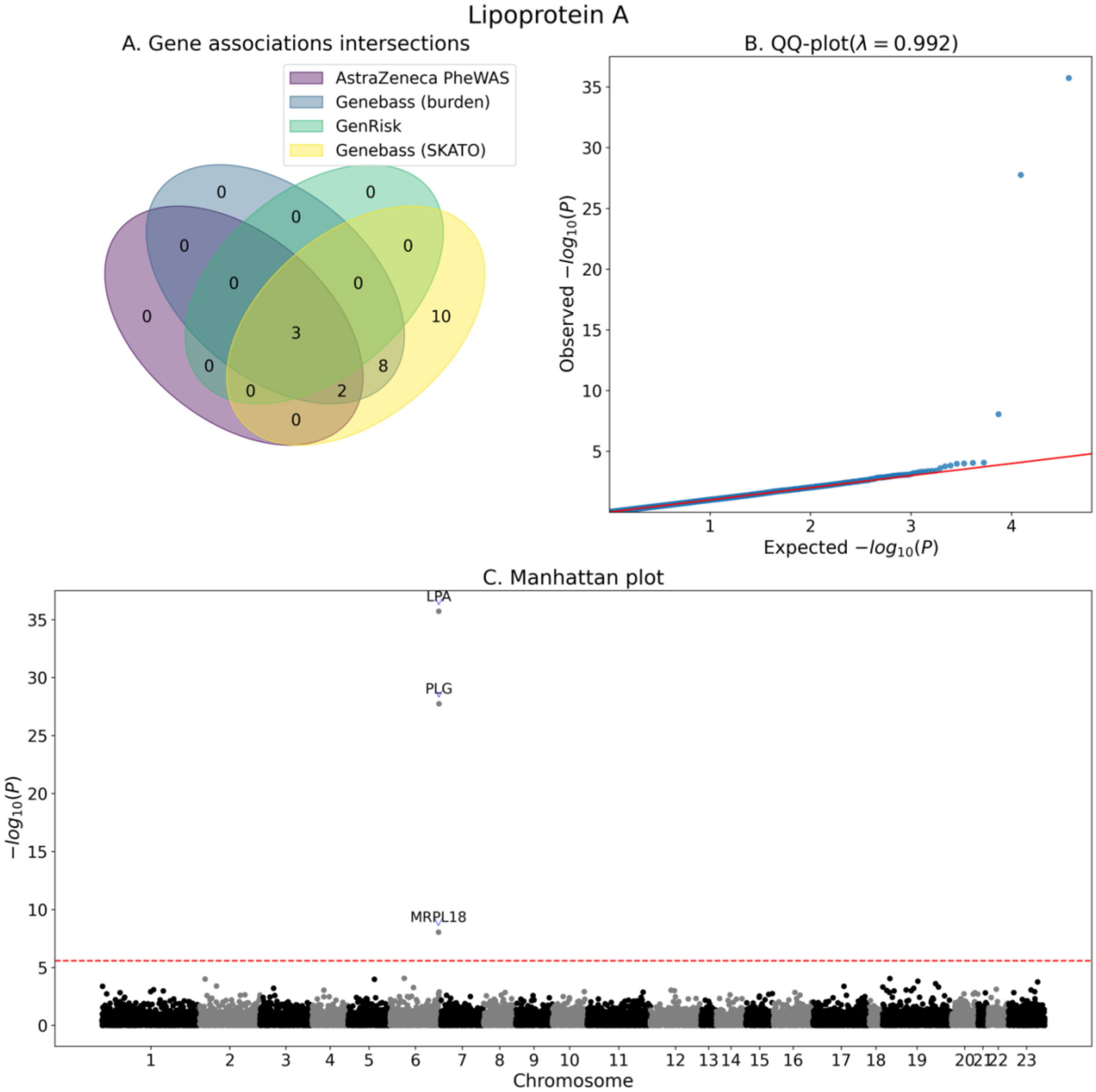

Figure S17 Association analysis summary for lipoprotein A.

A. Venn diagram of the number significantly associated genes as identified by GenRisk, AstraZenica PheWAS and genebass. B. QQ-plot of the P-values of GenRisk pipeline results. C. Manhattan plot of GenRisk pipeline results.

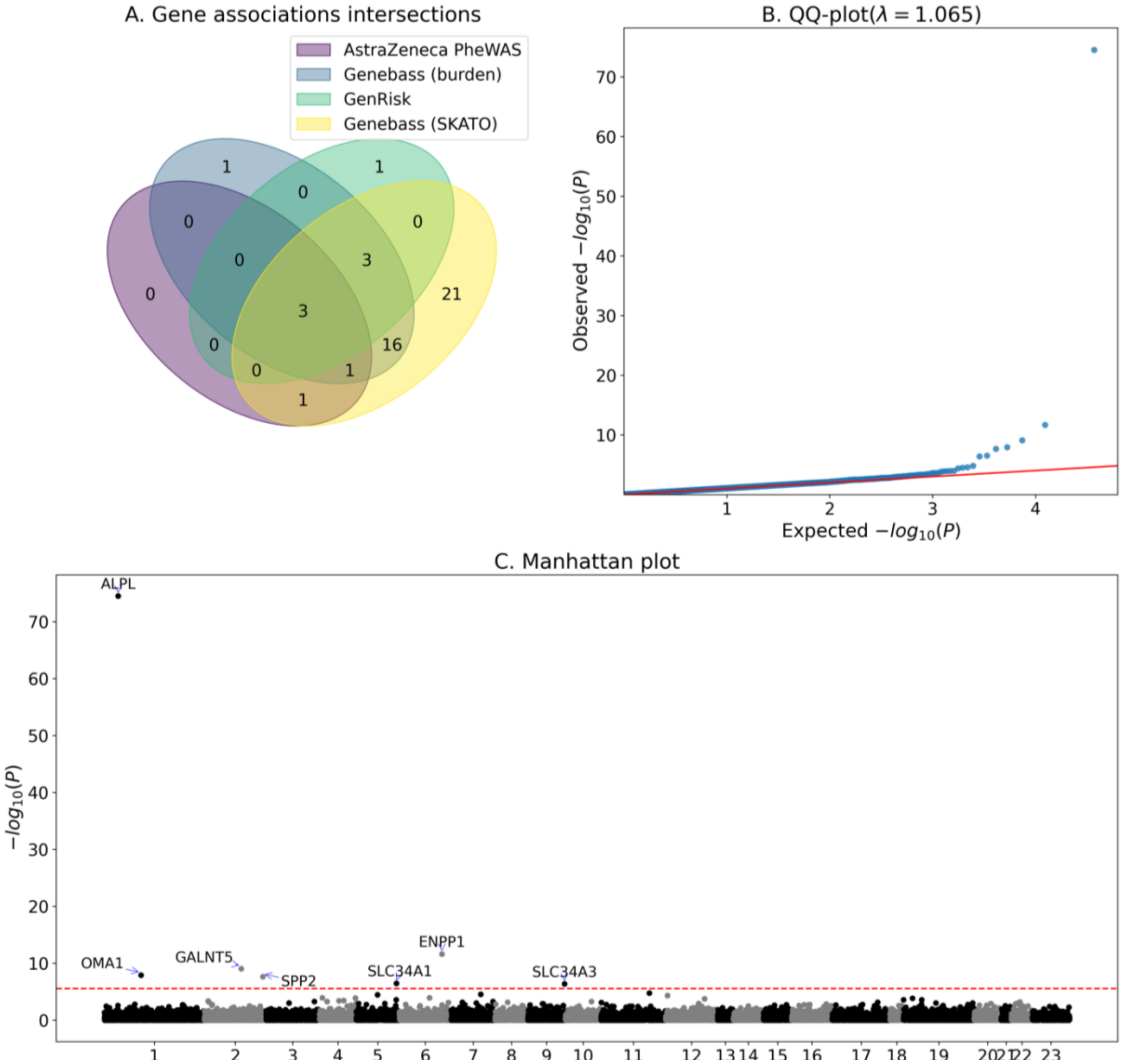

Figure S18 Association analysis summary for phosphate.

A. Venn diagram of the number significantly associated genes as identified by GenRisk, AstraZenica PheWAS and genebass. B. QQ-plot of the P-values of GenRisk pipeline results. C. Manhattan plot of GenRisk pipeline results.

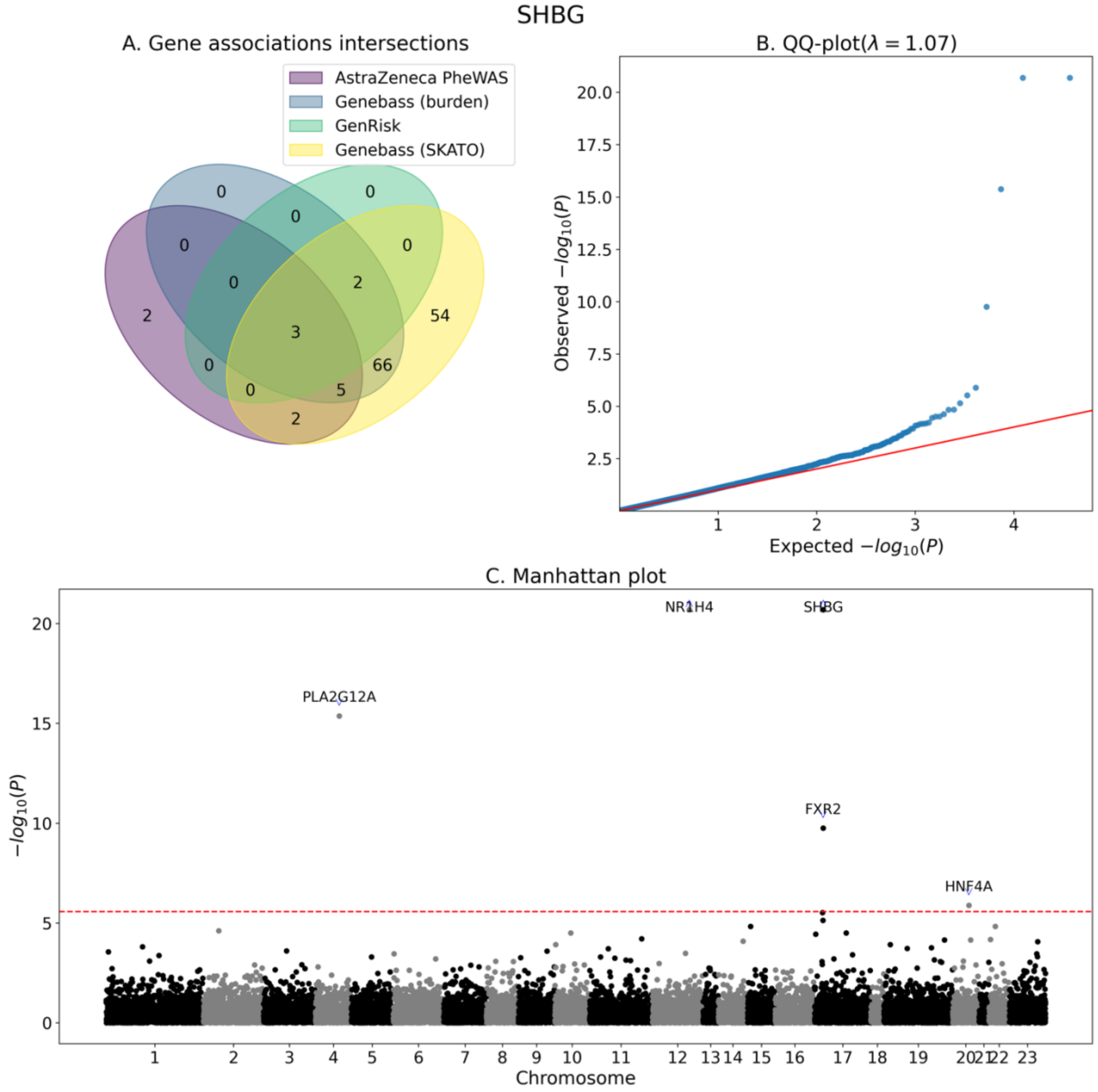

Figure S19 Association analysis summary for SHBG.
A. Venn diagram of the number significantly associated genes as identified by GenRisk, AstraZenica PheWAS and genebass. B. QQ-plot of the P-values of GenRisk pipeline results. C. Manhattan plot of GenRisk pipeline results.

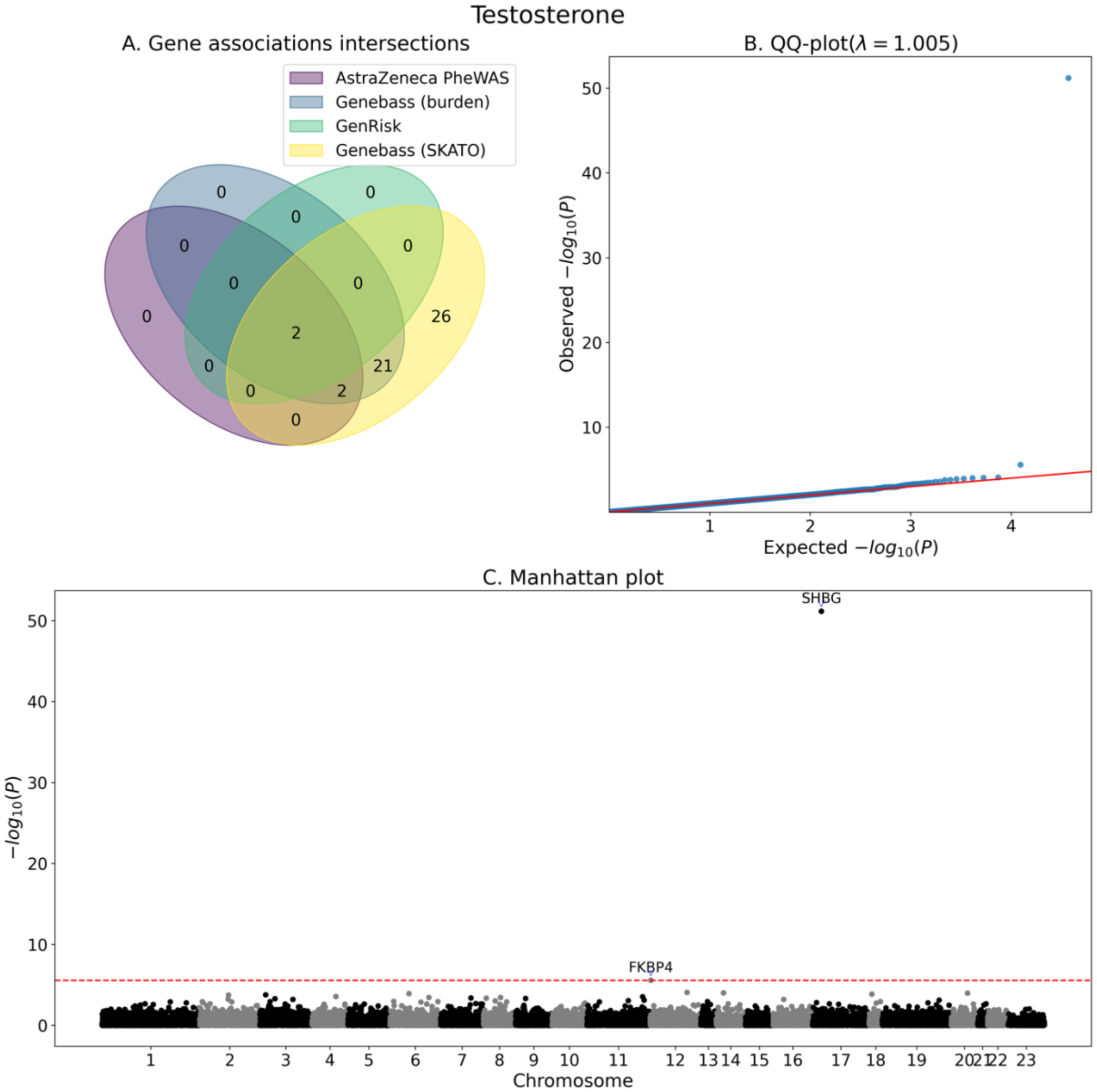

Figure S20 Association analysis summary for testosterone.

A. Venn diagram of the number significantly associated genes as identified by GenRisk, AstraZenica PheWAS and genebass. B. QQ-plot of the P-values of GenRisk pipeline results. C. Manhattan plot of GenRisk pipeline results.

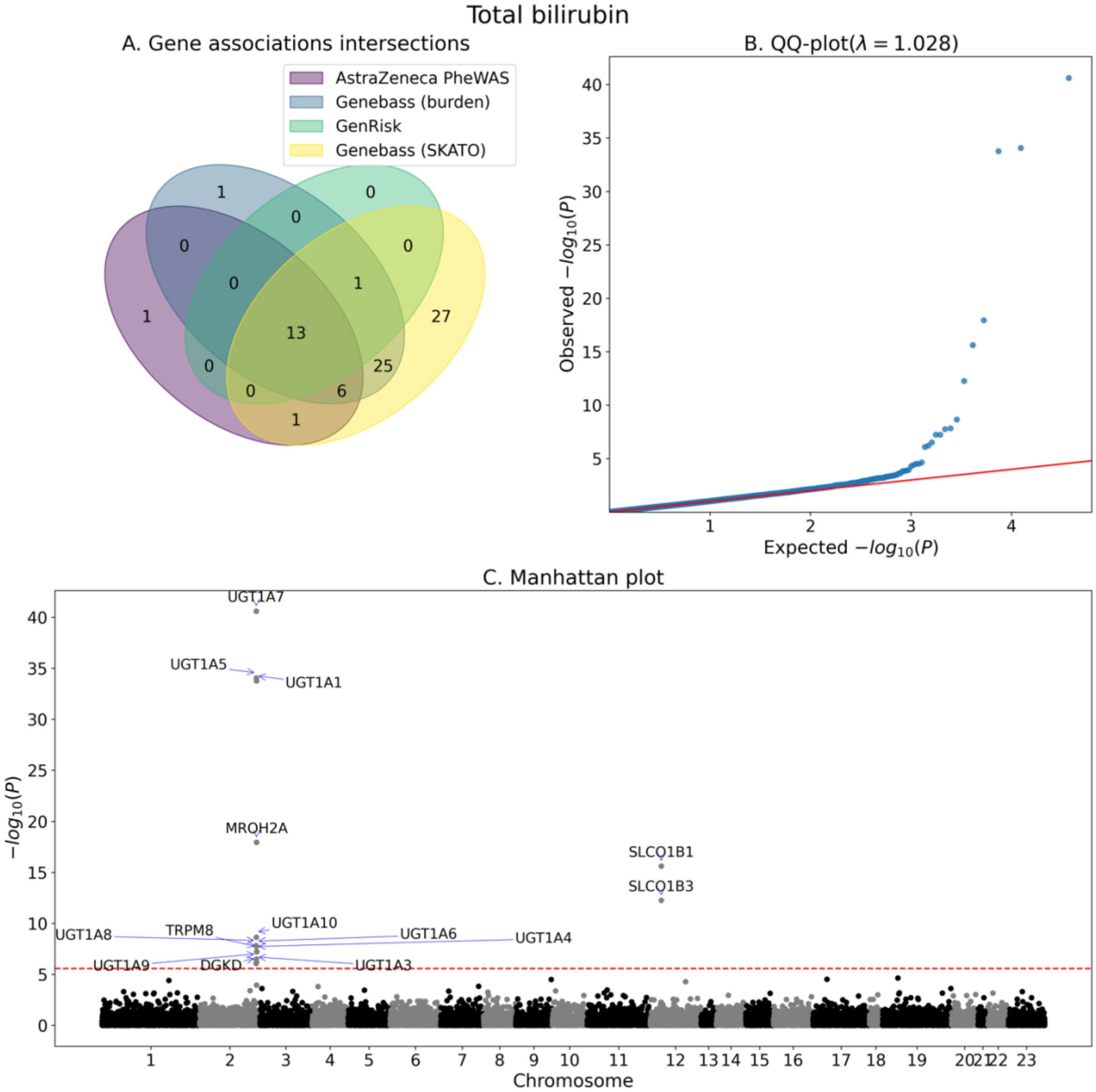

Figure S21 Association analysis summary for total bilirubin.

A. Venn diagram of the number significantly associated genes as identified by GenRisk, AstraZenica PheWAS and genebass. B. QQ-plot of the P-values of GenRisk pipeline results. C. Manhattan plot of GenRisk pipeline results.

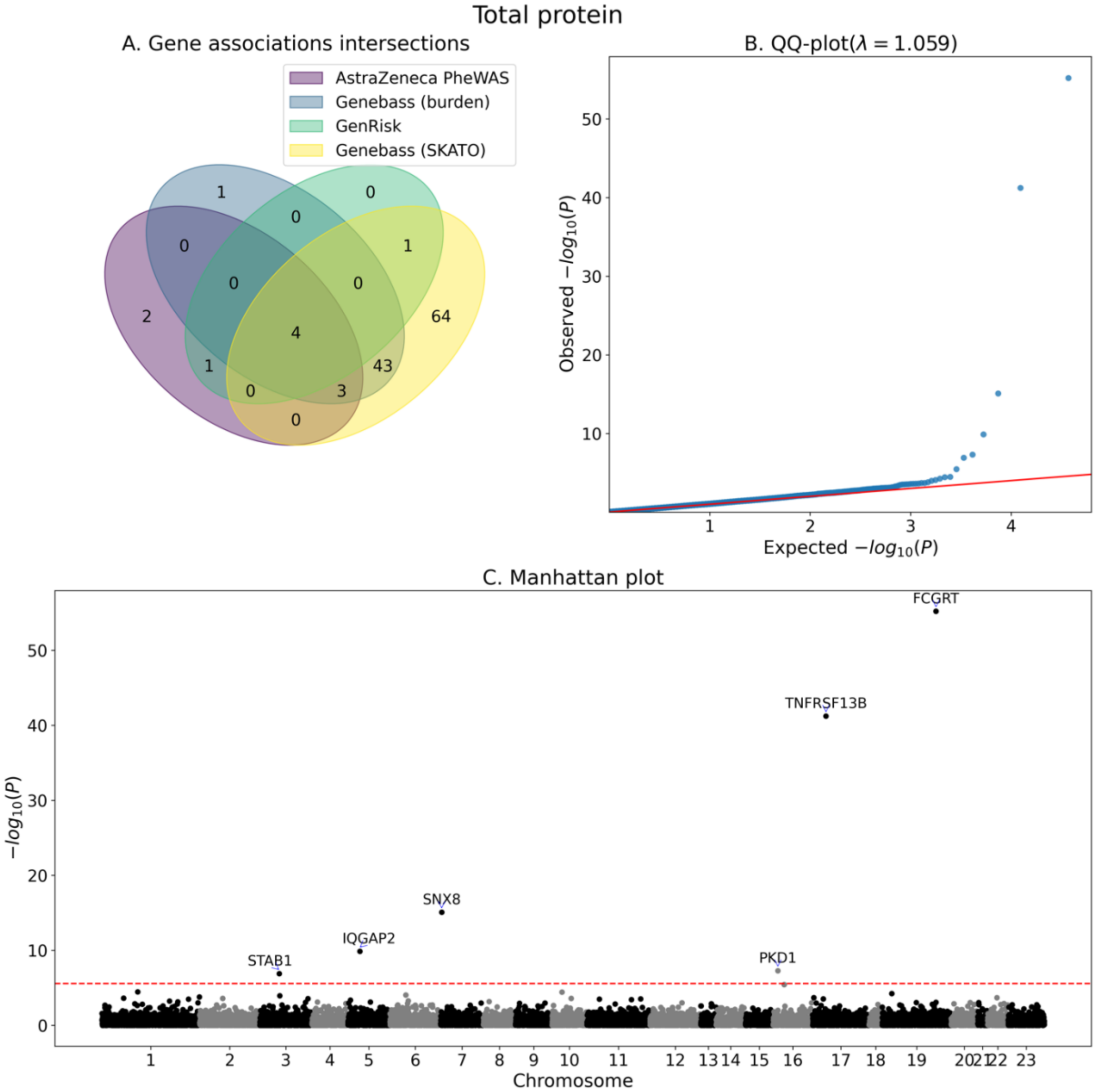

Figure S22 Association analysis summary for total protein.
A. Venn diagram of the number significantly associated genes as identified by GenRisk, AstraZenica PheWAS and genebass. B. QQ-plot of the P-values of GenRisk pipeline results. C. Manhattan plot of GenRisk pipeline results.

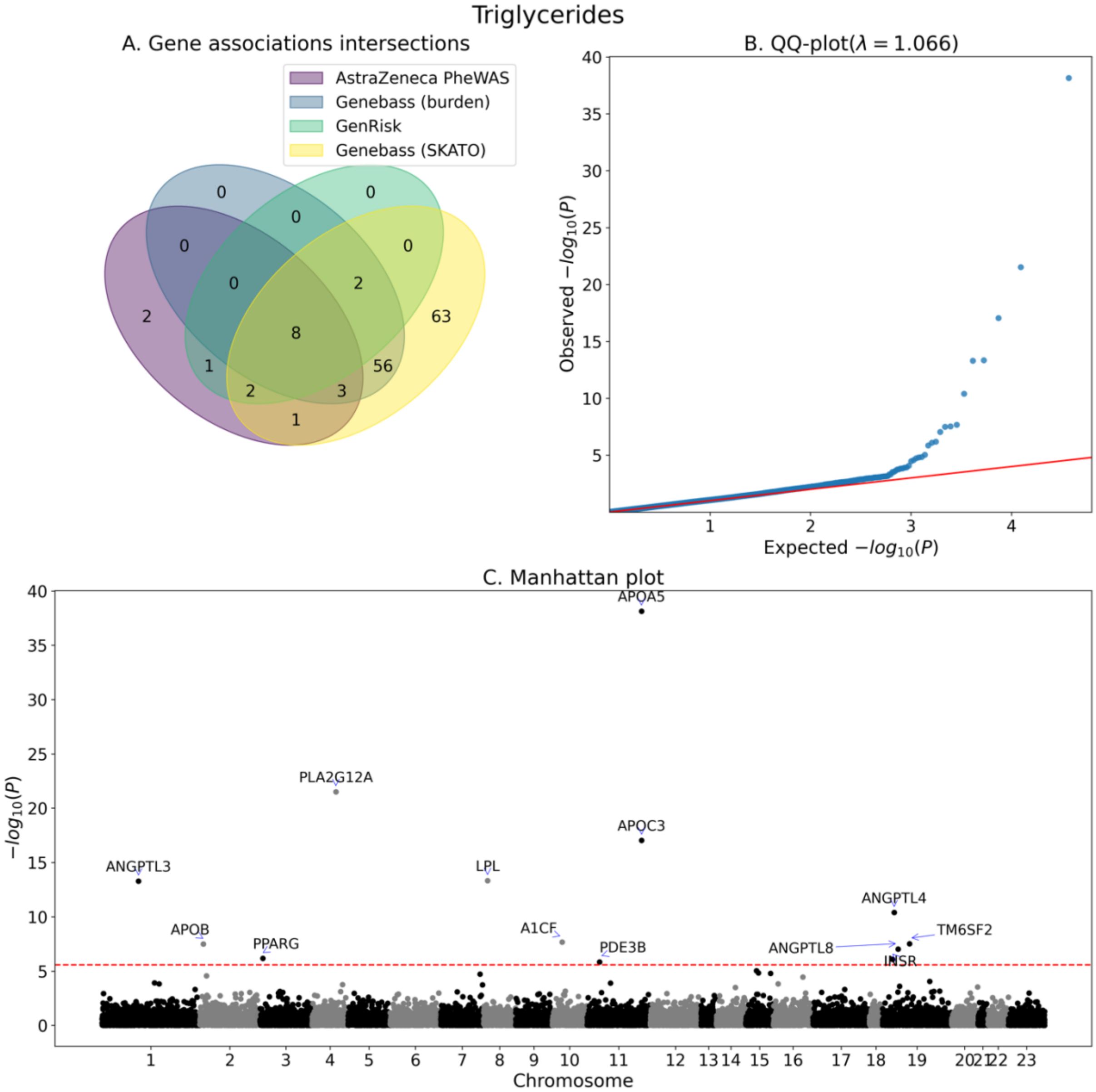

Figure S23 Association analysis summary for triglycerides.
A. Venn diagram of the number significantly associated genes as identified by GenRisk, AstraZenica PheWAS and genebass. B. QQ-plot of the P-values of GenRisk pipeline results. C. Manhattan plot of GenRisk pipeline results.

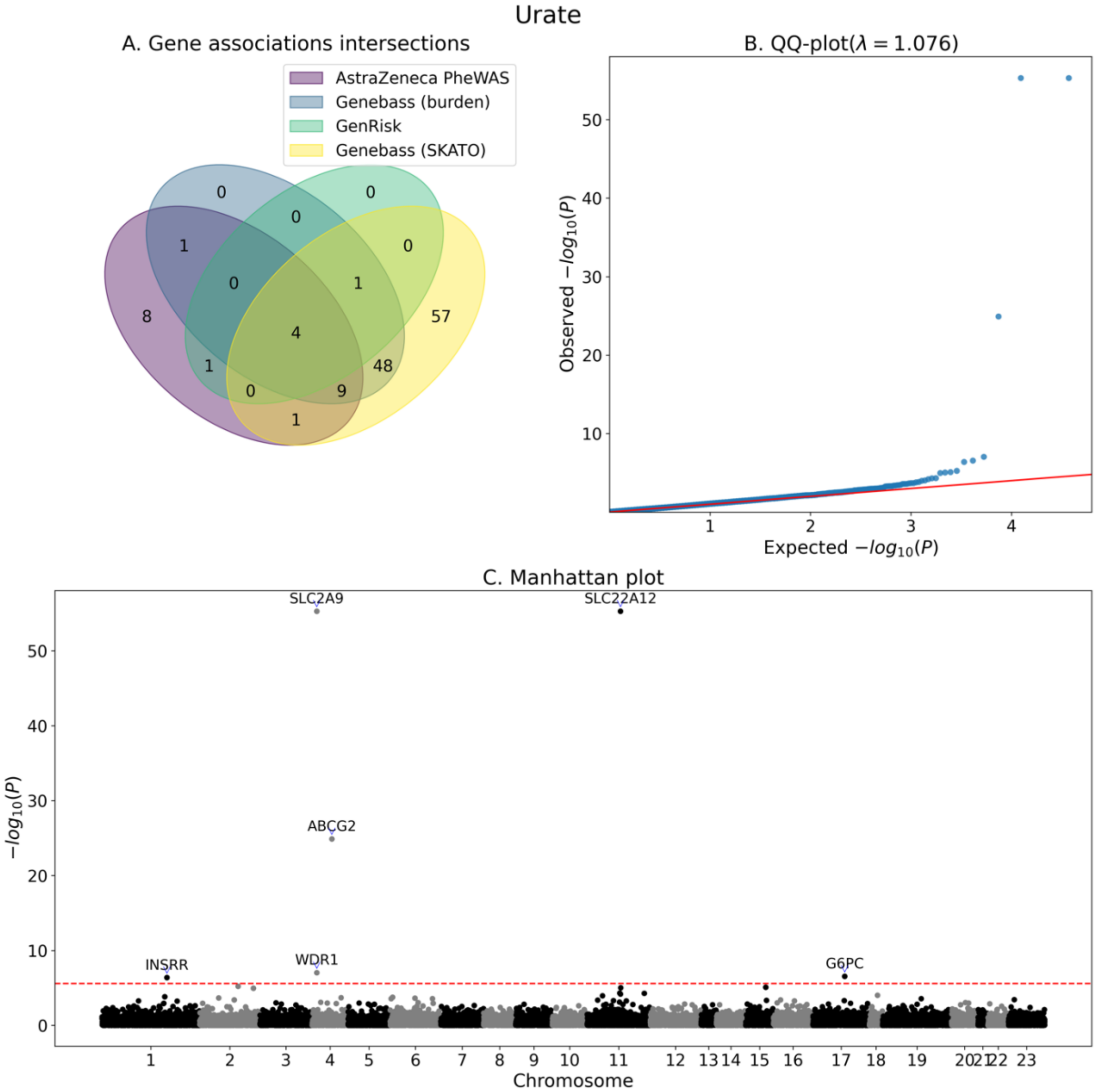

Figure S24 Association analysis summary for urate.
A. Venn diagram of the number significantly associated genes as identified by GenRisk, AstraZenica PheWAS and genebass. B. QQ-plot of the P-values of GenRisk pipeline results. C. Manhattan plot of GenRisk pipeline results

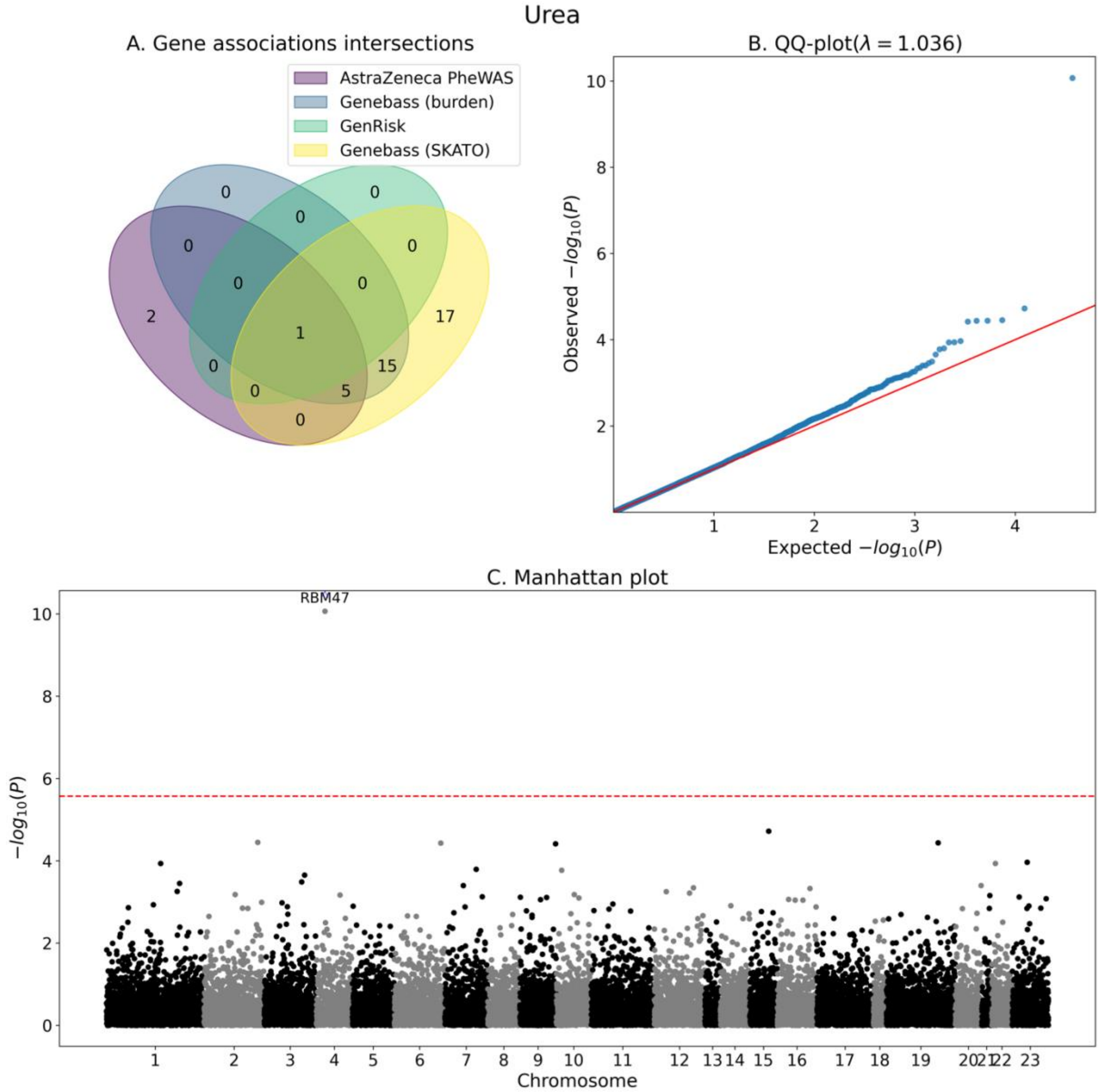

Figure S25 Association analysis summary for urea.
A. Venn diagram of the number significantly associated genes as identified by GenRisk, AstraZenica PheWAS and genebass. B. QQ-plot of the P-values of GenRisk pipeline results. C. Manhattan plot of GenRisk pipeline results

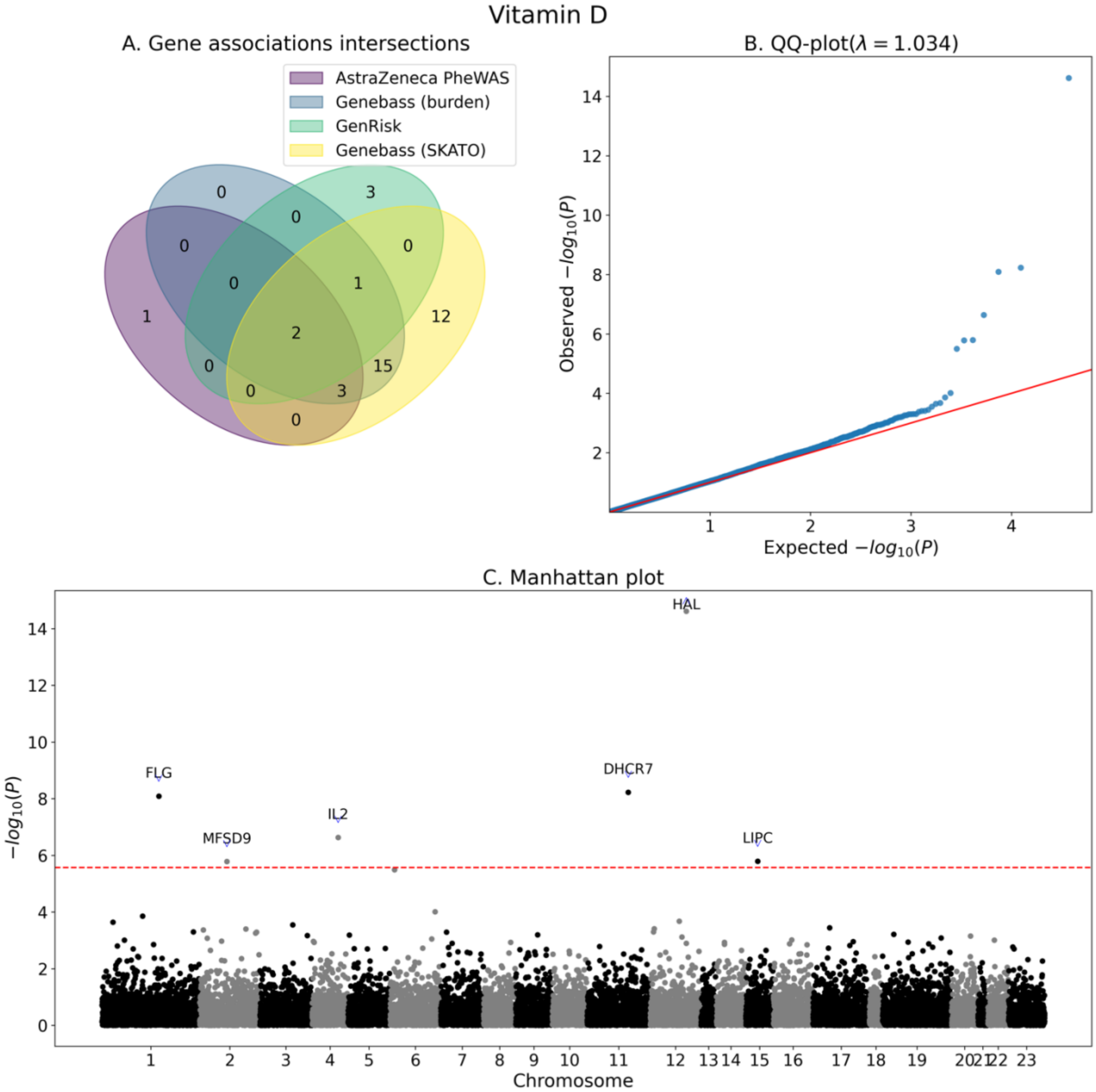

Figure S26 Association analysis summary for vitamin D.
A. Venn diagram of the number significantly associated genes as identified by GenRisk, AstraZenica PheWAS and genebass. B. QQ-plot of the P-values of GenRisk pipeline results. C. Manhattan plot of GenRisk pipeline results.

A.Alanine aminotransferase cov model

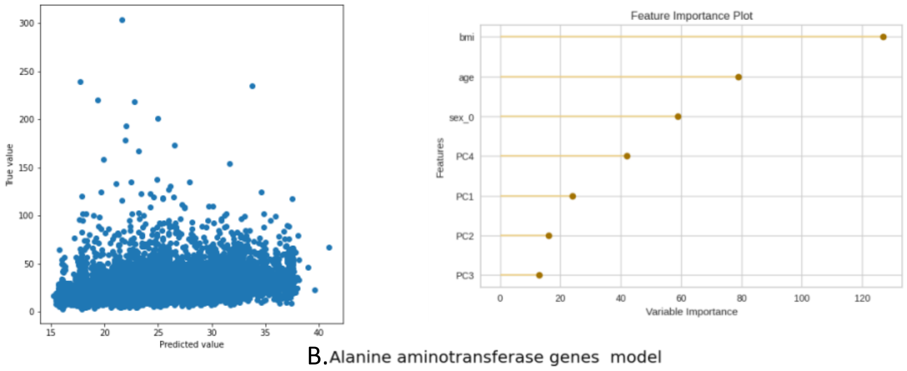

B.Alanine aminotransferase genes model

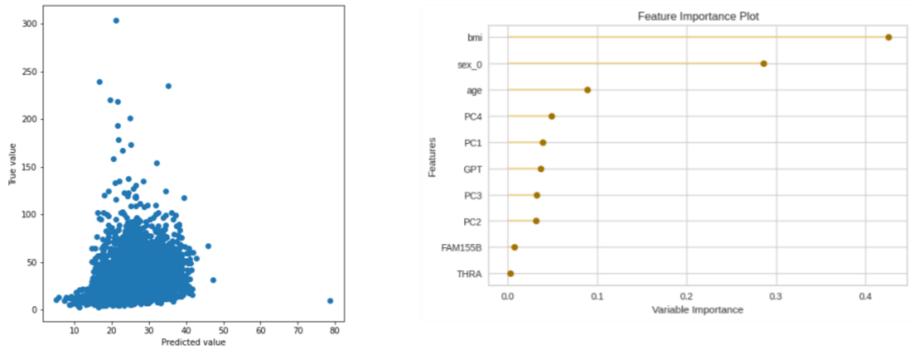

C. Alanine aminotransferase prs model

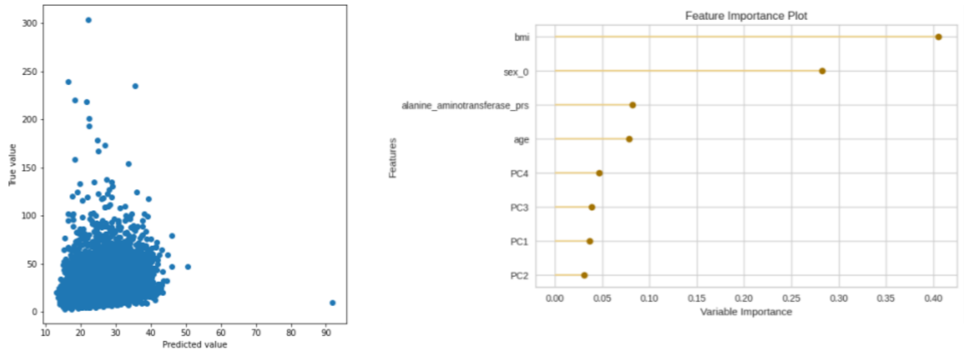

D. Alanine aminotransferase genes prs model

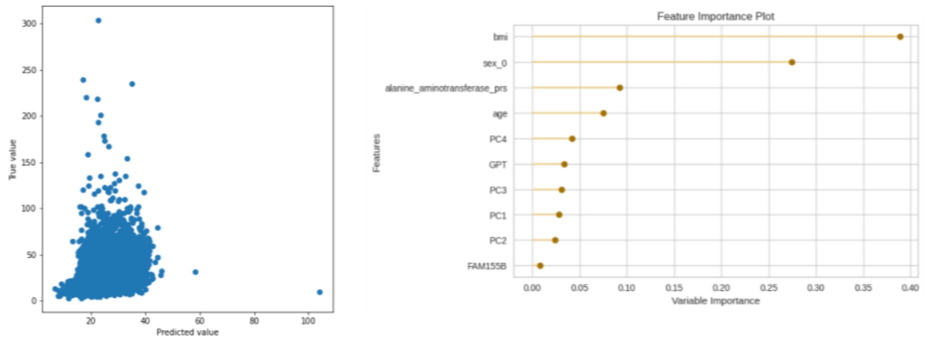

Figure S27 True vs. Predicted value plot (left) and top 10 features (right) for alanine aminotransferase A. covariates model B. genes model C. PRS model and D. combined model.

A. Albumin cov model

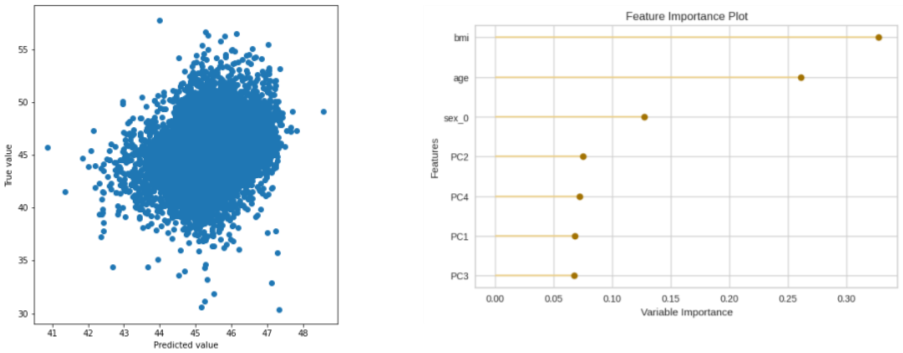

B. Albumin genes model

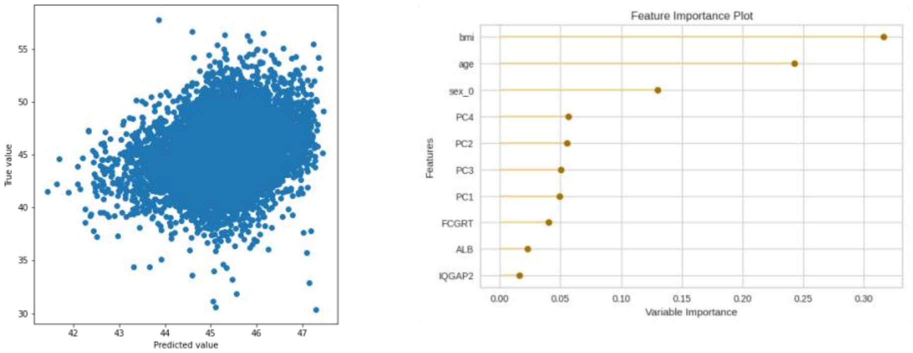

C. Albumin prs model

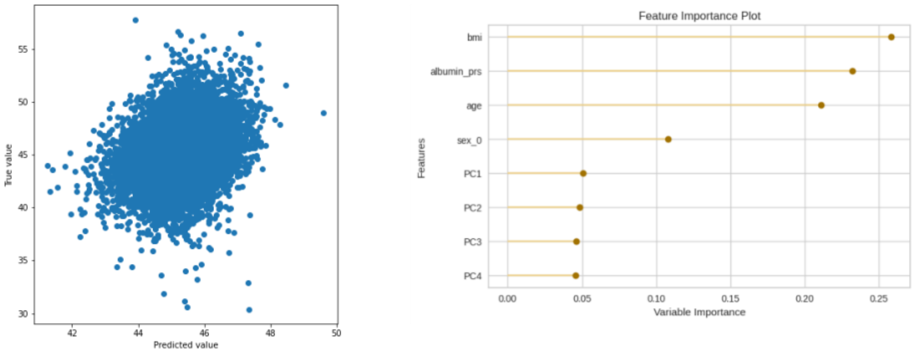

D. Albumin genes prs model

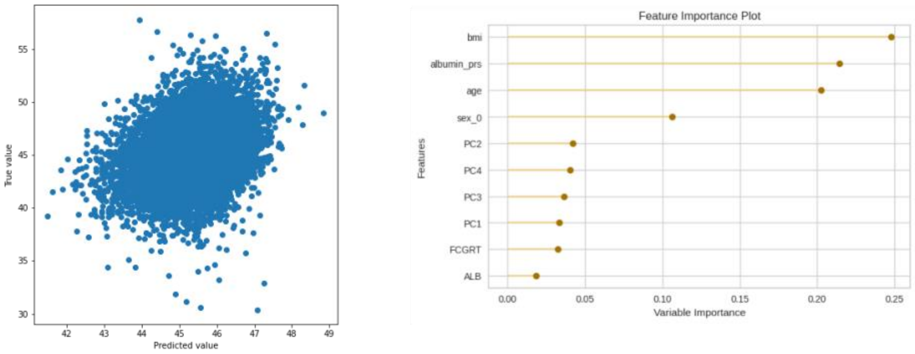

Figure S28 True vs. Predicted value plot (left) and top 10 features (right) for albumin A. covariates model B. genes model C. PRS model and D. combined model.

A. Alkaline phosphatase cov model

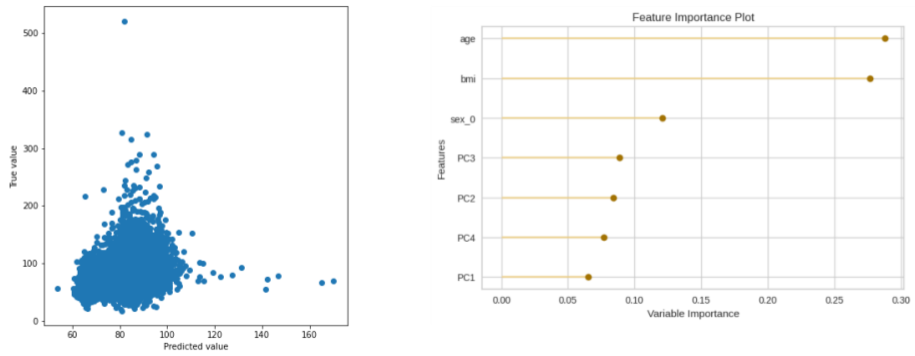

B. Alkaline phosphatase genes model

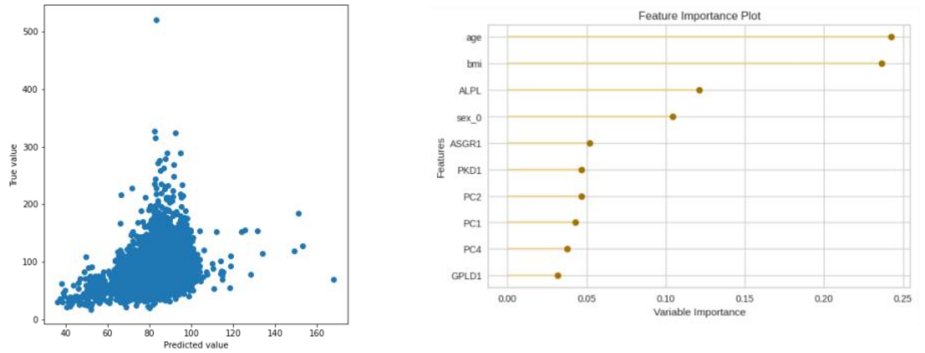

C. Alkaline phosphatase prs model

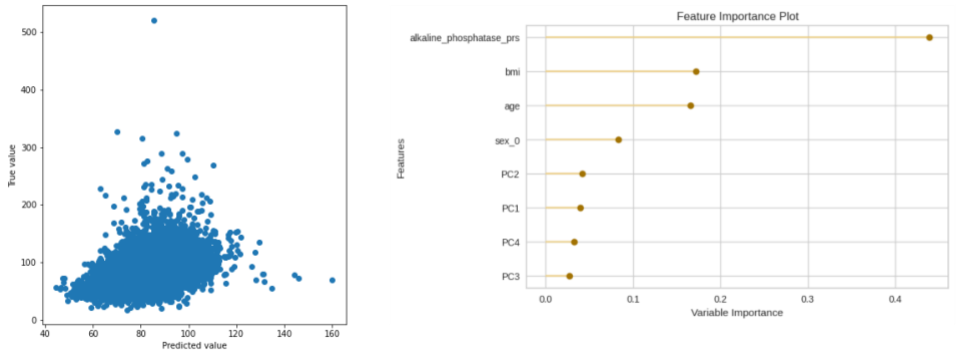

D. Alkaline phosphatase genes prs model

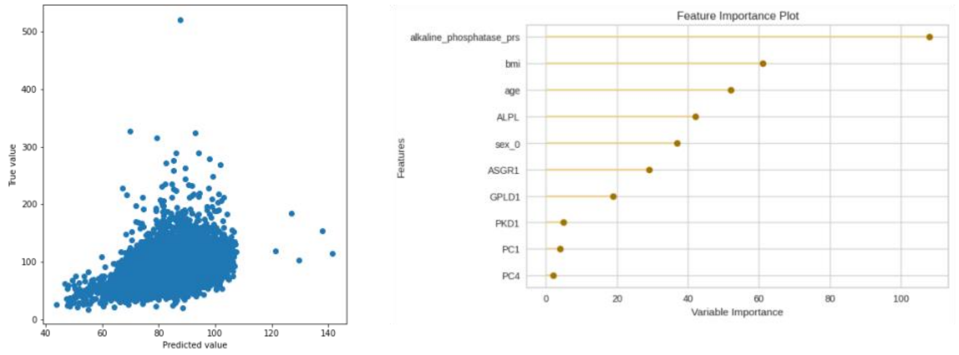

Figure S29 True vs. Predicted value plot (left) and top 10 features (right) for alkaline phosphatase A. covariates model B. genes model C. PRS model and D. combined model.

A. Apolipoprotein a cov model

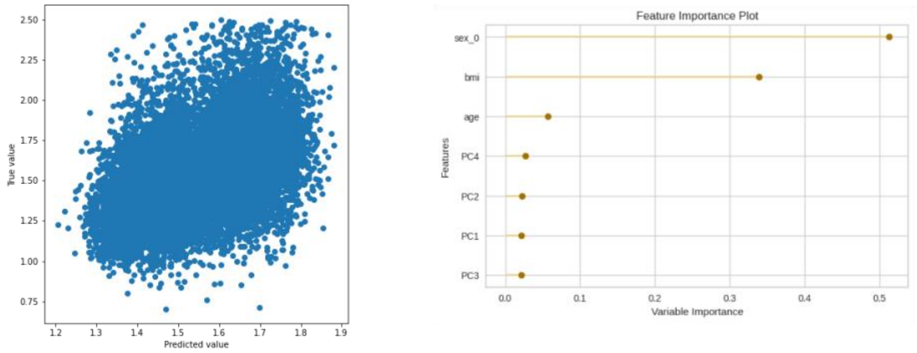

B. Apolipoprotein a genes model

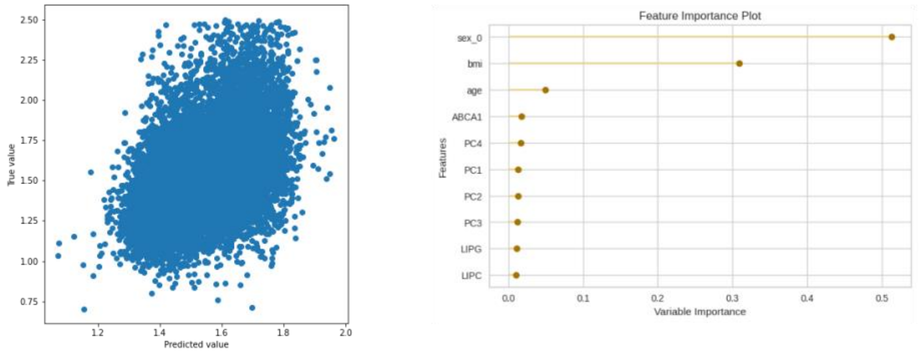

C. Apolipoprotein a prs model

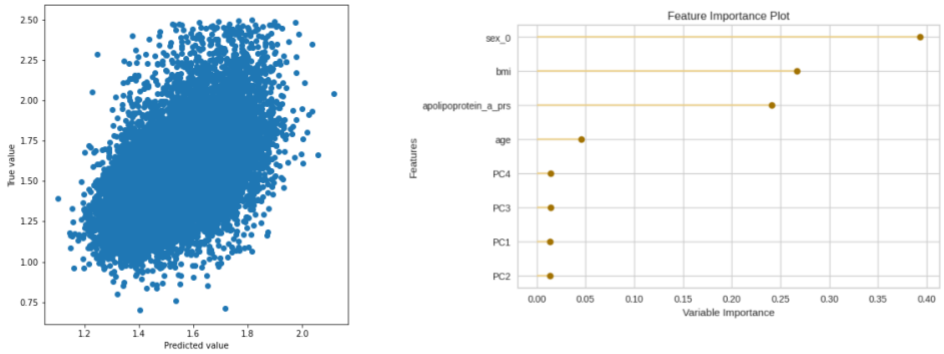

D. Apolipoprotein a genes prs model

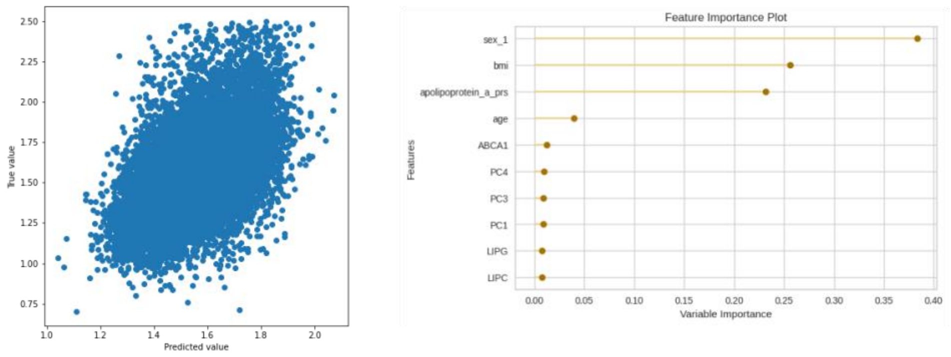

Figure S30 True vs. Predicted value plot (left) and top 10 features (right) for apolipoprotein A A. covariates model B. genes model C. PRS model and D. combined model.

A. Apolipoprotein b * cov model

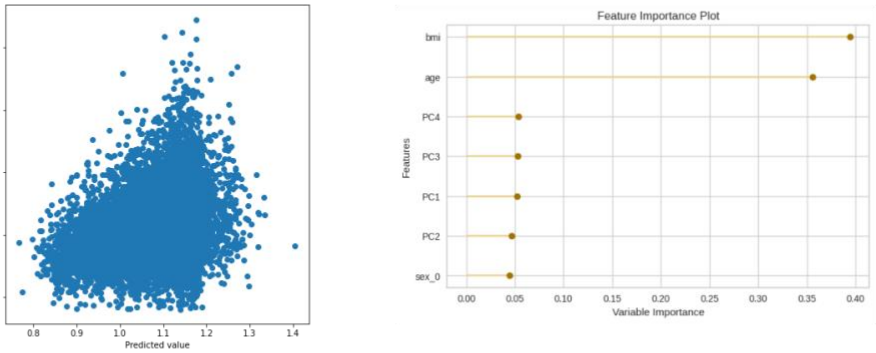

B.Apolipoprotein b * genes model

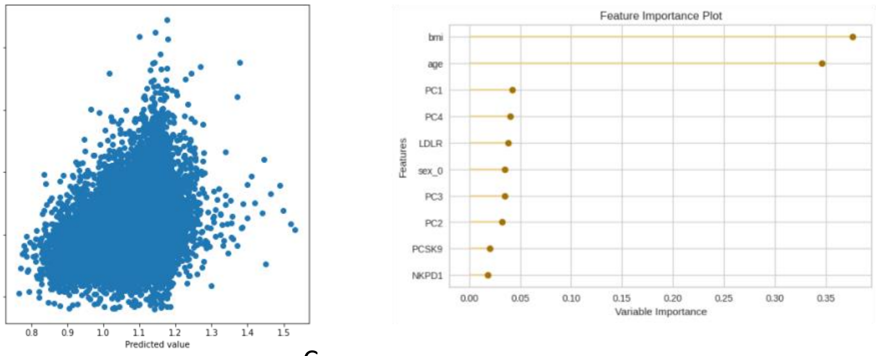

C. Apolipoprotein b * prs model

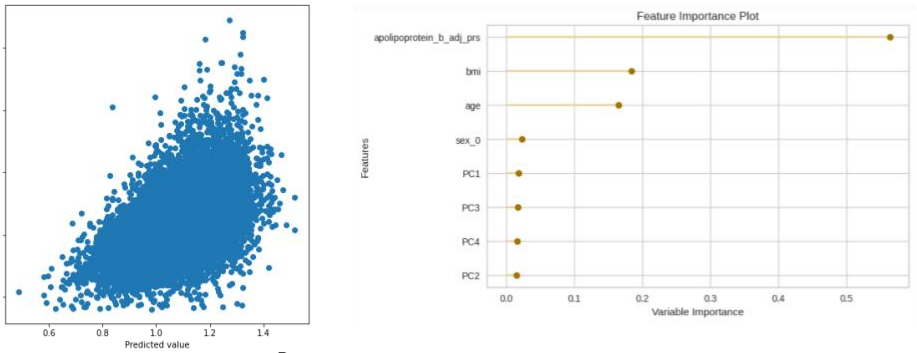

D. Apolipoprotein b * genes prs model

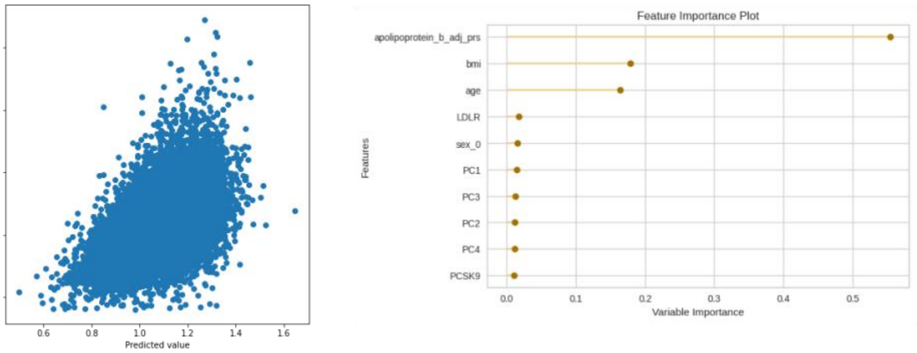

Figure S31 True vs. Predicted value plot (left) and top 10 features (right) for apolipoprotein B* A. covariates model B. genes model C. PRS model and D. combined model.
* statin adjusted values

A. Aspartate aminotransferase cov model

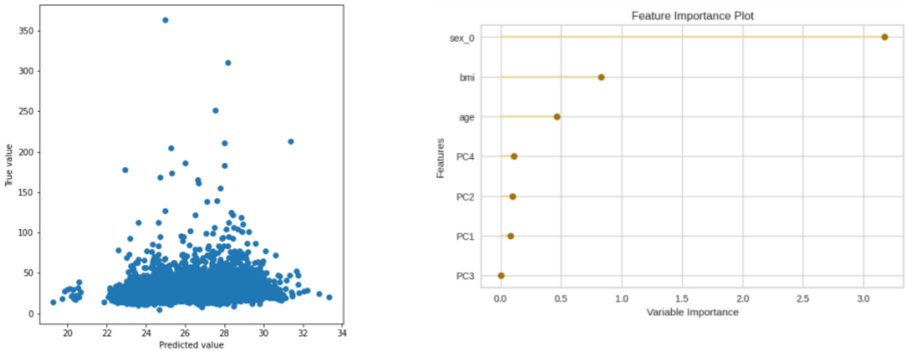

B. Aspartate aminotransferase genes model

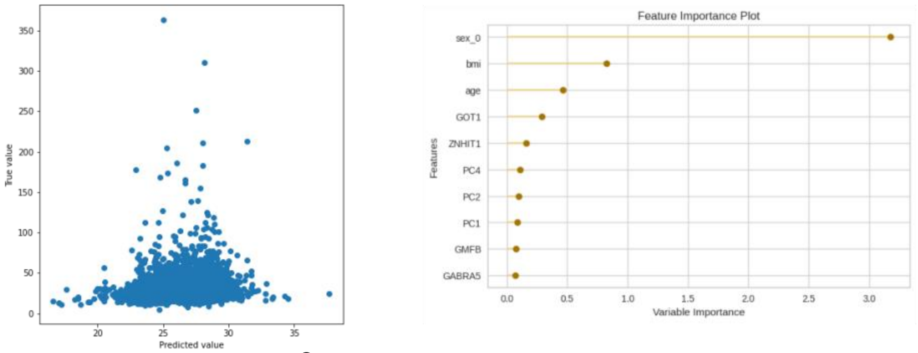

C. Aspartate aminotransferase prs model

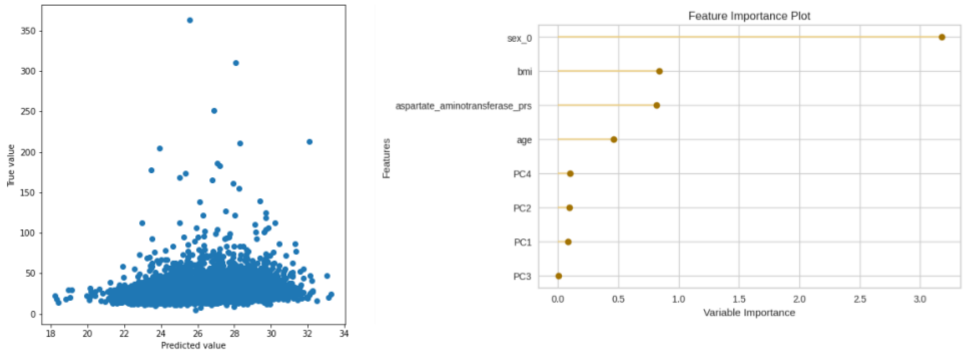

D. Aspartate aminotransferase genes prs model

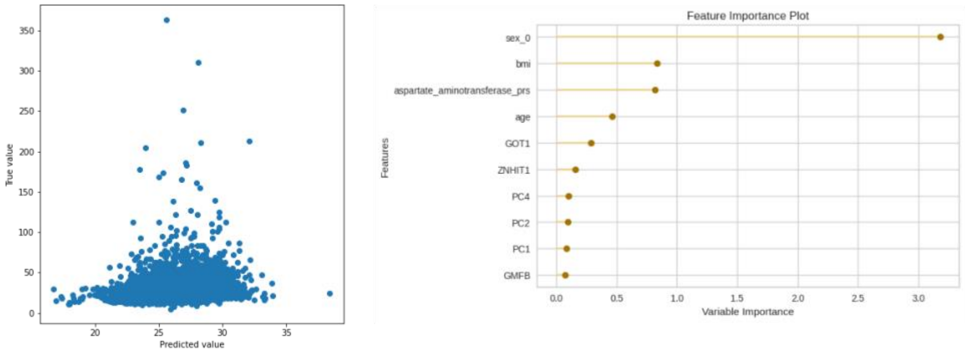

Figure S32 True vs. Predicted value plot (left) and top 10 features (right) for aspartate aminotransferase A. covariates model B. genes model C. PRS model and D. combined model.

A. Calcium cov model

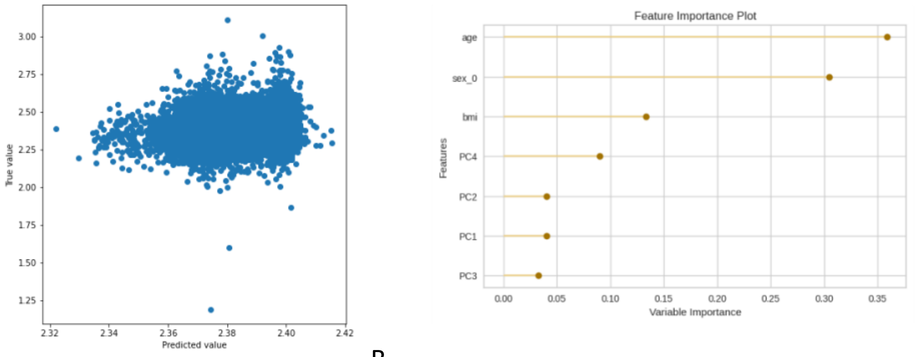

B. Calcium genes model

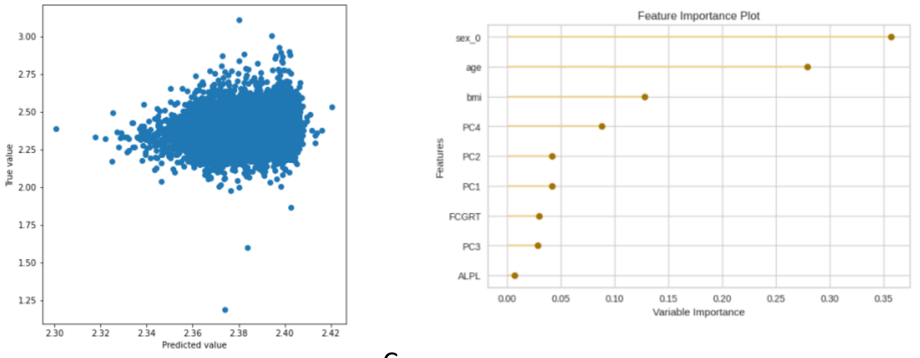

C. Calcium prs model

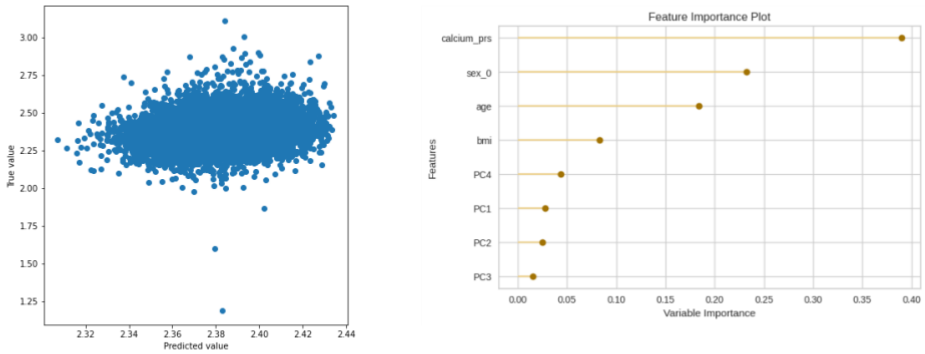

D. Calcium genes prs model

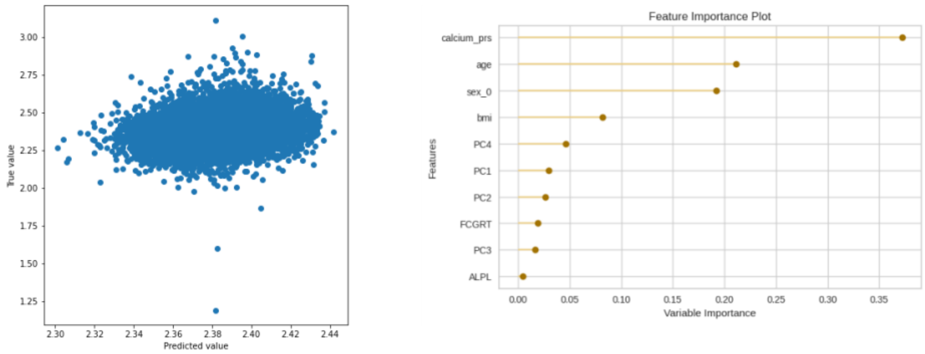

Figure S33 True vs. Predicted value plot (left) and top 10 features (right) for calcium A. covariates model B. genes model C. PRS model and D. combined model.

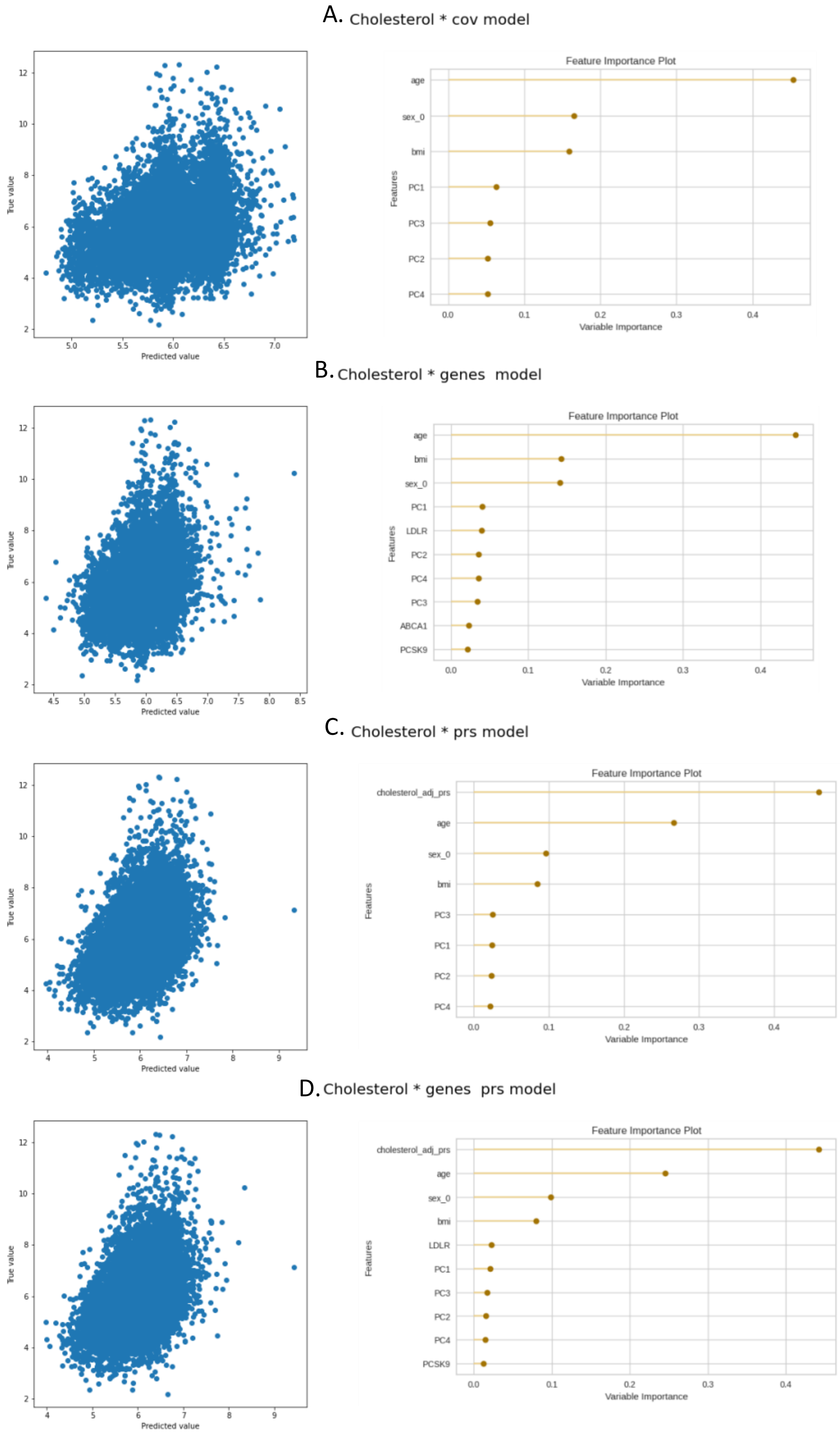

Figure S 34 True vs. Predicted value plot (left) and top 10 features (right) for cholesterol* A. covariates model B. genes model C. PRS model and D. combined model.
* statin adjusted values

A. Creactive protein cov model

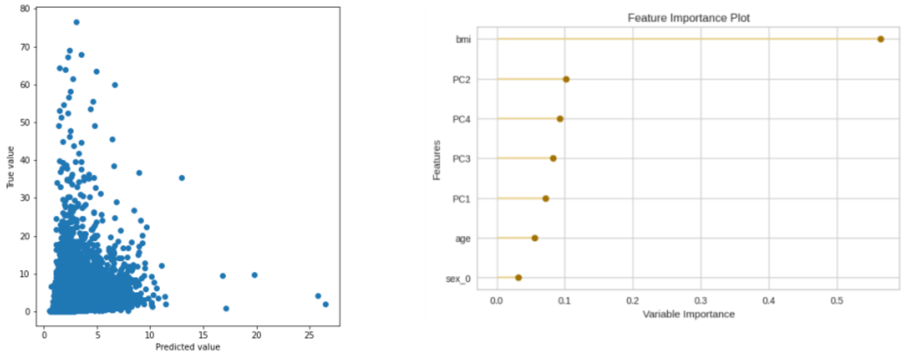

B. Creactive protein genes model

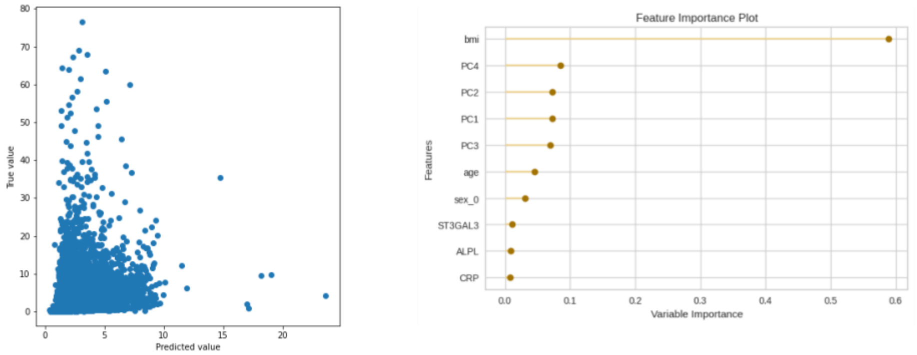

C. Creactive protein prs model

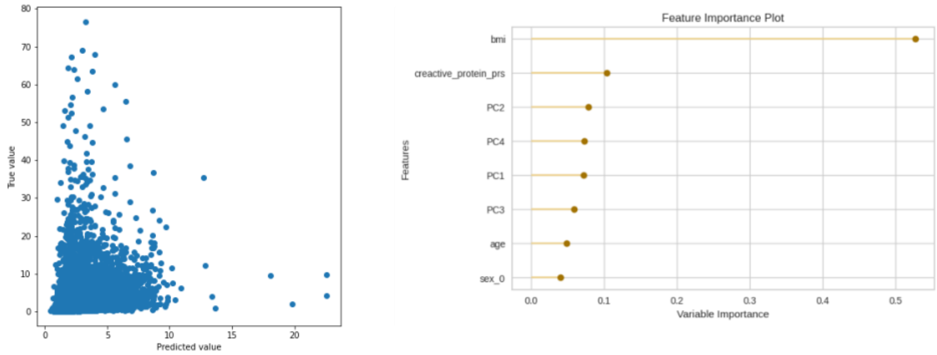

D. Creactive protein genes prs model

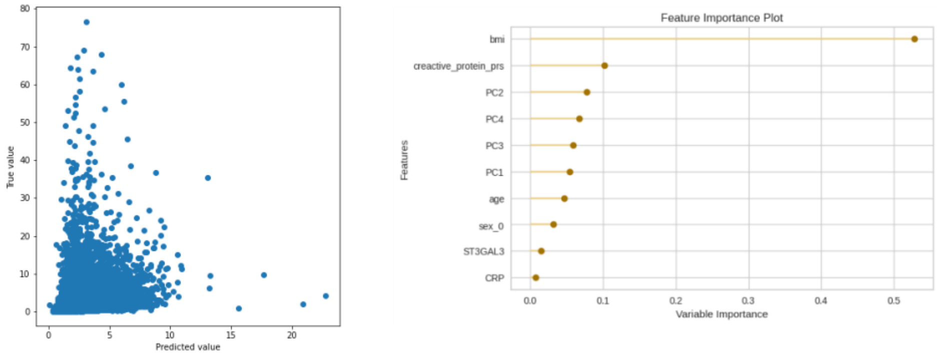

Figure S35 True vs. Predicted value plot (left) and top 10 features (right) for C reactive protein A. covariates model B. genes model C. PRS model and D. combined model.

A. Creatinine cov model

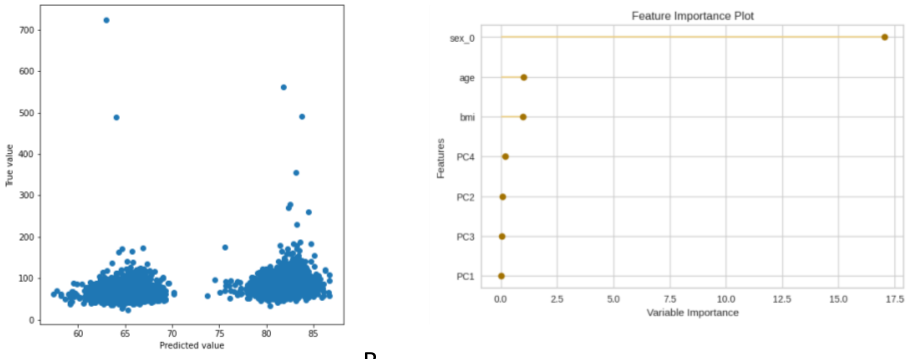

B. Creatinine genes model

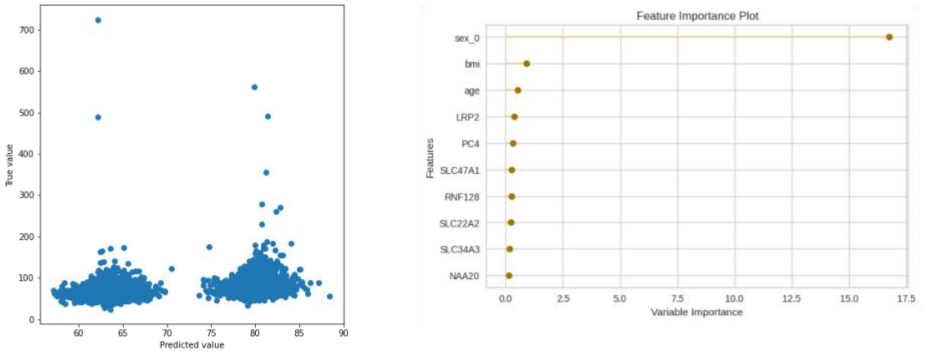

C. Creatinine prs model

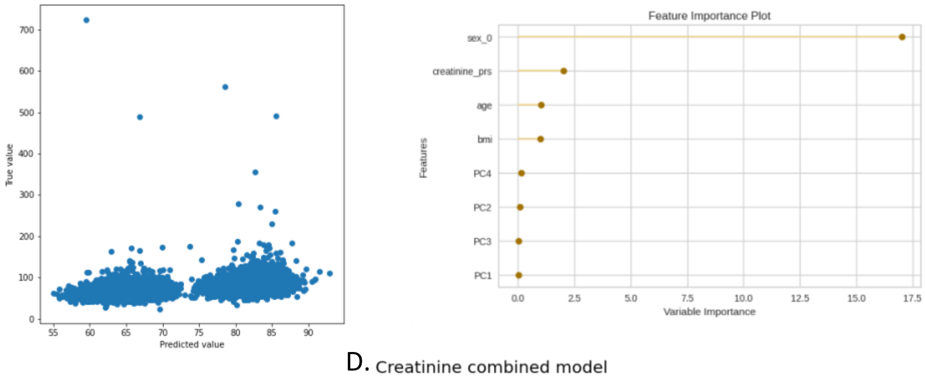

D. Creatinine combined model

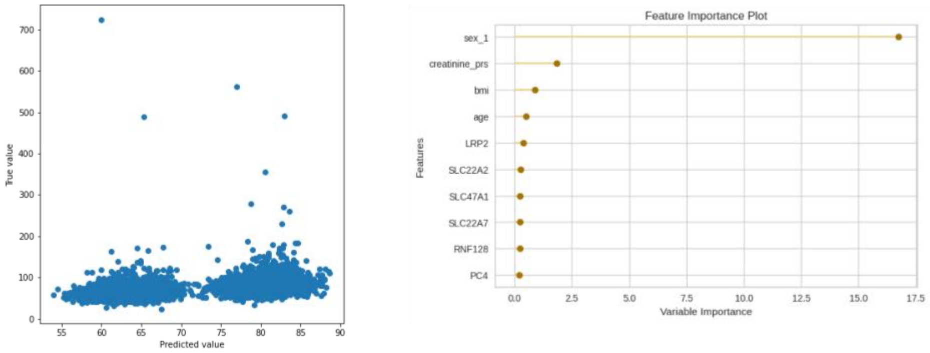

Figure S36 True vs. Predicted value plot (left) and top 10 features (right) for creatinine A. covariates model B. genes model C. PRS model and D. combined model.

A. Cystatin c cov model

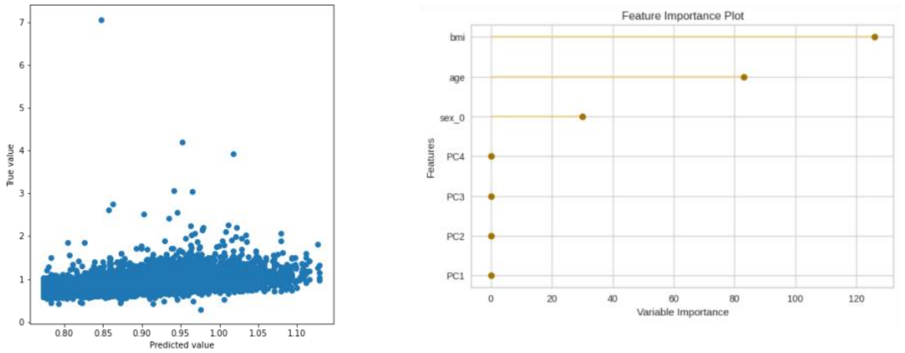

B. Cystatin c genes model

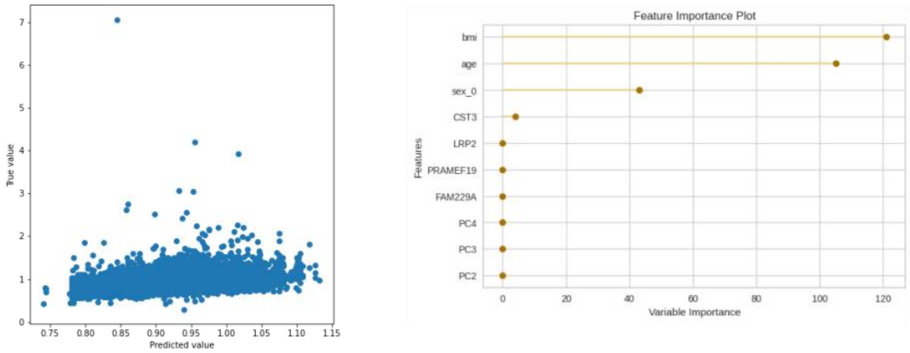

C. Cystatin c prs model

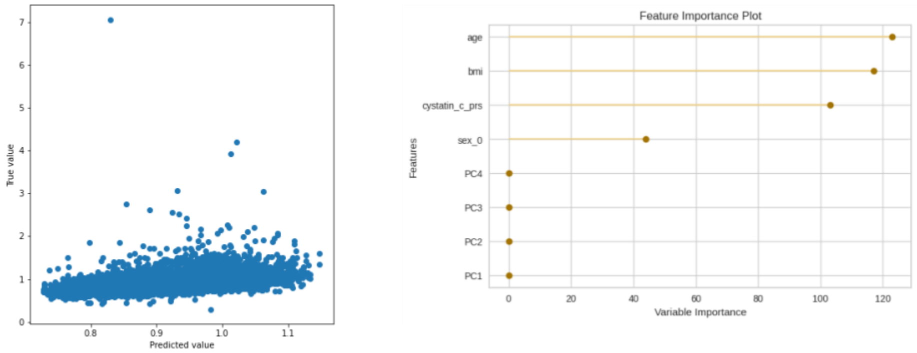

D. Cystatin c genes prs model

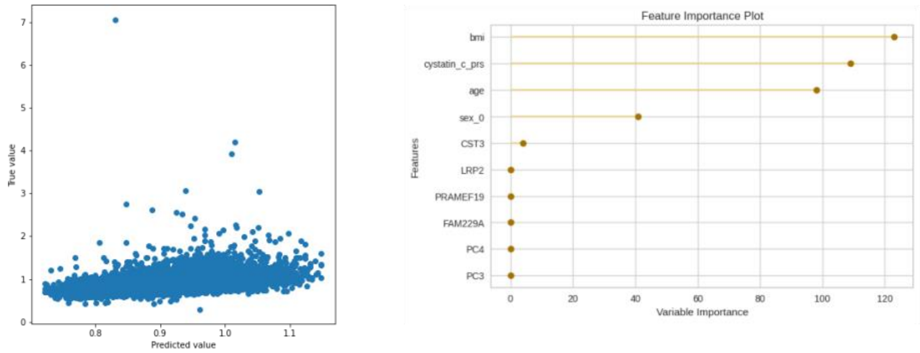

Figure S37 True vs. Predicted value plot (left) and top 10 features (right) for cystatin C A. covariates model B. genes model C. PRS model and D. combined model.

A. Direct bilirubin cov model

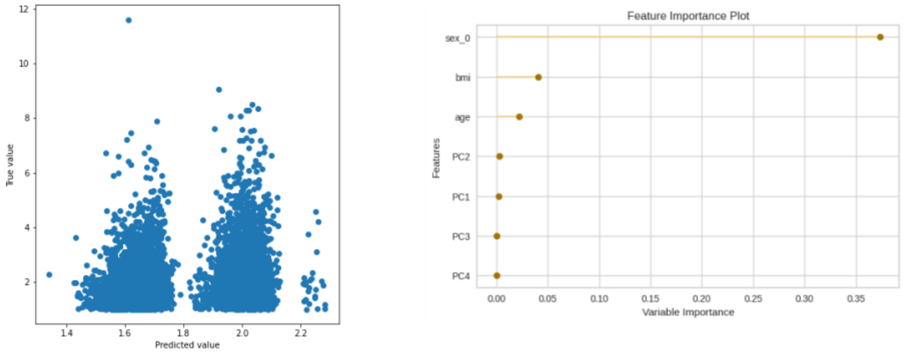

B. Direct bilirubin genes model

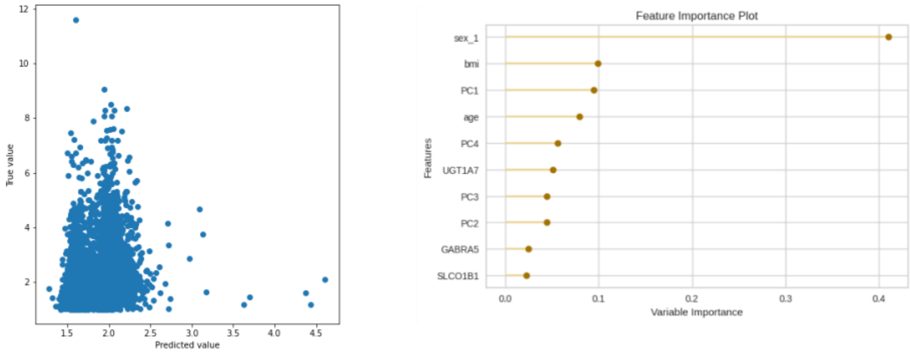

C. Direct bilirubin prs model

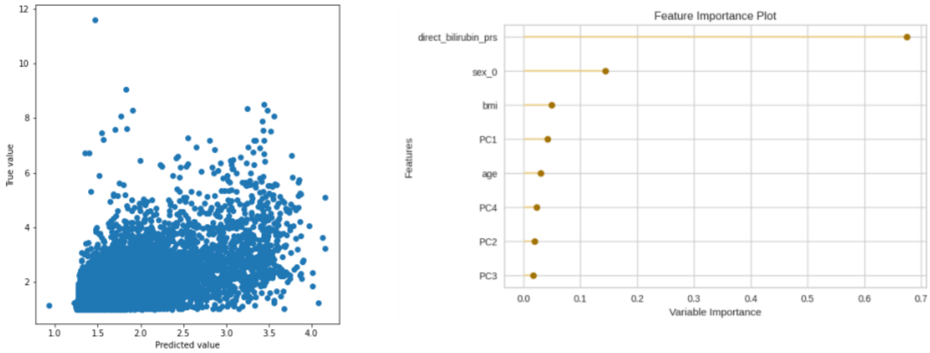

D. Direct bilirubin genes prs model

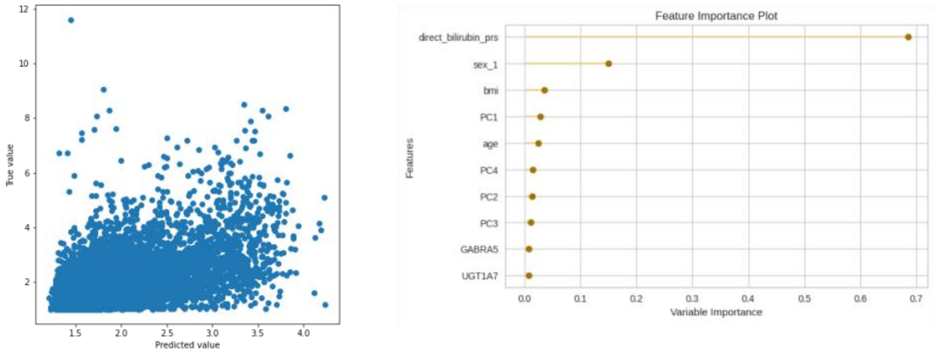

Figure S38 True vs. Predicted value plot (left) and top 10 features (right) for direct bilirubin A. covariates model B. genes model C. PRS model and D. combined model.

A. Gamma glutamyltransferase cov model

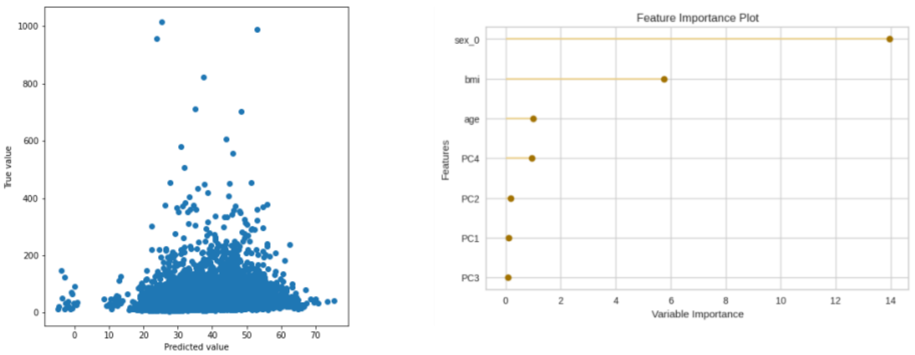

B. Gamma glutamyltransferase genes model

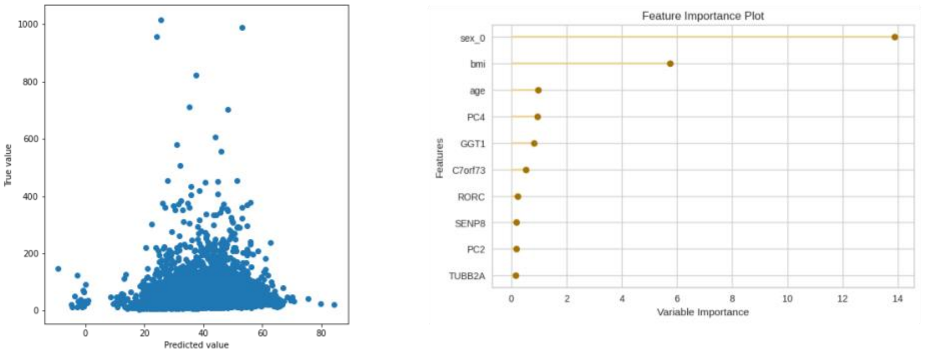

C. Gamma glutamyltransferase prs model

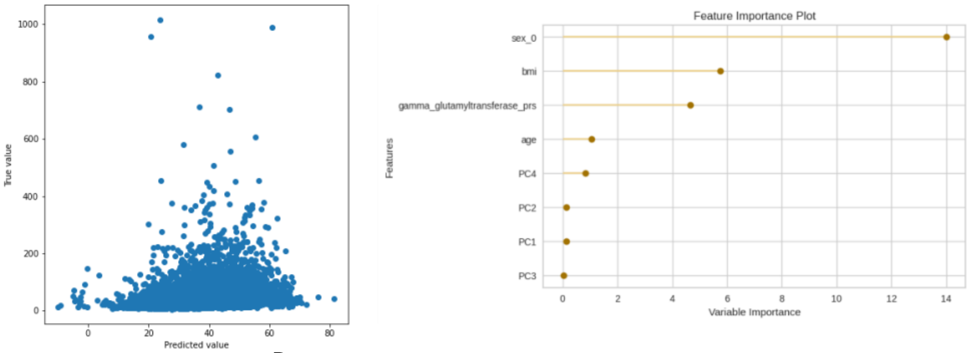

D. Gamma glutamyltransferase genes prs model

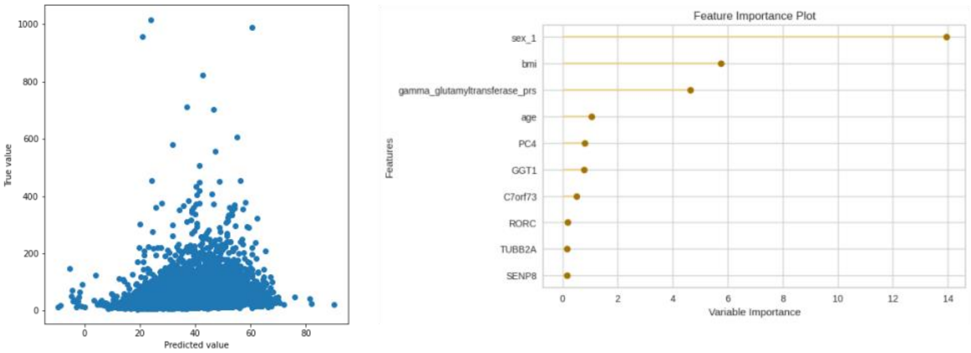

Figure S39 True vs. Predicted value plot (left) and top 10 features (right) for gamma glutamyltransferase A. covariates model B. genes model C. PRS model and D. combined model.

A. Glucose cov model

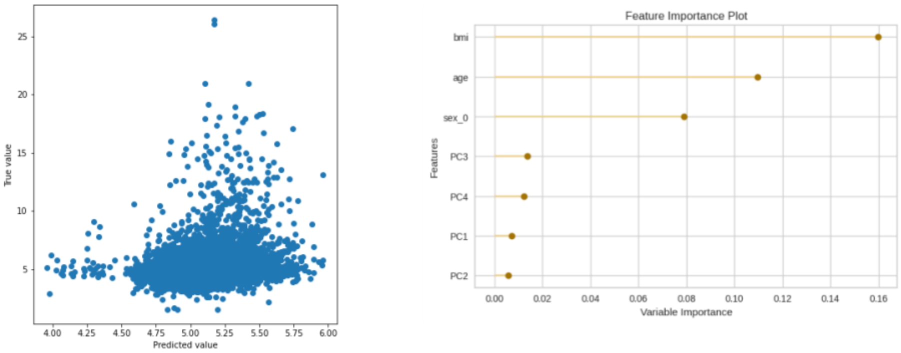

B. Glucose genes model

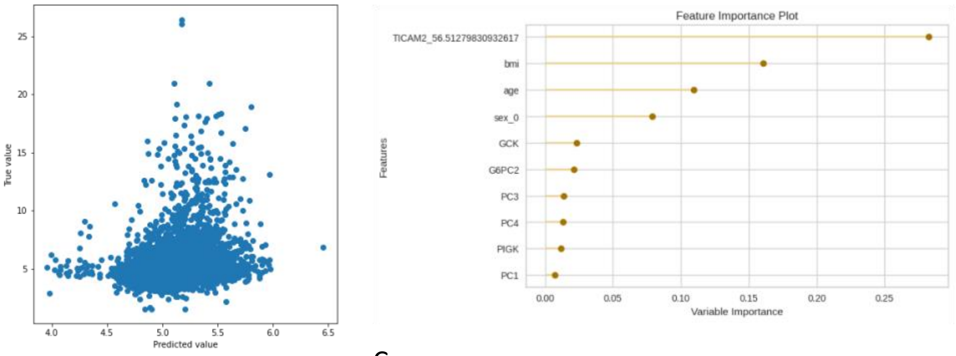

C. Glucose prs model

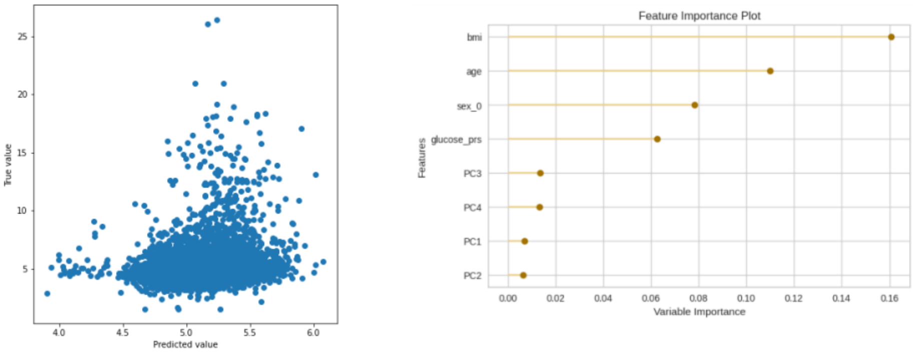

D. Glucose genes prs model

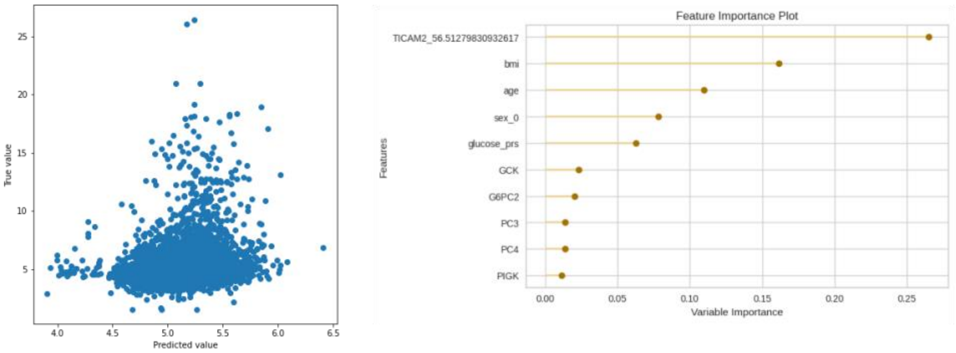

Figure S40 True vs. Predicted value plot (left) and top 10 features (right) for glucose A. covariates model B. genes model C. PRS model and D. combined model.

A. Glycated haemoglobin (hba1c) cov model

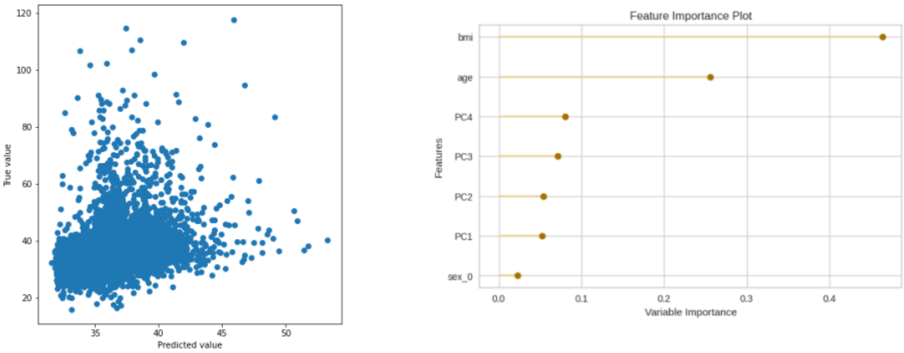

B.Glycated haemoglobin (hba1c) genes model

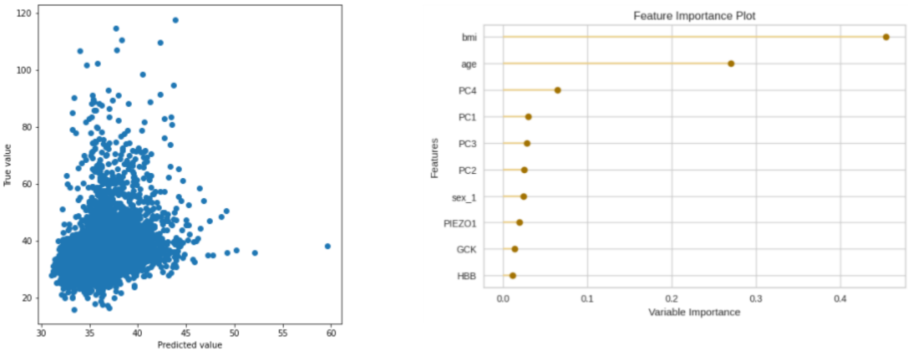

C. Glycated haemoglobin (hba1c) prs model

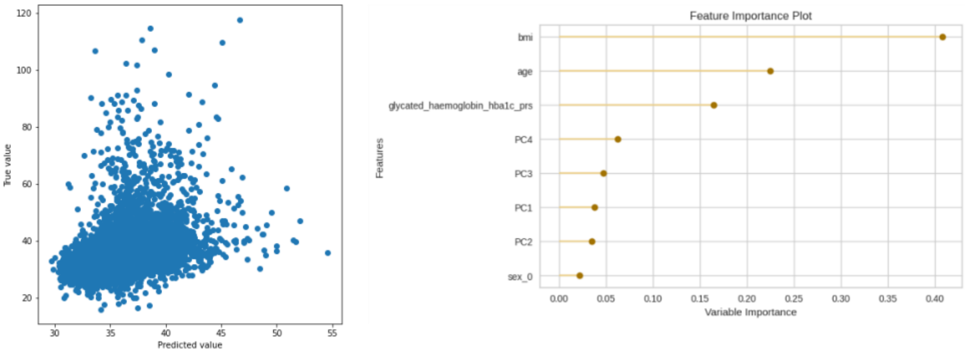

D.Glycated haemoglobin (hba1c) genes prs model

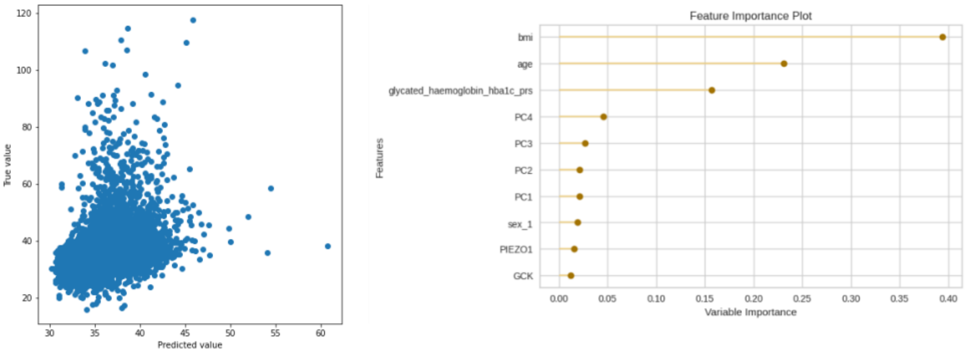

Figure S41 True vs. Predicted value plot (left) and top 10 features (right) for glycated haemoglobin (HbA1c) A. covariates model B. genes model C. PRS model and D. combined model.

A. Hdl cholesterol cov model

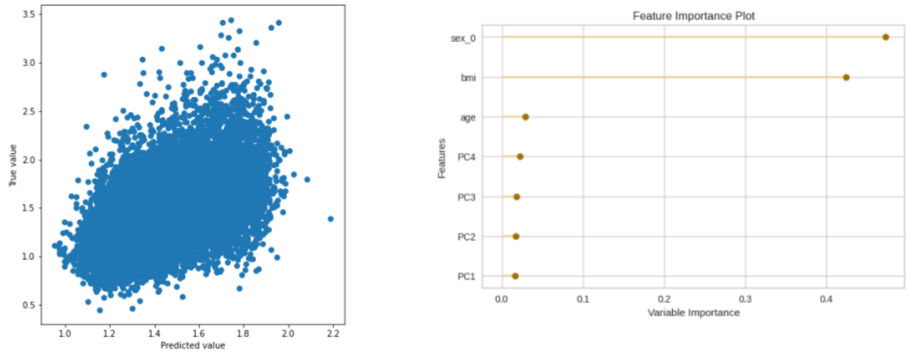

B. Hdl cholesterol genes model

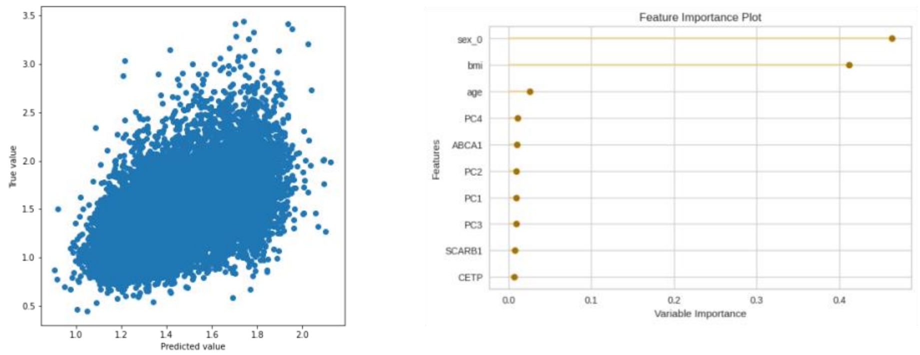

C. Hdl cholesterol prs model

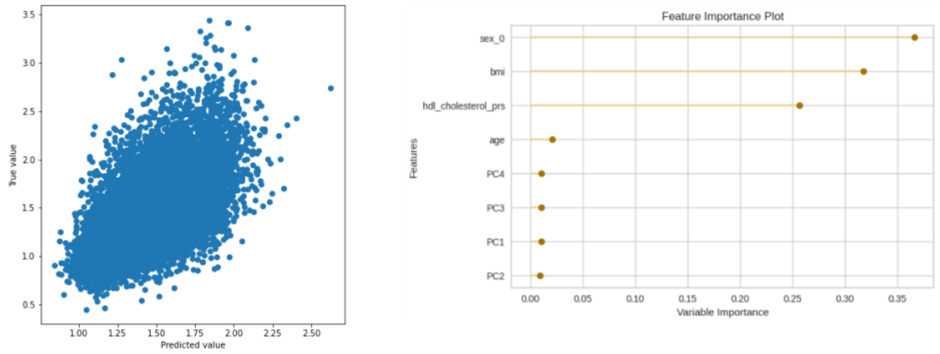

D. Hdl cholesterol genes prs model

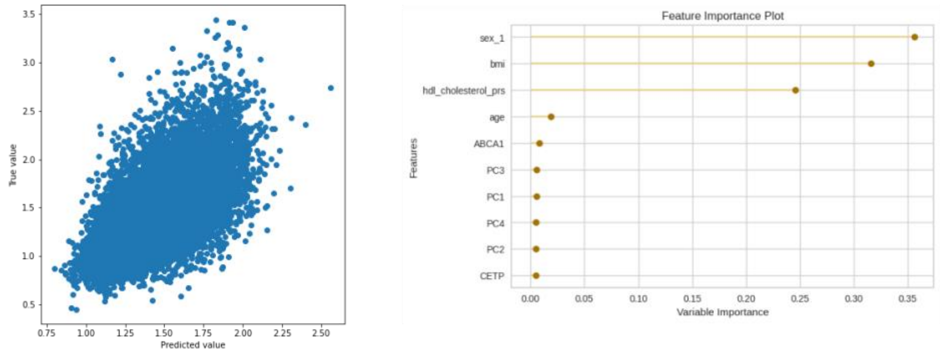

Figure S42 True vs. Predicted value plot (left) and top 10 features (right) for HDL cholesterol A. covariates model B. genes model C. PRS model and D. combined model.

A. Igf1 cov model

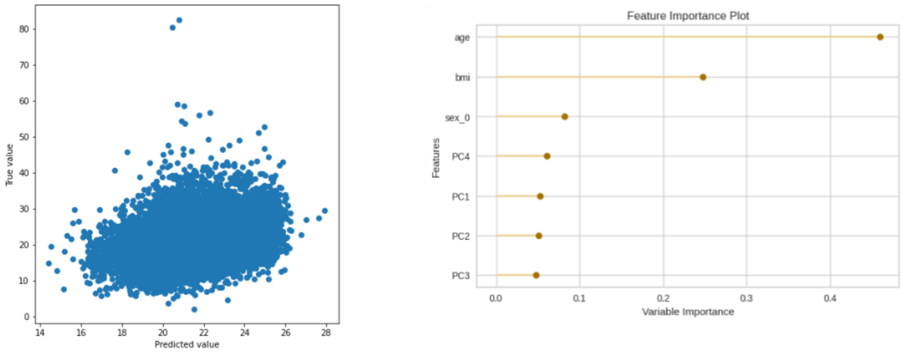

B. Igf1 genes model

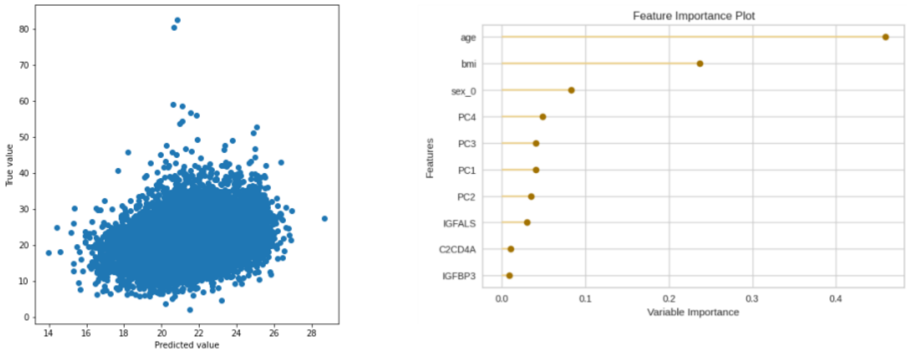

C. Igf1 prs model

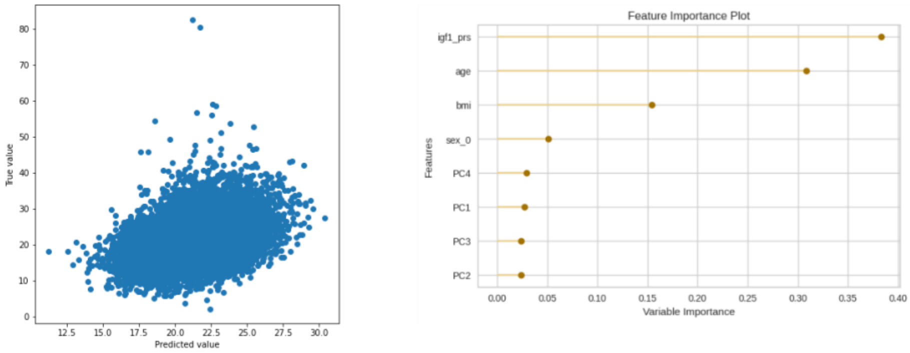

D. Igf1 genes prs model

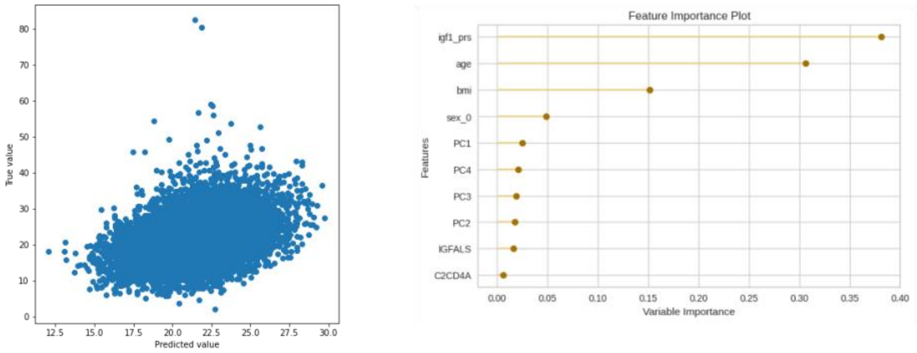

Figure S43 True vs. Predicted value plot (left) and top 10 features (right) for IGF1 A. covariates model B. genes model C. PRS model and D. combined model.

A. Ldl direct * cov model

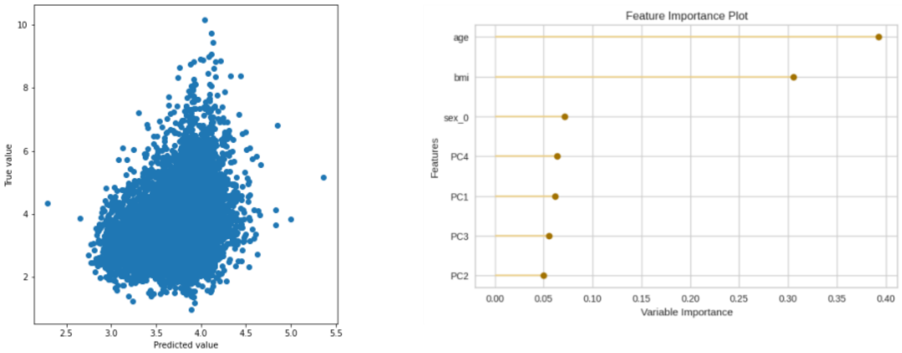

B. Ldl direct * genes model

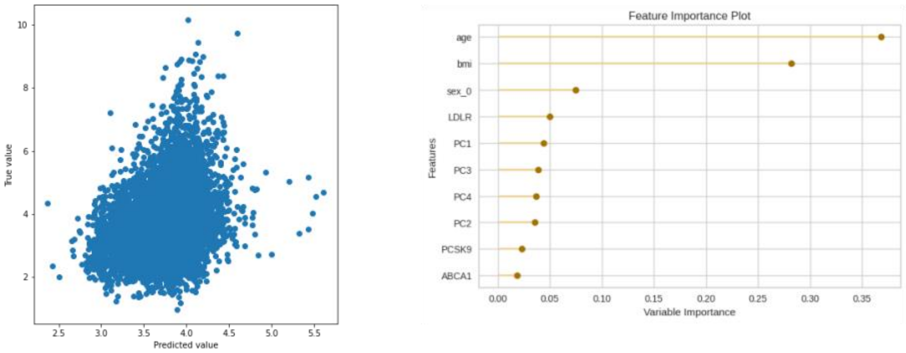

C. Ldl direct * prs model

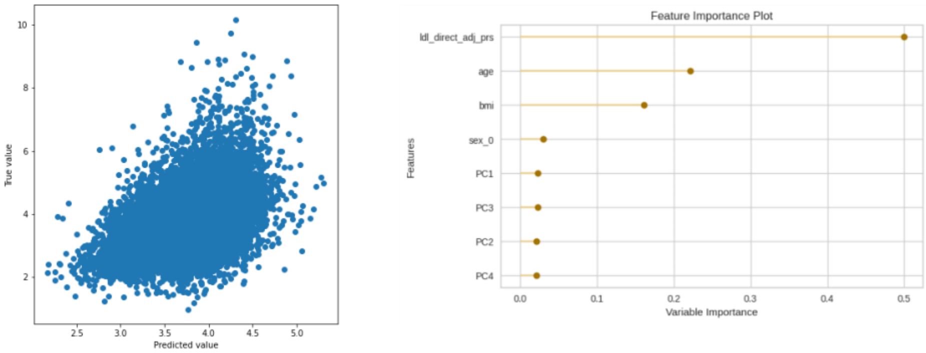

D. Ldl direct * genes prs model

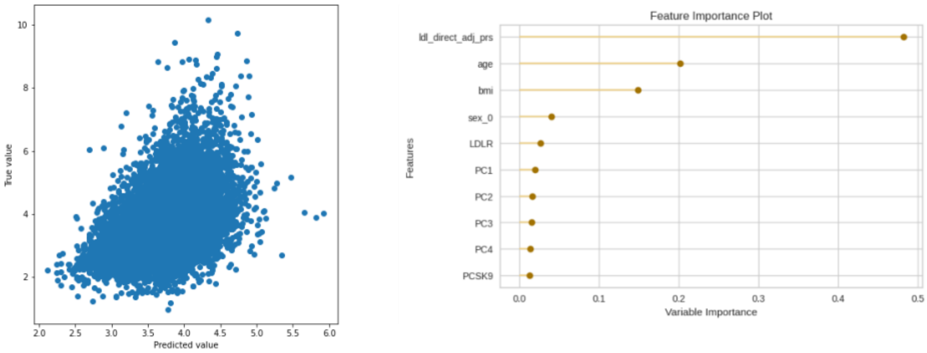

Figure S44 True vs. Predicted value plot (left) and top 10 features (right) for LDL direct* A. covariates model B. genes model C. PRS model and D. combined model.
* statin adjusted values

A. Lipoprotein a cov model

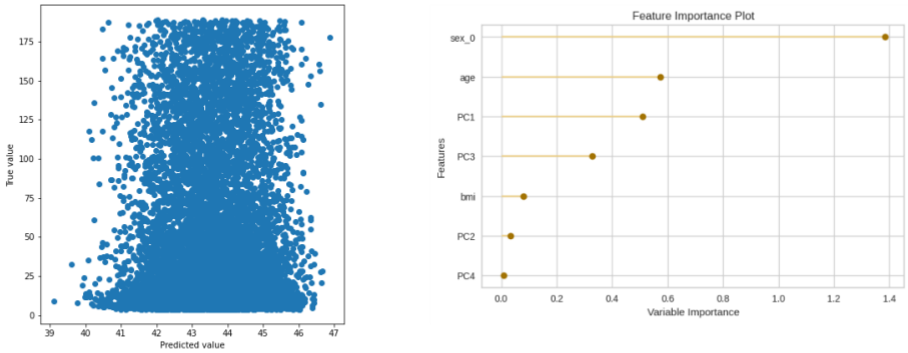

B. Lipoprotein a genes model

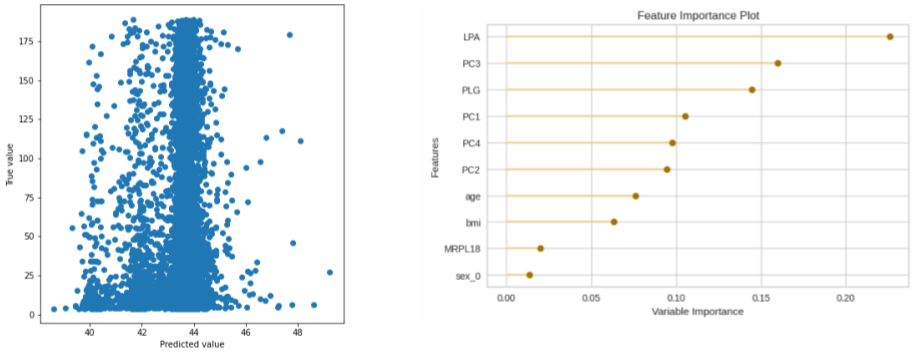

C. Lipoprotein a prs model

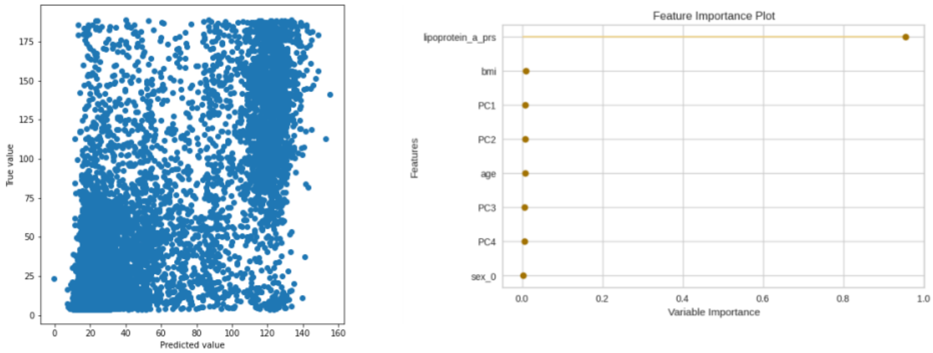

D. Lipoprotein a genes prs model

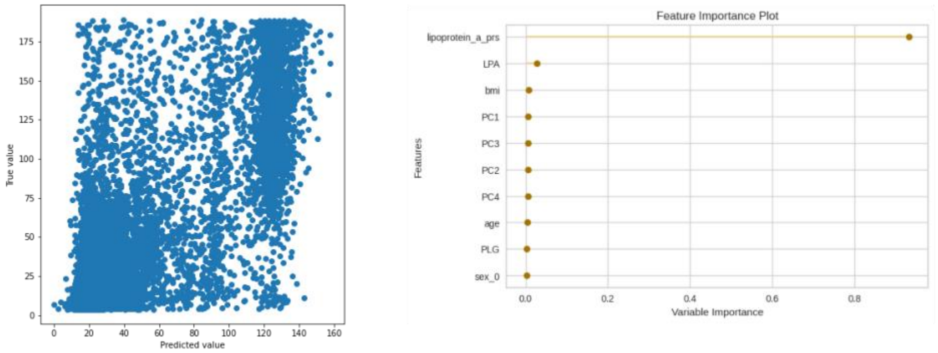

Figure S45 True vs. Predicted value plot (left) and top 10 features (right) for lipoprotein A A. covariates model B. genes model C. PRS model and D. combined model.

A. Phosphate cov model

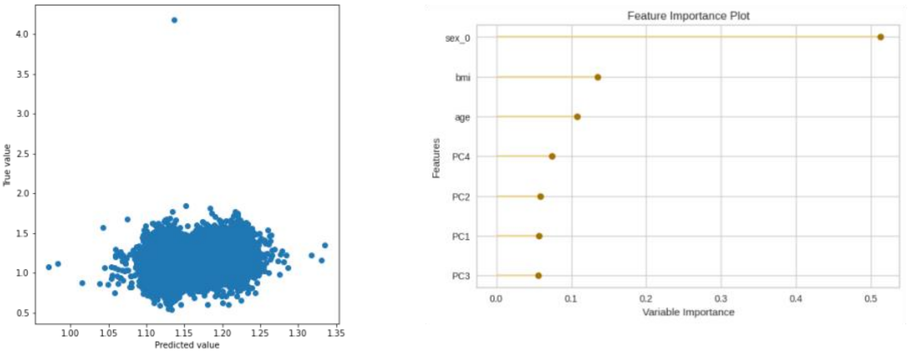

B. Phosphate genes model

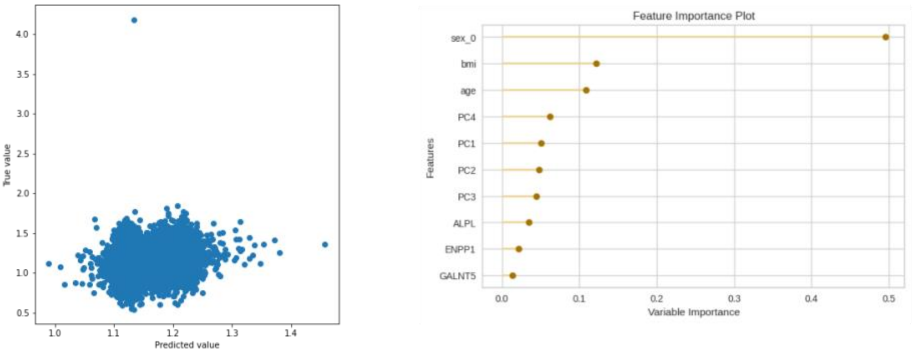

C. Phosphate prs model

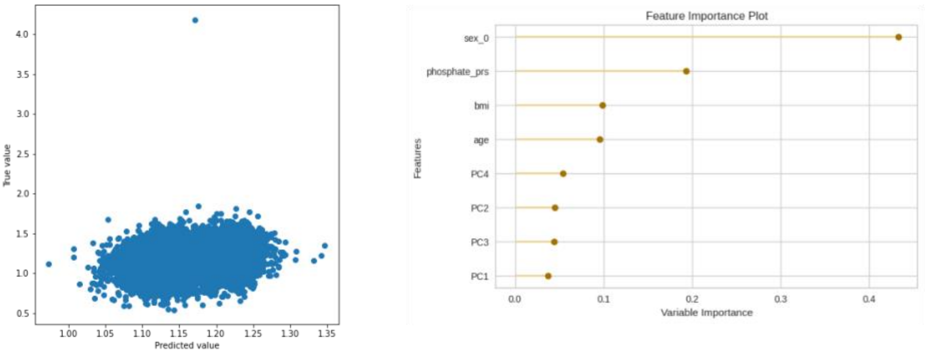

D. Phosphate genes prs model

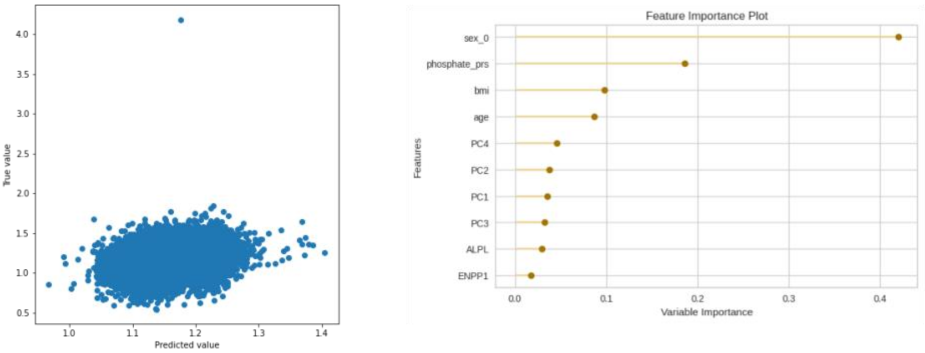

Figure S46 True vs. Predicted value plot (left) and top 10 features (right) for phosphate A. covariates model B. genes model C. PRS model and D. combined model.

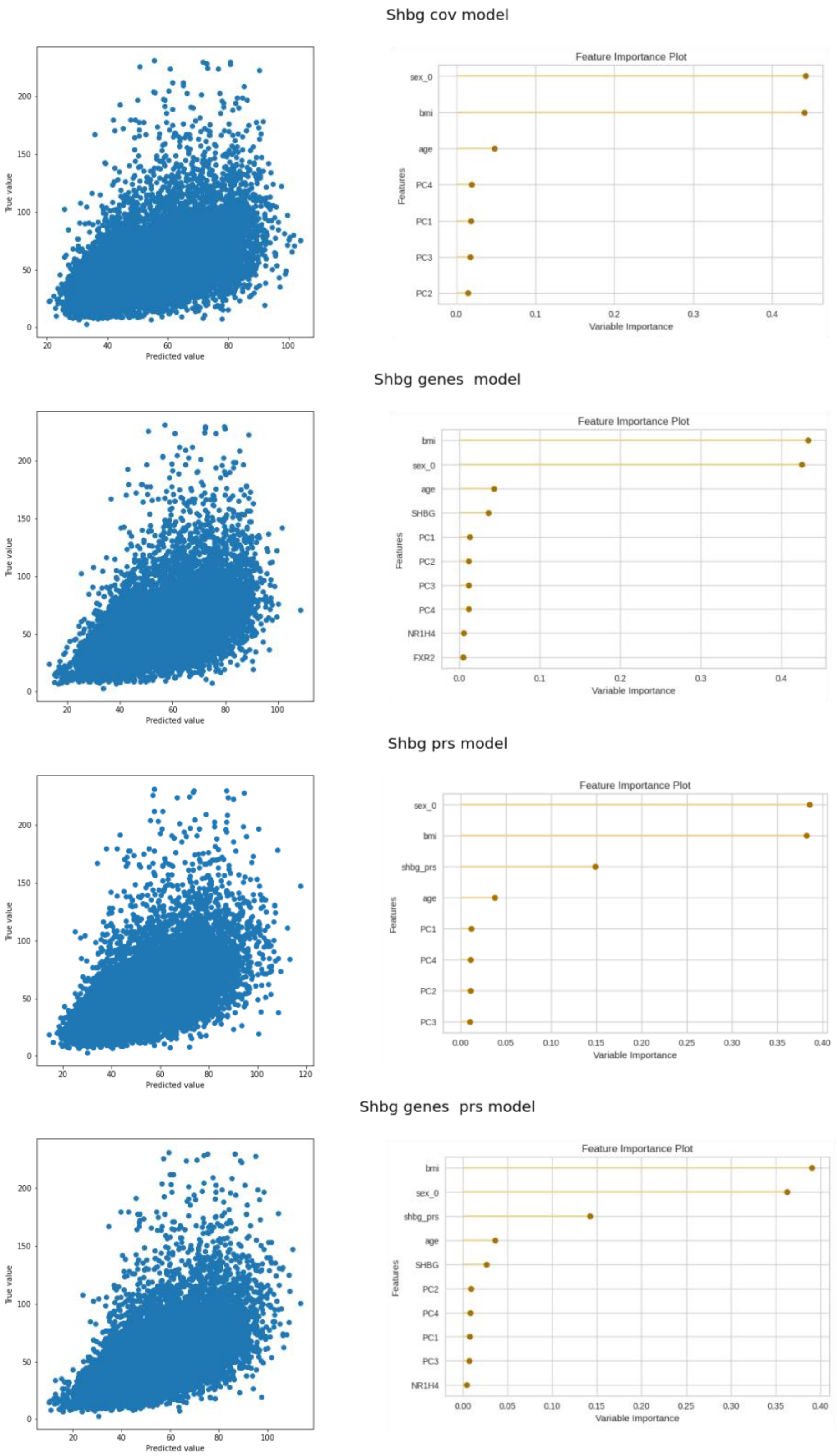

Figure S47 True vs. Predicted value plot (left) and top 10 features (right) for SHBG A. covariates model B. genes model C. PRS model and D. combined model.

A. Testosterone cov model

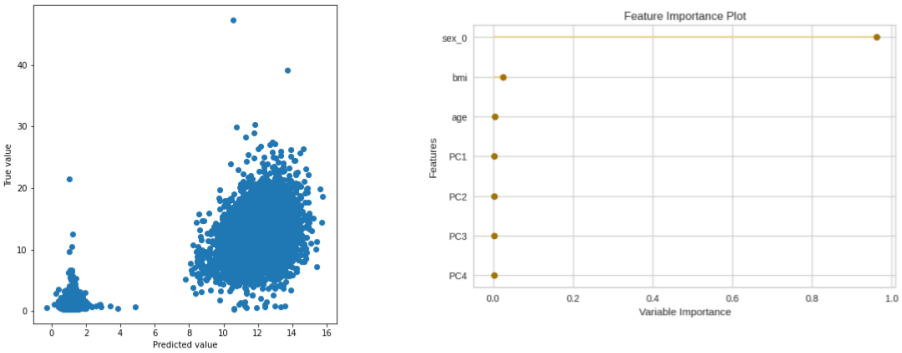

B. Testosterone genes model

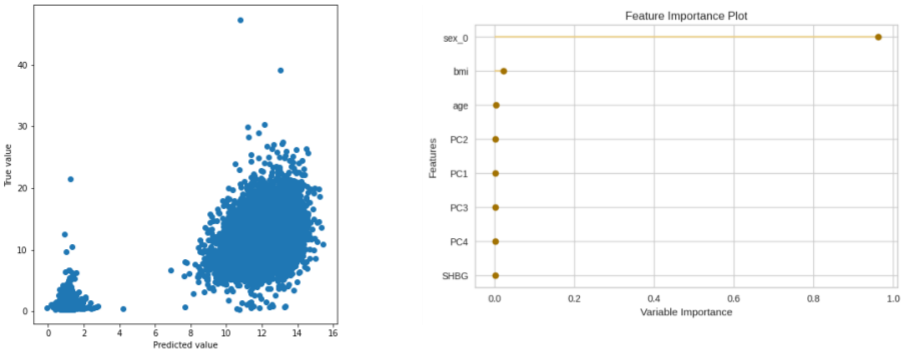

C. Testosterone prs model

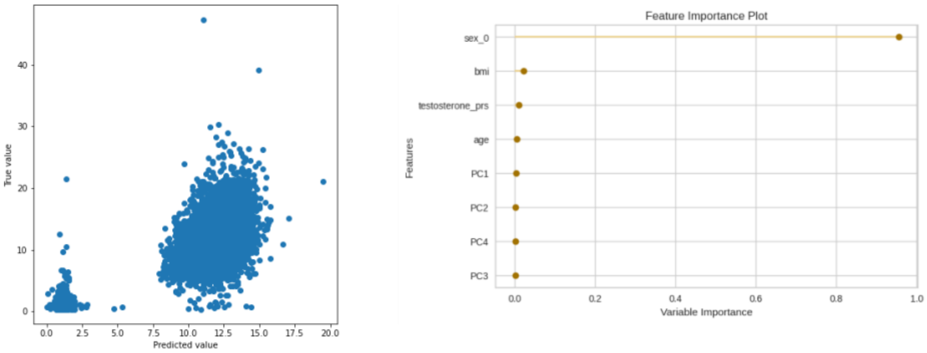

D. Testosterone genes prs model

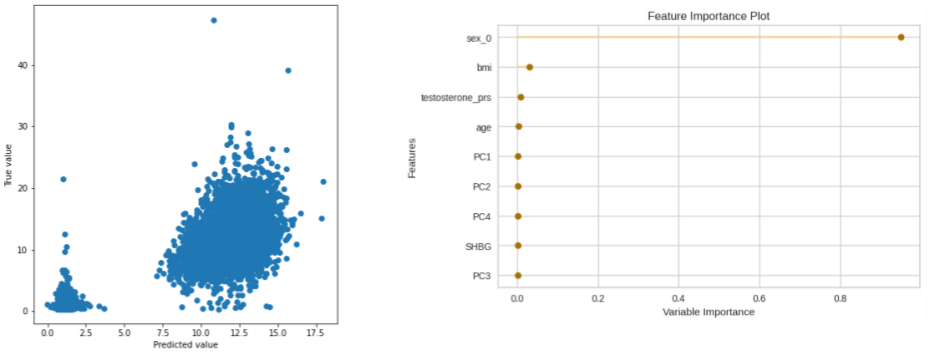

Figure S48 True vs. Predicted value plot (left) and top 10 features (right) for testosterone A. covariates model B. genes model C. PRS model and D. combined model.

A. Total bilirubin cov model

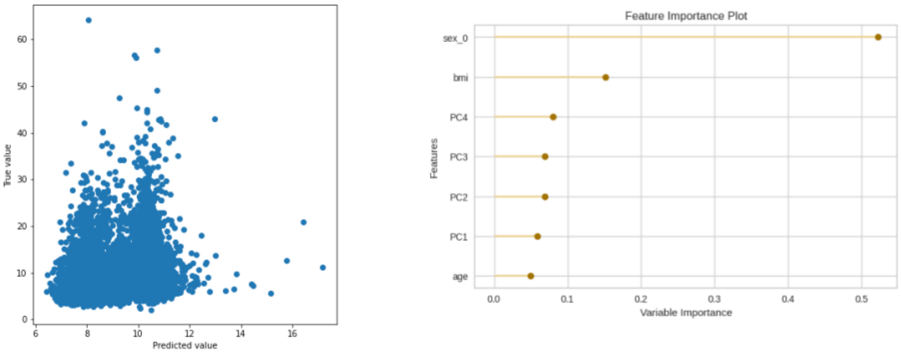

B. Total bilirubin genes model

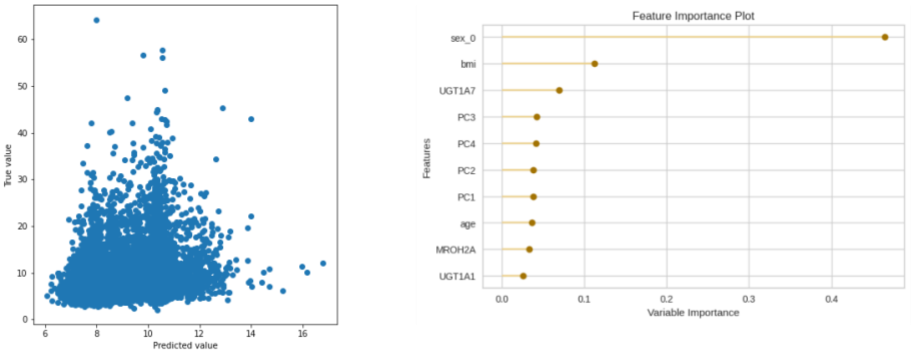

C. Total bilirubin prs model

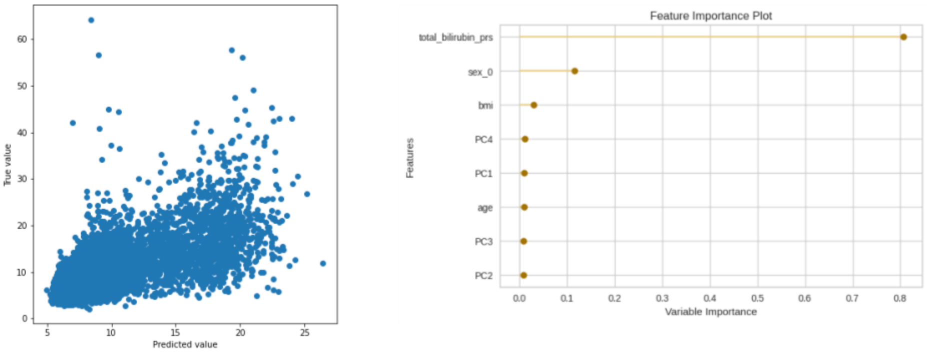

D. Total bilirubin genes prs model

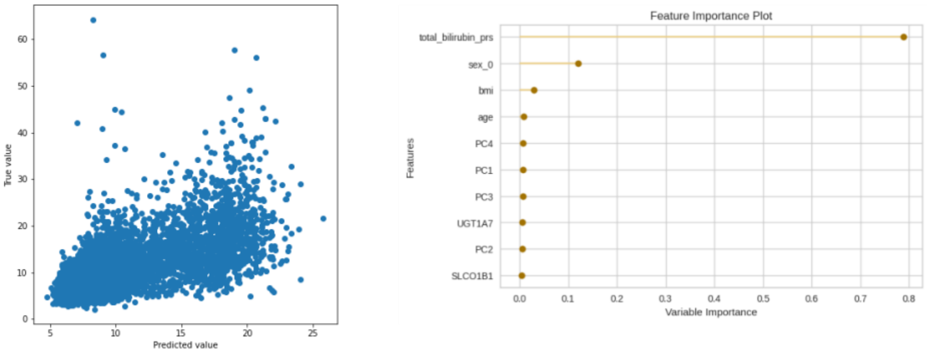

Figure S49 True vs. Predicted value plot (left) and top 10 features (right) for total bilirubin A. covariates model B. genes model C. PRS model and D. combined model.

A. Total protein cov model

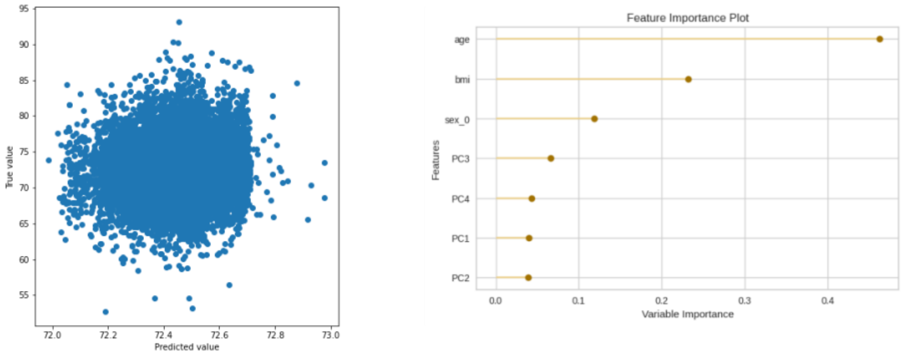

B. Total protein genes model

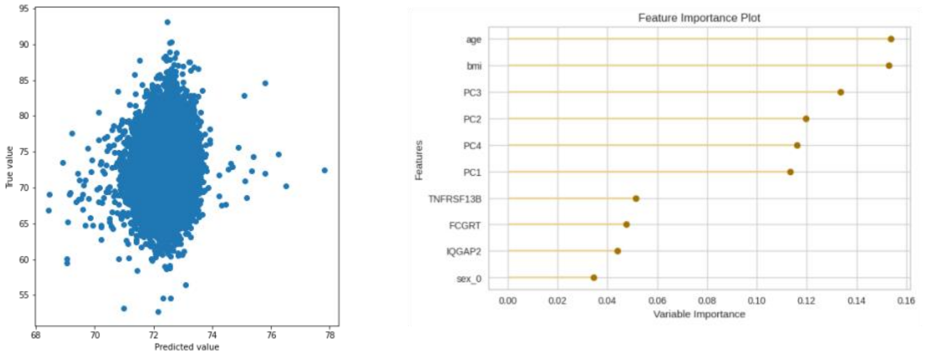

C. Total protein prs model

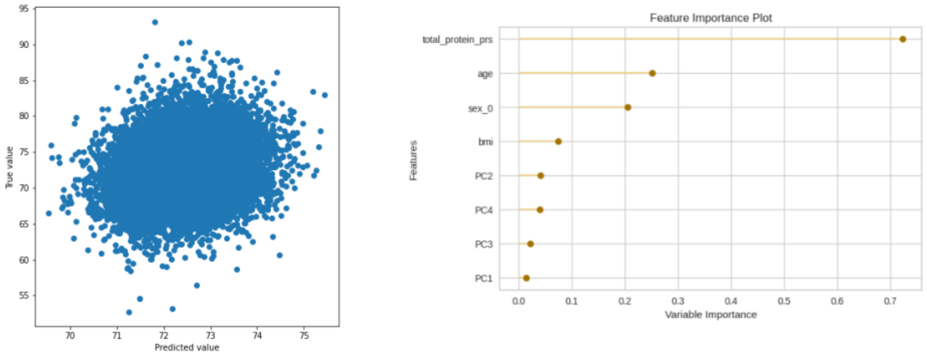

D. Total protein genes prs model

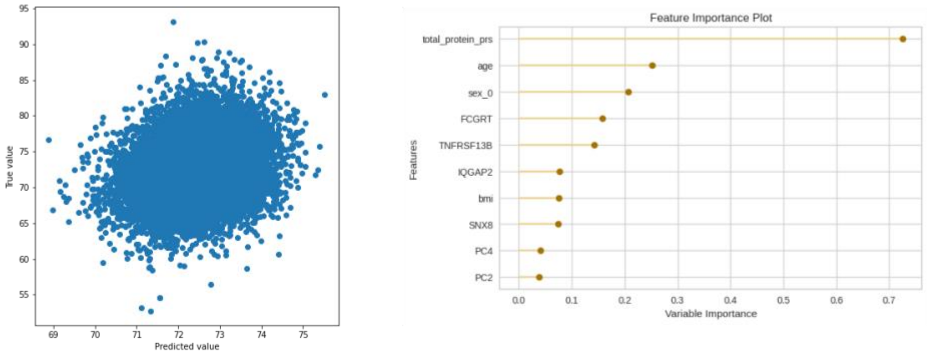

Figure S50 True vs. Predicted value plot (left) and top 10 features (right) for total protein A. covariates model B. genes model C. PRS model and D. combined model.

A. Triglycerides cov model

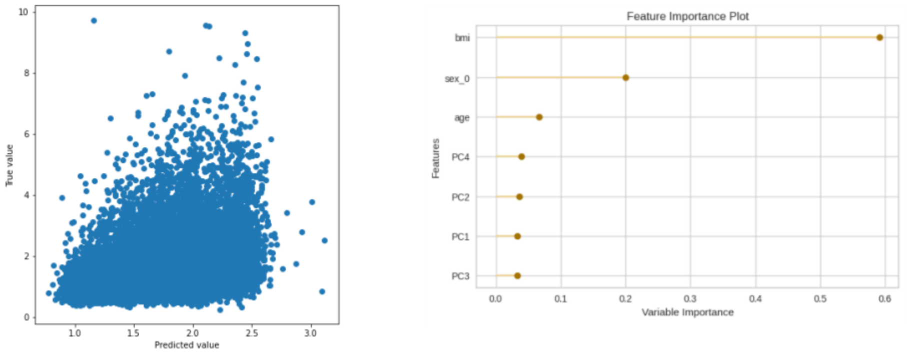

B.Triglycerides genes model

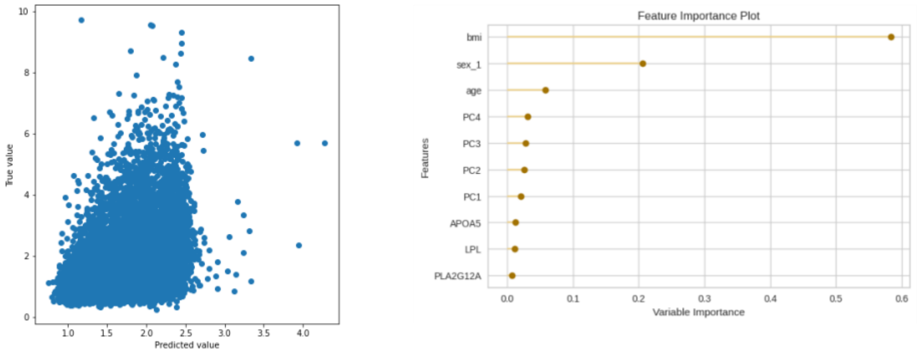

C. Triglycerides prs model

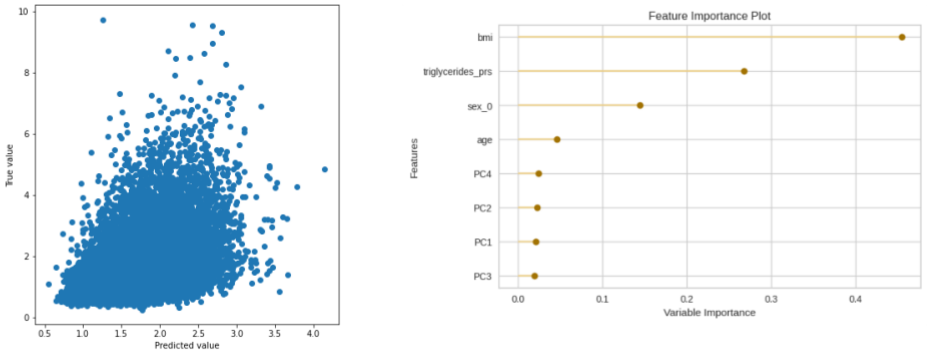

D.Triglycerides genes prs model

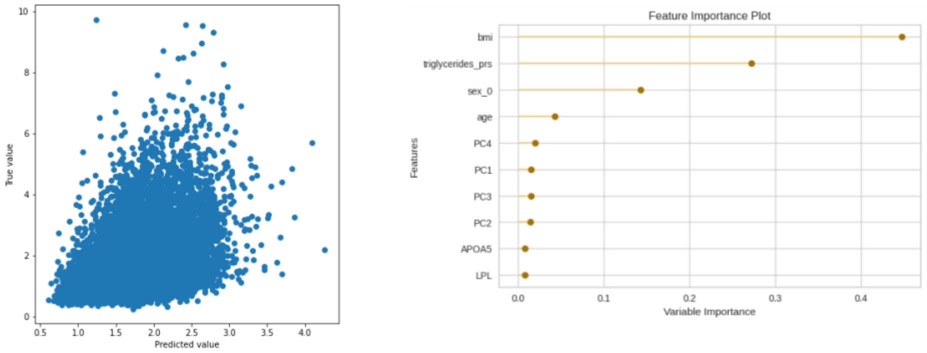

Figure S51 True vs. Predicted value plot (left) and top 10 features (right) for triglycerides A. covariates model B. genes model C. PRS model and D. combined model.

A. Urate cov model

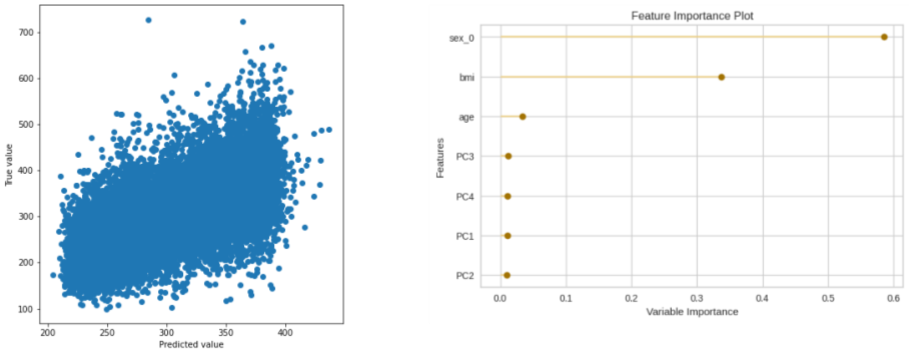

B. Urate genes model

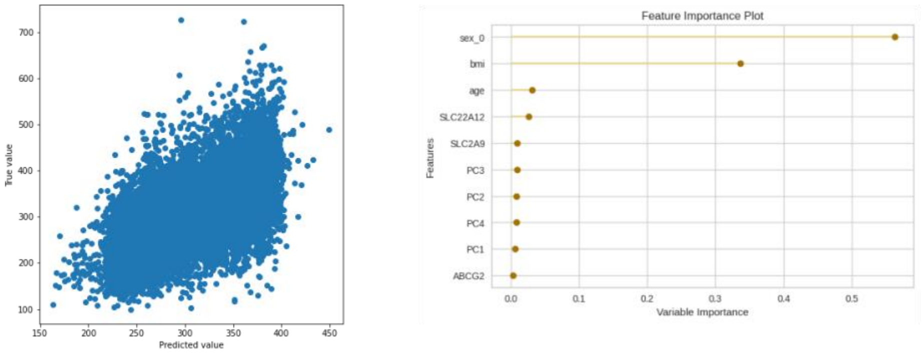

C. Urate prs model

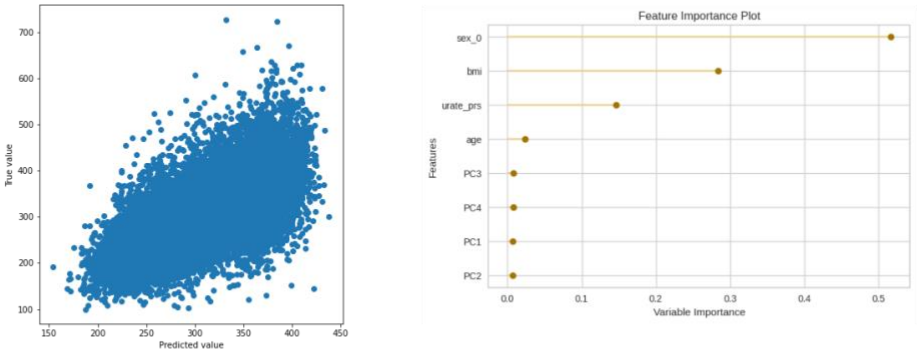

D. Urate genes prs model

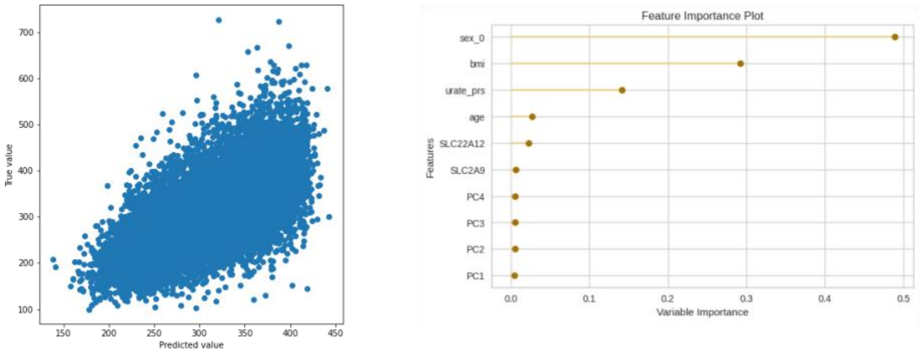

Figure S52 True vs. Predicted value plot (left) and top 10 features (right) for urate A. covariates model B. genes model C. PRS model and D. combined model.

A. Urea cov model

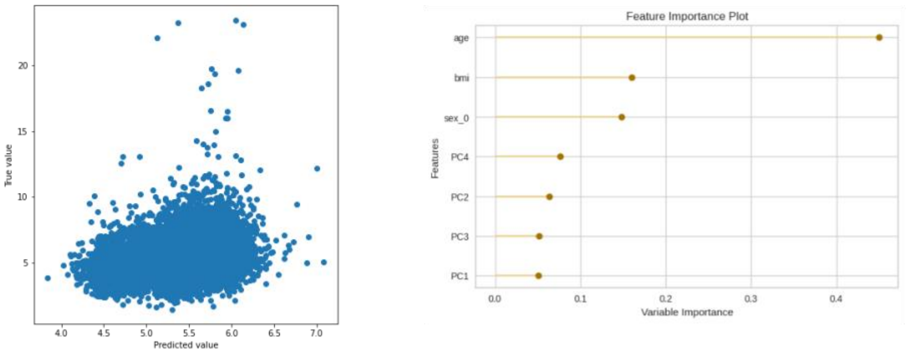

B. Urea genes model

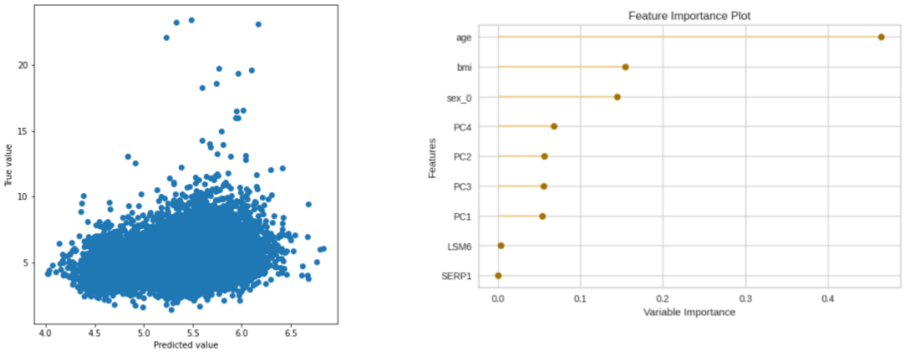

C. Urea prs model

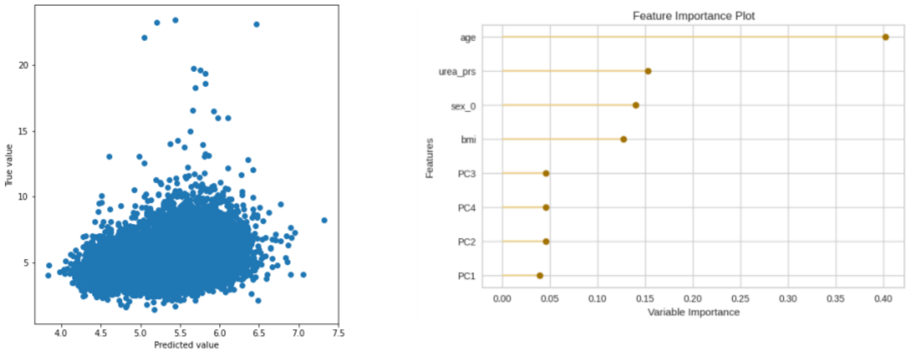

D. Urea genes prs model

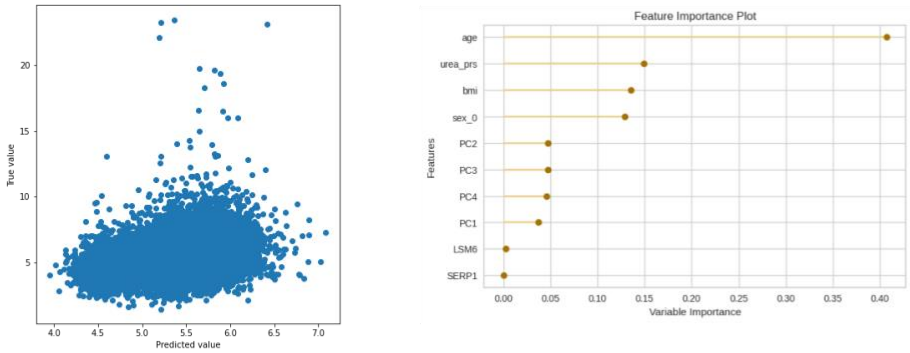

Figure S53 True vs. Predicted value plot (left) and top 10 features (right) for urea A. covariates model B. genes model C. PRS model and D. combined model.

A. Vitamin d cov model

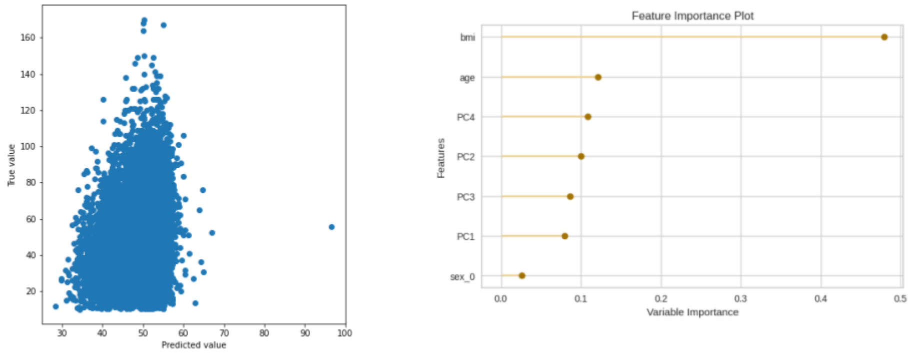

B. Vitamin d genes model

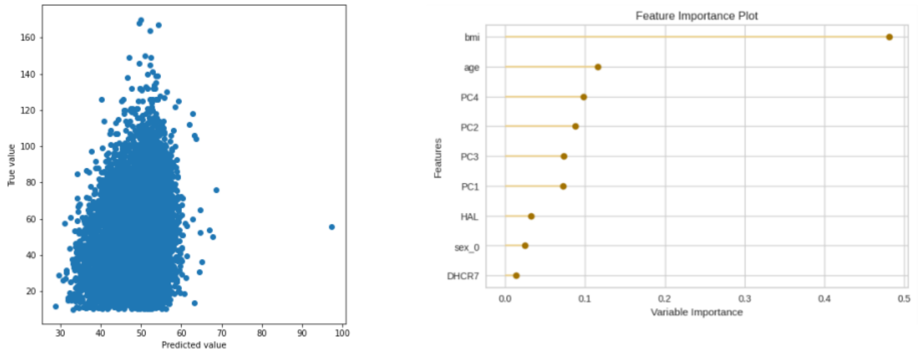

C. Vitamin d prs model

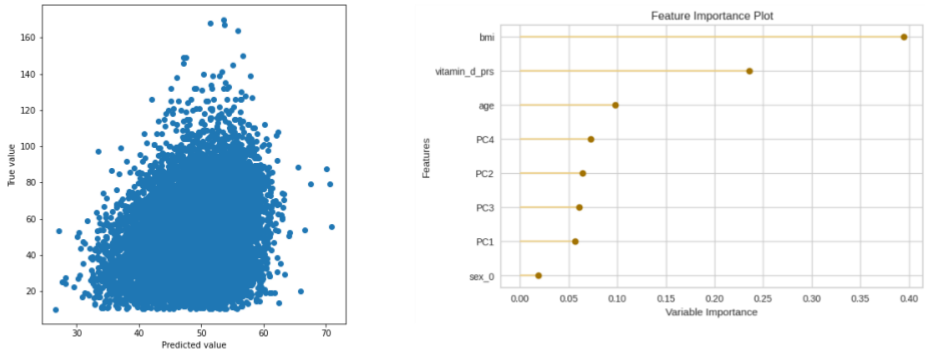

D. Vitamin d genes prs model

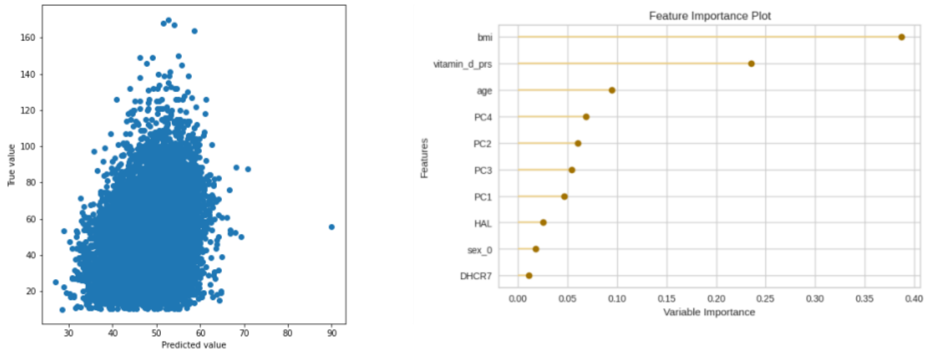

Figure S54 True vs. Predicted value plot (left) and top 10 features (right) for vitamin D A. covariates model B. genes model C. PRS model and D. combined model.
